# Adhesion Failures Determine the Pattern of Choroidal Neovascularization in the Eye: A Computer Simulation Study

**DOI:** 10.1371/journal.pcbi.1002440

**Published:** 2012-05-03

**Authors:** Abbas Shirinifard, James Alexander Glazier, Maciej Swat, J. Scott Gens, Fereydoon Family, Yi Jiang, Hans E. Grossniklaus

**Affiliations:** 1 The Biocomplexity Institute and Department of Physics, Indiana University Bloomington, Bloomington, Indiana, United States of America; 2 Department of Physics, Emory University, Atlanta, Georgia, United States of America; 3 Department of Mathematics and Statistics, Georgia State University, Atlanta, Georgia, United States of America; 4 Theoretical Division, Los Alamos National Laboratory, Los Alamos, New Mexico, United States of America; 5 L.F. Montgomery Ophthalmic Pathology Laboratory, Emory University, Atlanta, Georgia, United States of America; University of Auckland, New Zealand

## Abstract

Choroidal neovascularization (CNV) of the macular area of the retina is the major cause of severe vision loss in adults. In CNV, after choriocapillaries initially penetrate Bruch's membrane (BrM), invading vessels may regress or expand (CNV initiation). Next, during Early and Late CNV, the expanding vasculature usually spreads in one of three distinct patterns: in a layer between BrM and the retinal pigment epithelium (sub-RPE or Type 1 CNV), in a layer between the RPE and the photoreceptors (sub-retinal or Type 2 CNV) or in both loci simultaneously (combined pattern or Type 3 CNV). While most studies hypothesize that CNV primarily results from growth-factor effects or holes in BrM, our three-dimensional simulations of multi-cell model of the normal and pathological maculae recapitulate the three growth patterns, under the hypothesis that CNV results from combinations of impairment of: 1) RPE-RPE epithelial junctional adhesion, 2) Adhesion of the RPE basement membrane complex to BrM (RPE-BrM adhesion), and 3) Adhesion of the RPE to the photoreceptor outer segments (RPE-POS adhesion). Our key findings are that when an endothelial tip cell penetrates BrM: 1) RPE with normal epithelial junctions, basal attachment to BrM and apical attachment to POS resists CNV. 2) Small holes in BrM do not, by themselves, initiate CNV. 3) RPE with normal epithelial junctions and normal apical RPE-POS adhesion, but weak adhesion to BrM (e.g. due to lipid accumulation in BrM) results in Early sub-RPE CNV. 4) Normal adhesion of RBaM to BrM, but reduced apical RPE-POS or epithelial RPE-RPE adhesion (e.g. due to inflammation) results in Early sub-retinal CNV. 5) Simultaneous reduction in RPE-RPE epithelial binding and RPE-BrM adhesion results in either sub-RPE or sub-retinal CNV which often progresses to combined pattern CNV. These findings suggest that defects in adhesion dominate CNV initiation and progression.

## Author Summary

This paper tests hypotheses for the mechanisms of choroidal neovascularization (*CNV*), the pathological growth of capillaries in response to physical defects in a structured tissue, the *retina*, showing that previously neglected cell-cell, cell-ECM and ECM-ECM adhesion failures suffice to determine the loci and progression of neovascularization. Surprisingly, a simple theory based on classes of adhesion failures, which involve variation of only five parameters, can coherently explain the heterogeneous range of CNV growth patterns and dynamics. Our results are generally applicable to other types of tissues where capillaries are close to an epithelium, *e.g.*, lung and gut.

## Introduction

### Organization

We first review the key components of the retina and the processes commonly hypothesized to underlie CNV. We then discuss our main hypotheses for CNV mechanisms and why we believe adhesion may play an important role in both initiation and progression of CNV. We then use a multi-cell computer simulation of a mechanistic computational model of the choriocapillaris, BrM and photoreceptors to investigate the effects of adhesion variations on CNV initiation and progression. Finally, we focus on how adhesion in the BrM-RPE-POS complex changes due to aging and inflammation both in human retina and in animal models of CNV and discuss the biomedical implications of our results.

### Age-Related Macular Degeneration and Pathological Choroidal Neovascularization

*Sprouting angiogenesis*, growth of new blood vessels from preexisting vessels, occurs in response to chemical and mechanical stimuli and to hypoxia in both adult and embryonic tissues. Sprouting angiogenesis requires activation of normally quiescent endothelial cells in pre-existing blood vessels, breakdown of existing basement membranes, migration of activated cells led by one or more endothelial *tip cells* or immune cells (which can also function as tip cells) in response to environmental and cell-contact cues and proliferation of a subset of activated endothelial cells (*stalk cells*) with possible recruitment of support cells (pericytes and smooth muscle cells) during blood-vessel maturation [1].

In pathological angiogenesis, *e.g.* in vascular tumors, vessels do not mature, resulting in leaky capillary vasculature which causes severe edema, inefficient blood transport and reduced oxygenation. Maturation failure can result in a pathological feedback loop, where worsening hypoxia leads to higher levels of proangiogenic factors, including vascular endothelial growth factor A (*VEGF-A*) and platelet-derived growth factor (*PDGF*), and the greater excess of proangiogenic factors produces even more inefficient capillaries, worsening the hypoxia.

The hallmark of *wet* or *exudative* age-related macular degeneration (*AMD*), which is the leading cause of irreversible blindness in North America, Europe, and Australia [2], is lesioning choroidal neovascularization (*CNV*), the invasion of the retina by new blood vessels growing from the choriocapillaris (*CC*). In humans, CNV frequency increases with age, independent of other risk factors or specific insults, though numerous risk factors and insults can greatly increase its probability of occurrence in an individual. CNV in all patients shares the same basic neoangiogenic steps. We distinguish the following three temporal phases: *Initiation*, when endothelial cells first cross Bruch's membrane (*BrM*); *Early CNV*, when endothelial cells spread and form capillaries in a defined locus, and *Late CNV*, when additional loci may become involved, often leading to retinal pigment epithelium (*RPE*) detachment and degeneration, CNV regression/involution and photoreceptor death.

The diverse CNV scenarios are categorized based on histological [3] and clinical observations [4]. Neovascular vessels originating from the choroid can grow in the plane between the RPE and BrM (*sub-RPE*, *occult* or *Type 1 CNV*), between the retina and the RPE (*sub-retinal*, *classic* or *Type 2 CNV*) or in both locations (*combined* or *Type 3 CNV*). Type 3 CNV can also form as a late stage of Early Type 1 or Early Type 2 CNV. In wet AMD, severe visual loss results from subretinal hemorrhage from the leaky CNV, which leads to the eventual formation of a disciform scar. While CNV is generally a disease of the elderly, with onset occurring after 70 years, progression after onset may be rapid. According to the Macular Photocoagulation Study Group [5], about 40% of patients with untreated Type 1 CNV loose significant visual acuity within 12 months. 23% of these patients develop Type 3 CNV within 3 months and an additional 23% develop Type 3 CNV within 12 months. Current therapeutic strategies depend on the CNV locus (subfoveal, juxtafoveal or extrafoveal) and include photodynamic therapy, laser photocoagulation and most-commonly, anti-angiogenic drugs (Lucentis, Macugen or Avastin) [6]. Long-term prognoses are poor; only 20% of patients with Type 1 CNV have stabilized or improved vision 36 months after initial diagnosis and treatment [5].

Developing more effective targeted intervention strategies will depend on understanding CNV mechanisms. However, because of the structural complexity of the normal and diseased retina and the numerous homeostatic and developmental mechanisms operating concurrently, experiments have yet to identify clearly the mechanisms responsible for either CNV initiation or progression. As a novel approach to developing such understanding, this paper applies quantitative models and computer simulations to test hypotheses for the mechanisms leading to CNV initiation and controlling early and late Type 1, 2 and 3 CNV.

### Current Hypotheses for CNV Initiation and Progression

Multiple hypotheses compete to explain CNV initiation, growth and patterning (for comprehensive reviews, see [7], [8]). These hypotheses form two major groups depending on their primary mechanism of action: 1) VEGF overexpression, and 2) irregularities in BrM (including focal defects and basal deposits). Inflammation affects both mechanisms because it promotes formation of irregularities in BrM and participates in angiogenesis. To better understand CNV, we consider its risk factors and correlated pathological conditions and briefly discuss their relationship to the steps of angiogenesis.

Excessive expression of VEGF, mainly in response to injuries and hypoxia, without balancing expression of angiogenesis inhibitors is a major stimulator of neoangiogenesis in most tissues. Excess VEGF has been considered a primary cause of CNV [9] because anti-VEGF drugs can significantly inhibit CNV progression. This hypothesis seems reasonable, because activation, proteolytic activity and survival of ECs depend on VEGF concentrations, and directional migration of tip cells depends on gradients of VEGF, which in turn depend on the composition of the ECM and the proteolytic activity of ECs [10] (in many cases VEGF-A is bound to the ECM and is only sensed by ECs when released by proteolytic enzymes). In animal models, excess secretion of VEGF by the RPE due to subretinal injections of reactive oxygen species (*ROS*) [11] or adenovirus [12] can induce CNV. However, other studies in transgenic mice show that increased expression of VEGF-A and/or angiopoietin-2 in RPE is **not sufficient** to initiate CNV and that overexpression of VEGF can only initiate CNV when combined with subretinal injections [13] which disturb the integrity of the RPE, probably by triggering inflammation, reducing RPE-POS contact adhesion and inducing RPE growth [14]–[16]. Also in transgenic mice [17], overexpression of VEGF-A_164_ in the RPE causes extensive intrachoroidal neovascularization, but does not lead to sub-RPE or sub-retinal CNV, again suggesting that an intact Bruch's membrane/RPE barrier prevents choroidal neovascularization from penetrating into the subretinal space. Immunohistochemical analyses of human subjects with a history of chorioretinal disease show that compared to age-matched control subjects (mean age about 80 years) their PEDF levels are significantly lower than their VEGF levels in RPE cells, the RPE basal lamina, BrM and the choroidal stroma [9], [18]. This imbalance shows that disturbed angiogenic and antiangiogenic factor levels correlate with late-stage chorioretinal disease, but the relative expression levels of these factors would need to be measured experimentally **before** CNV initiation to establish whether the imbalance is a cause or a result of CNV. However, secreted proteome profiling in human RPE cell cultures derived from donors with AMD shows a 2- to 3-fold increase in their levels of PEDF compared to age-matched healthy donors [19]. Thus, while the experimental evidence shows a clear increase in proangiogenic factors, this increase may be compensated by an increase in antiangiogenic factors. Whether an imbalance develops is thus not definitively established. Another serious objection to the VEGF-hypothesis is that it fails to explain the distinct loci and progression of CNV. VEGF levels are high throughout the retina and cannot provide spatial cues to lead to the localization of CNV to either the RPE-BrM boundary (Type 1) or the RPE-POS boundary (Type 2). Indeed, no experiment has established the presence of VEGF gradients within the BrM-RPE-POS complex.

Both age-related changes of the retina and pathological conditions can increase VEGF expression. Life-long accumulation of lipids in BrM and their gradual oxidation (producing reactive oxygen species and recruiting immune cells) correlate with increased production of VEGF by the RPE and greater likelihood of developing CNV [20]. Hypoxia also temporarily increases the secretion of VEGF in cultured RPE cells by up to 3-fold over 48 hours, followed by a return to baseline [21], [22]. Inflammatory cells also secrete VEGF and other proangiogenic and antiangionegic factors (see *Inflammation* subsection in supplementary Text S1).

Irregularities of BrM include focal breaks and thinning in BrM, abnormal production of ECM by the RPE, and formation of soft drusen. All of these BrM defects correlate with CNV [23], [24]. However, the common hypotheses that BrM presents a physical barrier to the invasion and/or growth of choriocapillaries into the retina and that small gaps in BrM may be responsible for initiation of CNV, contradict several experimental and clinical observations: 1) BrM is never an impenetrable barrier to immune and tip cells and has not been shown to physically block the invasion of activated ECs into the sub-RPE space. BrM is only 2–4 µm thick, with pores up to 0.5 µm diameter [25]. The BrM elastin layer in the macula of healthy young adults (age<62) can have gaps of up 2 µm [26]. 2) Activated endothelial cells, which are always present in the normal choriocapillaris, probe their micro-environment by sending out processes (like filopodia) as thin as 0.1 µm and as long as 200 µm even in dense embryonic and adult tissues. Such flilopodial processes can easily cross BrM through its pores. Leukocytes can cross BrM rapidly under both normal and inflammatory conditions [27] (taking at most a few hours to cross the BrM-RPE barrier and only a few minutes to cross the endothelium in an *in vitro* flow model [28]). ECs digest and penetrate an intact BrM in less than a week when RPE is severely damaged due to phototoxicity in a rat model [29]. 3) The rate of CNV in persons younger than 50 years old is negligible (except in cases of excess inflammatory response in the eyes). 4) The CNV initiation probability when BrM is mechanically disrupted in animal models is about 10% [30]. These observations suggest that focal defects and thinning of BrM do not significantly reduce the already minimal efficacy of the physical barrier function of healthy BrM. Instead, other mechanisms may explain the correlation of focal defects in BrM with CNV, *e.g.*, breaks in BrM due to calcification may disrupt both the RBaMs and the basement membranes of CC cells, disrupting the epithelial junctional structure of the RPE [23] and activating CC endothelial cells. This simultaneous activation of ECs and disruption of the RPE may explain the correlation of focal defects in BrM with CNV.

However, while BrM does not form a mechanical barrier to persistent EC penetration of the retina, BrM and the RPE attached to it clearly do form an effective barrier to choroidal penetration, even in the presence of small holes in BrM. The nature of this barrier is not clear. Haptotaxis may play a role. ECs exhibit strong haptotactic preference for their own basement membrane. The basement membrane of the RPE (RBaM) differs in structure and components from the CC basement membrane (CC BaM) (reviewed in [31]). ECs preferentially adhere to their own basal lamina and new blood vessels follow the pattern of any pre-existing EC-manufactured basal lamina after capillary atrophy [32]. Thus, in the absence of factors that induce directed migration of ECs (chemotaxis or haptotaxis), we hypothesize that activated ECs of the choriocapillaris prefer to stay on the outer side of BrM, which has a high level of CC BaM, and not to invade sub-RPE space, which almost entirely lacks CC BaM.

Since overexpression of VEGF and reduction in BrM's barrier function may not fully explain CNV initiation, multiple types, loci and progression [23], this paper investigates the possible role of additional mechanisms, particularly adhesion defects.

### Adhesion Failure and CNV

While not usually considered crucial to CNV, a great deal of experimental evidence suggests that failures of adhesion are essential for the development of CNV (Table 1). The strict spatial separation of the CC from the normal retina and the distinct loci of Type 1 and Type 2 CNV suggest that the physical structure and properties of the BrM-RPE-POS complex may determine both CNV initiation and progression. Experimental evidence suggests that reduced RBaM-BrM adhesion may enable CNV to invade the sub-RPE space. The differences in properties and effects of hard and soft drusen support this hypothesis. Soft drusen are strong risk factors for CNV and are often associated with detachment of the RPE from BrM, suggesting that they substantially reduce RBaM-BrM adhesion [33]–[35]. However, hard drusen, which contain hyalinised material and attach firmly to the inner collagenous layer of BrM (based on EM images) do not greatly increase the likelihood of CNV [24], [35]. Softening of hard drusen, which reduces their adhesion to RBaM, correlates with CNV [36], [37]. Similarly, the ability of inflammation to induce CNV suggests that impaired lateral adhesion between cells in the RPE promotes Type 2 CNV. Pathological conditions that compromise the integrity of the oBRB by weakening junctional epithelial adhesion in the RPE cause a wide range of neovascular diseases in the retina [38]–[42]. Recent studies show that subretinal drusenoids, which are drusen-like deposits that accumulate between the RPE and photoreceptors, perturb RPE-POS adhesion and correlate with CNV [43]–[45]. Finally, the detachment of the POS from the RPE (retinal detachment) reduces the integrity of the oBRB and significantly increases the risk of CNV in animal models of CNV, suggesting that impaired RPE-POS adhesion also promotes CNV.

**Table 1:** Pathological conditions and injuries, their effects on adhesion and their correlations with CNV.

	Context		Adhesion			Effects		
	Condition	Subject	RPE-RPE	RBaM-BrM	RPE-POS	RPE Viability	CNV Loci	Simulation Results
1	Normal Aging (No Drusen)	Human	+	+	+	+	-	No initiation even in presence of small holes in **BrM** (Table S1)
2	Hard Drusen [23], [35]	Human	−	−	+	−	-	See the *Current Hypotheses for CNV Initiation and Progression* section
3	Soft Drusen [23], [35]–[37]	Human	−/+	−/−	−/+	−/+	Sub-RPE	**S11**, **T12**, **P13 CNV** (Tables S2, S5, S6, S7)
4	Sub-retinal Drusenoid (reticular pseudodrusen) [44], [45], [96]	Human	−/−	−/−/+	−/−	−/+	Sub-Retinal and/or Sub-RPE	**T12, S22, P23 CNV** (Tables S6, S8, S9)
5	BrM Calcification [23], [24]	Human	−/+	−/+	+	−/+	*	*
6	Active Inflammation [80]	Young Human	−	+	−/−/+	−/+	Sub-Retinal	**S22 CNV** (Table S8)
7	Retinal Detachment [14], [40]	Cat	−/−	+	−	+	*	**S22 CNV** (Table S8)
8	High Fat Diet+Aging+Blue Light [75]	Mouse	−/+	−/−	+	+/−	Early Sub-RPE	**ET1 CNV** (Table S2)
9	Chemotoxicity [81]	Rabbit	−	−/+	−	−	Sub-Retinal	**P23 CNV** (Table S9)
10	Sub-Retinal Injection [83]–[86]	Rat, Rabbit	−	−/+	−	−/+	Sub-Retinal	**S22**, **P23 CNV** (Tables S8, S9)
11	Sub-retinal Injection and VEGF Overexpression [13]	Rat	−	−/+	−	−/+	Sub-Retinal	**S22**, **P23 CNV** (Tables S8, S9)

**Columns: Condition**: type of condition, injury or perturbation in clinical or experimental observations. **Subject**: human or animal. **Adhesion**: strength of adhesion (+ = normal, − = moderately impaired, − = severely impaired). **Effects**: RPE viability (+ = most RPE cells remain viable, − = some RPE cells die, − = most RPE cells die), CNV loci (− = no or low probability of initiation and progression, sub-RPE = Type 1, sub-retinal = Type 2, combined pattern = Type 3, * = no data presented/available). Simulation results (**boldface** words = model objects. **CNV** Type definitions: see Table 4 and Table 5. * = no data presented/available. See the *Results* and *Discussion* sections for details of simulation results).

Since the relative importance, roles and interactions among the different types of adhesion impairment during CNV initiation and progression are unclear, this body of experimental evidence motivated us to study the role of adhesion failures in the BrM-RPE-POS complex in CNV.

#### The need for models and simulation

While a detailed experimental analysis of adhesion effects in CNV is desirable, it is currently impractical. No animal model exhibits the full range of AMD-related CNV pathologies [46], while *in vitro* experiments do not reproduce the complex layering and porosity of BrM, the interlocking of the RPE and POS, the accumulation of lipids in the BrM layers or the formation of soft drusen. Independent quantitative control of biological mechanisms is experimentally difficult, especially *in vivo*. We therefore chose to develop computational models which allow us to titrate the effects of specific mechanisms without confounding crosstalk or quantitative uncertainties and to study the synergistic or antagonistic effects of multiple mechanisms acting simultaneously or sequentially. Computational models also allow us to explore many more combinations of bio-mechanistic hypotheses and parameter choices than we could in experiments. Our computational models include key retinal components, cell-cell, cell-ECM and ECM-ECM adhesion mechanisms and major angiogenesis-related processes, like BrM breakdown by proteases, hypoxic signaling upregulating VEGF production and VEGF and oxygen transport. Our computational model of the retina allows us to investigate the significance of these hypothesized mechanisms in both CNV initiation and progression. In this paper, we focus on the significance of adhesion failures in the BrM-RPE-POS complex to CNV early and late progression, deferring a detailed comparative study of the roles of VEGF overexpression and BrM defects to future publications.

### A Quantitative Model of the Retina-RPE-CC Complex

CNV involves the interaction of two complex components, the retina, with its supporting structures, and the choriocapillaris. We briefly review the functional and structural properties of these components in the context of CNV in supplementary Text S1.

To allow unbiased study of CNV mechanisms, our model of the retina-RPE-CC includes objects and processes (Table 2) capable of recapitulating all the major CNV hypotheses (VEGF overexpression, BrM defects, adhesion failures and inflammation). We list our key modeling assumptions in supplementary Text S2. We translate our quantitative model into a computational model in the *Methods* section in supplementary Text S3 (for a detailed explanation of our modeling terminology see supplementary Text S4). To avoid confusion, we use normal fonts for biological objects and **boldface** to represent objects and times in the quantitative model, *e.g.* **RPE** denotes the model's representation of biological RPE and one **year** denotes one simulated biological year. We also use **boldface** to distinguish the specific simulation interactions of **junctional adhesion**, **labile adhesion** and **plastic coupling** in our model from their biological correlates, but we do not use a separate font to distinguish other modeled and biological processes.

**Table 2:** Model objects and processes.

Object Types			Processes
**Generalized Cells**	**Endothelial Cells (EC)**	**Vascular Cells** (of the **CC**)	Adhere via **junctional adhesion** to **ECs** and **BrM**
			Adhere via **labile adhesion** to **RPE**, **PIS**, **POS** and **Medium**
			Take up **RPE-derived VEGF-A**
			Secrete **short-diffusing VEGF-A**
			Secrete **Oxygen**
			Die when **RPE-derived VEGF-A** is less than a threshold
			Have intrinsic random motility
			Migrate via chemotaxis up gradients of **short-diffusing VEGF-A** (Contact-inhibited)
		**Stalk Cells** (of the **CNV**)	All Processes of **Vascular Cells** and:
			Migrate via chemotaxis up gradients of **RPE-derived VEGF-A** (Contact-inhibited)
			Grow in response to **RPE-derived VEGF-A** (Contact-inhibited)
		**Tip Cells**	All Processes of **Vascular Cells** except secretion of **Oxygen**:
			Migrate via chemotaxis up gradients of **RPE-derived VEGF-A** (Contact-inhibited)
			Secrete **MMP**
		**RPE Cells**	Adhere via **junctional adhesion** to **RPE cells** and **BrM**
			Adhere via **labile adhesion** to **ECs, PIS, POS** and **Medium**
			Secrete **RPE-derived VEGF-A**
			**Die** in absence of contact with **RPE** and **BrM**
			Have intrinsic random motility
	**Photoreceptor Compartments**	**POS Cell-parts**	Adhere via **junctional adhesion** to **POS** and **PIS cells**
			Adhere via **labile adhesion** to **ECs, RPE, BrM** and **Medium**
			Have intrinsic random motility
		**PIS Cell-parts**	All Processes of **POS Cell-parts** and:
			**Consume Oxygen**
	**Extracellular Materials**	**BrM**	Non-diffusing solid material (implemented as non-motile generalized **cells**)
			Adheres via **labile** and/or **junctional adhesion** (see **EC**, **RPE**, **PIS**, **POS**)
			Degraded by **MMP**
		**Medium**	Adheres via **labile adhesion** to **cells** and **BrM**
			Fills space unoccupied by **cells** or **BrM**
**Fields**		**Oxygen**	Diffuses
			Decays
		**RPE-derived VEGF-A**	Diffuses
			Decays
		**Short-diffusing VEGF-A**	Diffuses
			Decays
		**MMP**	Diffuses
			Decays
			Degrades **BrM**

#### Anatomical components of the model

**Figure 1:**
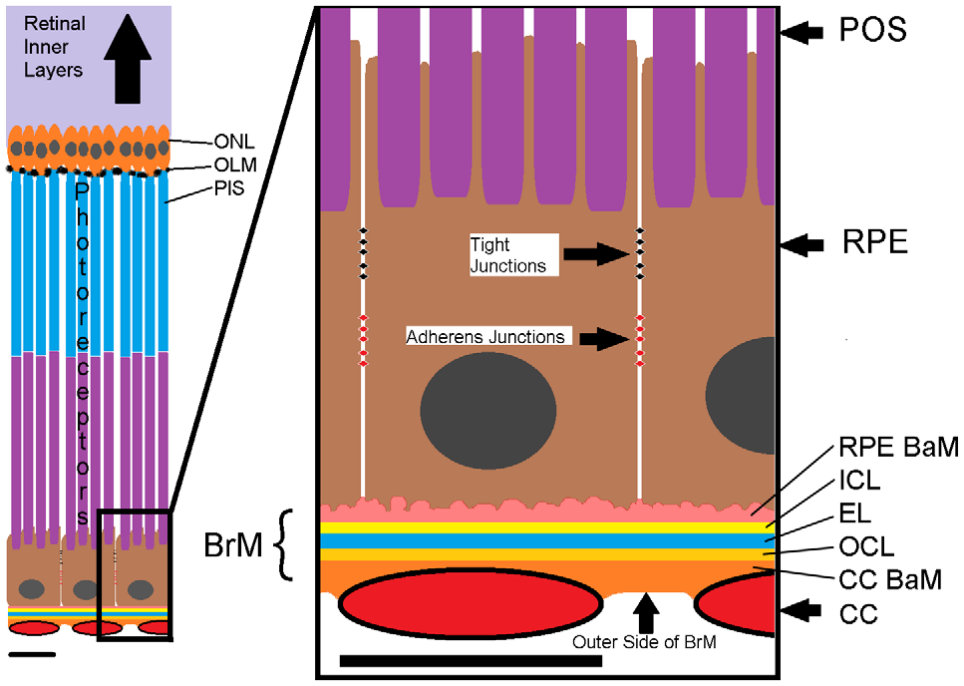
Retinal structure, the retinal pigment epithelium, Bruch's membrane and the choriocapillaris. *Left large-scale*: Structure of the outer retinal layers, the RPE and the CC. *Right*: Detail of the CC-BrM-RPE-POS complex. *CC*: choriocapillaris, *BrM*: Bruch's membrane, *RPE*: Retinal pigment epithelium, *CC BaM*: Basement membrane of the CC, *OCL*: Outer collagenous layer, *EL*: Elastin layer, *ICL*: Inner collagenous layer, RPE BaM: Basement membrane of the RPE (we abbreviate RPE BaM as *RBaM*), *POS*: Photoreceptor outer segment, *PIS*: Photoreceptor inner segment, *ONL*: Outer nuclear layer. Light purple shading indicates the location of the inner retina. Scale bars ∼10 µm.

Since CNV is usually limited to the outer retina, we model the choriocapillaris, BrM, RPE and parts of the outer retina in detail, and represent the inner retina implicitly through appropriate boundary conditions at the *outer limiting membrane* (see Figure 1, for normal anatomical components). Our model does not explicitly represent the OLM which defines the innermost (towards the inner retina) **boundary** of the modeled outer retina. The properties of the retinal layers depend on the in-layer distance from the fovea. We could represent these typical anatomical/thickness variations of the biological retina in our model by changing a limited number of geometrical and metabolic parameters, though we do not do so in the present paper. Our model explicitly represents **BrM**, but neglects its layered structure and assumes that the inner and outer basal laminae and basement membrane of **BrM** provide equivalent adhesion substrates. Modeled **BrM** is composed of small blocks of non-diffusible solid material (frozen generalized **cells**). We assume that **cells** cannot cross intact **BrM**. So our modeled **BrM** blocks **cell** migration (See *Discussion* section for more details). Our model does not explicitly represent basal deposits, which play a major role in CNV initiation and progression, but includes them implicitly via their effects on the adhesion properties of the RPE-BrM complex. Since CNV originates from the outgrowth of capillaries in the choriocapillaris, our model represents the capillary network of the **choriocapillaris** (***CC***) and the **endothelial cells** (***EC*** **s**) explicitly. Modeled **CNV** capillaries are composed of **stalk cells** (see the *Angiogenesis and BrM Degradation* subsection, below). Our model represents extracellular fluid in the tissue by a **medium** that fills spaces unoccupied by **cells** or **BrM**.

#### Oxygen transport and metabolism in the model retina

The **choriocapillaris** secretes diffusing **oxygen** at a constant rate (see the *Methods* section in supplementary Text S3) and PO_2_ at the OLM **boundary** is constant. Numerous experimental and theoretical studies of oxygen tension profiles in the retina show that the oxygen consumption by the RPE is negligible compared to that of the PIS [47]. Our model assumes that **RPE** oxygen uptake is negligible [47], [48]. Our model neglects the effects of blood flow entirely and assumes that PO_2_ is independent of position along a capillary, or whether a capillary is a sprout or has anastomosed with other vessels. The effects of blood flow on vascular remodeling and tumor growth have been extensively studied by Owen *et al.* [49], Szczerba and Székely [50], Perfahl *et al.* [51], Alarcon *et al.*, Bartha and Rieger [52], Welter *et al.* [53], [54], McDougall *et al.* [55], Stephanou *et al.* [56], [57], Pries *et al.* [58]–[61] and Macklin *et al.* [62]. Our flow-related simplifying assumptions generally have the effects of increasing oxygen availability, reducing the rate and extent of neovascularization. We have performed simulations (data not shown) in which PO_2_ at **choriocapillaris** is set to half of its normal level, representing continuous systemic hypoxia. These simulations show that lower PO_2_ at the **choriocapillaris** has little effect on the generic behavior of our model.

#### Adhesion properties of EC, RPE, POS and PIS cells

Our model has two types of **cell-cell** and **cell-BrM** adhesion: 1) ***labile adhesion*** and 2) ***junctional adhesion***. Modeled **labile adhesion** represents cell-cell or cell-ECM surface adhesion in the absence of strong junctional structures (*e.g.* RPE-POS adhesion). **Junctional adhesion** combines **labile** adhesion at **cell** boundaries with **plastic coupling** (*e.g.* between neighboring **cells** or between **BrM** and **cells**). The **plastic coupling** simulates cytoskeletally-coupled junctional structures as breakable springs (see the *Methods* section in supplementary Text S3) that mechanically connect neighboring **cells** and also connects **cells** to **BrM**. **Junctional adhesion** represents biological epithelial/endothelial junctional adhesion or cell-ECM focal adhesion. Our representation of adhesion gives us the flexibility to represent both mesenchymal cells and cells organized in an epithelium. When **plastic coupling** between neighboring or ECM-adhering **cells** is strong relative to other effects including **labile adhesion**, **cells** are less likely to break their **plastic couplings** and change their neighbors, as is typical in epithelial-junction-coupled cells in an epithelium (*e.g.* a layer of differentiated epithelial cells *in vitro* at 100% confluency). However, when **plastic coupling** is weak or absent, **cells** can relatively easily migrate or change their neighbors, as is typical of mesenchymal cells or epithelial cells *in vitro* well below 100% confluency. In our model, we use this flexibility to vary the strengths of **labile adhesion** and **plastic coupling** independently to represent the differing adhesion properties of cells in healthy and pathological tissues.

**Figure 2:**
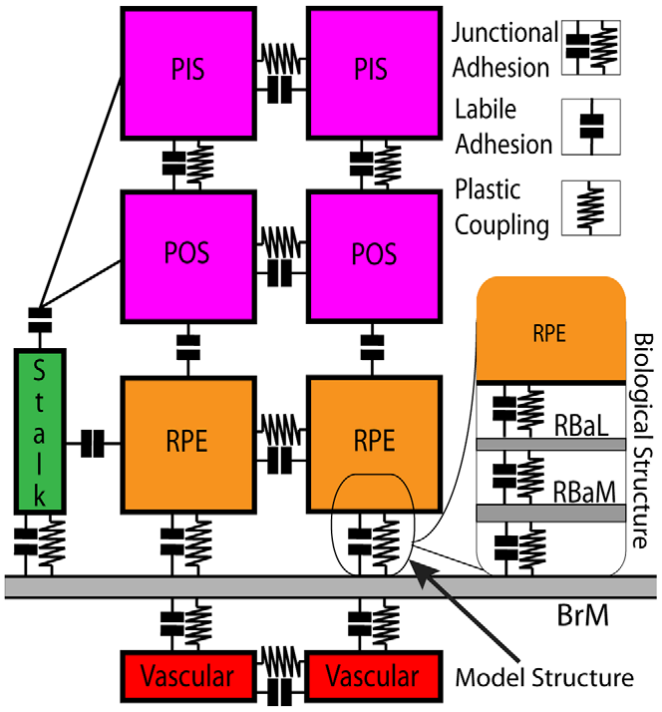
Adhesive interaction processes in the model retina. Our model includes two types of **cell-cell** and **cell-BrM** adhesion: 1) ***labile adhesion*** and 2) ***junctional adhesion***. Modeled **labile adhesion** represents cell-cell or cell-ECM labile adhesion in the absence of strong junctional structures (*e.g.*, RPE-POS adhesion). **Junctional adhesion** combines **labile adhesion** at **cell** boundaries with **plastic coupling** (*e.g.*, between neighboring **cells** or between **BrM** and **cells**). **Plastic coupling** simulates cytoskeletally-coupled junctional structures as breakable springs (see the *Methods* section in supplementary Text S3) that mechanically connect neighboring **cells** and also connect **cells** to **BrM**. **Junctional adhesion** represents biological epithelial or endothelial junctional adhesion or cell-ECM focal adhesion. In the model, a single **junctional adhesion** between RPE cells and BrM represents the complex biological adhesion between RPE cells and their basal laminae (RBaL), adhesion between the basal laminae and their basement membrane (RBaM) and adhesion between RBaM and BrM (inset). Modeled adhesion processes are: **EC-EC** and **EC-BrM junctional adhesion**; **EC-RPE**, **EC-POS** and **EC-PIS labile adhesion**; **RPE-RPE** and **RPE-BrM junctional adhesion**; **RPE-PIS** and **RPE-POS labile adhesion**; **PIS-PIS**, **PIS-POS** and **POS-POS junctional adhesion**. Key: **BrM**: Bruch's membrane, **RPE**: retinal pigment epithelium, **RBaM**: basement membrane of the RPE, **RBaL**: basal lamina of the RPE, **POS**: photoreceptor outer segment, **PIS**: photoreceptor inner segment.

Our model represents the in-plane epithelial junctions between healthy RPE cells by **junctional adhesion** with both strong **labile adhesion** and strong **plastic coupling** between **RPE cells** (Figure 2). Since RPE cells adhere strongly to their basement membranes, we treat the RBaM as a part of each **RPE cell** and assume that attachment of RPE cells to BrM depends on the adhesion of the RBaM (not of basal lamina) to BrM. During RPE detachment, cleavage occurs between the RBaM and BrM. Since we do not model the RBaM, we represent this RPE-RBaM-BrM adhesion as a single **junctional adhesion** between **RPE** and **BrM cells** (**RPE-BrM junctional adhesion**) (Figure 2). Since no known junctional structures couple RPE to the POS, we represent RPE-POS adhesion by relatively weak **RPE-POS labile adhesion** between **RPE cells** and **POS cells**. **RPE-POS labile adhesion** is weak relative to the strength of **labile adhesion** in **RPE-RPE junctional adhesion**. Neighboring **PIS** and **POS cell segments** adhere via **junctional adhesion** (Figure 2), limiting traverse **photoreceptor** displacement under normal conditions.

In an endothelium, ECs mainly adhere to other ECs via *vascular endothelial cadherin* (*VE-Cadherins*) and tight junctions. When forming the **CC**, modeled **ECs** adhere via **junctional adhesion** to neighboring **ECs** and **BrM** (Figure 2). **Stalk cells** (representing activated ECs) adhere to all other **ECs** via **junctional adhesion** with **labile adhesion** at the same strength as **ECs** in the **CC**, but have relatively weak **plastic coupling** to **ECs** and **BrM** (Figure 2). We assume **ECs** adhere to the **RPE** and to photoreceptor segments (**PIS** and **POS** generalized **cells**) weakly via **labile adhesion** representing nonspecific biological surface adhesion (Figure 2).

Adherent cells suspended in liquid assume a spherical shape, meaning that non-specific cell-liquid adhesion is weak. We represent this weak cell-liquid adhesion by weak **labile adhesion** between **cells** and **medium**. Thus **cells** prefer to adhere to other **cells** or **BrM** rather than to be surrounded by **medium**.

We do not model explicitly the differences in adhesion between the apical, lateral, and basal surfaces of biological RPE cells and photoreceptors. In our model **labile adhesion** strengths depend only on the types of **cells** or **membranes** in contact, not on the apical, basal or lateral identity of the contacting regions. However, effective cell polarization in the model develops from the specific geometry and contacts which occur in the normal and diseased retina, so at most times, **cells** emergently exhibit correctly polarized adhesivity, even though we do not impose it. We also neglect temporal adhesion changes observed clinically or experimentally during CNV progression, assuming temporally constant adhesivity of **RPE**, **POS**, **PIS** **cells** in our individual simulations.

Since we can vary independently the strength of the **labile adhesion** and **plastic coupling** which contribute to junctional adhesion, we refer to the labile components of **RPE-RPE** or **RPE-BrM junctional adhesion** as **RPE-RPE** or **RPE-BrM labile adhesion** and to the **plastic coupling** components of **RPE-RPE** or **RPE-BrM junctional adhesion** as **RPE-RPE** or **RPE-BrM plastic coupling**.

#### Angiogenic and antiangiogenic factors

To aggregate the effects of RPE-derived diffusible growth factors on the **choriocapillaris** and **CNV** capillaries we include a diffusible growth-factor **field, RPE-derived VEGF-A** which represents the aggregate proangiogenic effect of all biological long-diffusing proangiogenic and antiangiogenic factors. All types of **ECs** in our model take up **RPE-derived VEGF-A** uniformly at a constant rate. We omit growth factors and cytokines from other sources. Since we assume that PEDF affects CNV only as an anti-angiogenic factor and that it diffuses at the same rate as RPE-derived VEGF, we can combine the effects of the two RPE-derived VEGF isoforms (120 and 165) and PEDF on CNV growth and regression into an effective cell response to **RPE-derived VEGF-A**. While numerous other diffusible proangiogenic and antiangiogenic factors [63], [64] may play a modulatory role in capillary behavior (see *Inflammation* subsection in supplementary Text S1), we lack detailed experimental data on their spatial and temporal distribution and function. All modeled **ECs** (including **CC** and activated **ECs**, see below) secrete a **short-diffusing VEGF-A**. The **short-diffusing VEGF-A** is not a survival factor for **ECs** and we ignore its uptake by **ECs**. Both **short-diffusing VEGF-A** and **RPE-derived VEGF-A** decay at constant rates and diffuse uniformly everywhere in our modeled **retina**. However, the two **VEGF-A** *diffusion lengths* differ significantly. Typically, **RPE-derived VEGF-A** diffuses ∼5 times farther than **short-diffusing VEGF-A**. Assuming the decay rates for both **RPE-derived VEGF-A** and **short-diffusing VEGF-A** are the same, a five-fold difference in diffusion length translates into a twenty-five fold larger diffusion constant for **RPE-derived VEGF-A** compared to **short-diffusing VEGF-A**.

#### Angiogenesis and BrM degradation

Computer simulations can help us analyze the role of multiple mechanisms during angiogenesis, both in pathological conditions like tumor-induced angiogenesis [65]–[68] and corneal angiogenesis [69] and in healthy tissues like muscle [70]. Here, we use a multi-cell 3D angiogenesis model which we have previously described [65] to simulate **CNV** growth and patterning. Our model includes two types of activated **ECs**: **tip cell** and **stalk cell types**. The **tip cell type** is a transient **cell type** that lasts one day, then differentiates into the **stalk cell type**. **Cells** of **stalk cell type** remain **stalk cells**. Both **stalk** and **tip cells** migrate via chemotaxis up gradients of both **RPE-derived VEGF-A** and **short-diffusing VEGF-A** at any **cell** boundaries which are not in contact with other **ECs**, *i.e.* they exhibit contact-inhibited chemotaxis. Thus, a **stalk cell** at the tip of an angiogenic sprout, which has less contact area with other **ECs**, migrates more strongly than other **stalk cells** in response to **VEGF-A** gradients. Thus **stalk cells** can function as biological endothelial tip cells, leading other **stalk cells** in the trunk of an angiogenic sprout, as seen in experiments. **Stalk cells** self-organize into capillary-like network patterns [71].

Since experiments suggest that both ECs and macrophages can function as tip cells in CNV, we can interpret a **tip cell** either as an EC tip cell or as an immune cell that invades BrM. Modeled **tip cells** secrete a single **MMP field**. Our model is agnostic about the type of MMP, though experiments seem to favor MMP-2 which is activated on contact with tip cells or macrophages expressing MT1-MMP. Our model's **MMP field** represents a very short-diffusing molecule that degrades **BrM**. If we assume that other types of MMP remain bound to the EC, it does not greatly affect our simulations, data not shown. Active tip cells produce MMPs at rates which allow them to invade even dense ECM at speeds of more than a few µm per day (*e.g*. during intersegmental blood vessel formation in zebrafish embryos). At this rate of degradation, a single **tip cell** could destroy the entire simulated **BrM** within a few simulated months. However, the typical hole size in BrM *in vivo* is less than a few cell diameters [29]. To generate a simulated hole in **BrM** compatible with these *in vivo* observations (a roughly one cell-diameter hole), we must switch off **MMP** secretion by the **tip cell** after 24 simulated hours. In our simulations we represent this cessation of MMP secretion by the differentiation of the **tip cell** into a **stalk cell** at 24 h. We neglect the slow reconstruction of BrM by RPE cells.

#### Inflammation

We represent the adhesion-reducing effects of inflammation due to inflammatory factors and immune cells implicitly by weakening **RPE-RPE**, **RPE-POS** (due to acute inflammation) and **RPE-BrM adhesion** (due to chronic irregularities in complement cascade). We neglect the role of inflammation on juxtacrine Delta/Notch coupling between ECs.

#### Cell proliferation and death

Typically, adherent cells like RPEs need to adhere to other cells (of the same or different types) or to an appropriate substrate to remain viable. Otherwise they die. Modeled **RPE cells** require **RPE-RPE** and **RPE-BrM** contact to remain viable. In the absence of such contact, **RPE cells** die. **RPE cells** do not proliferate or grow. Both **CC** and **ECs** require a low concentration of **RPE-derived VEGF** to remain viable, and die below a threshold level of **RPE-derived VEGF-A**. **Stalk cells** grow at a rate depending on the local concentration of **RPE-derived VEGF-A** unless their growth is inhibited by **stalk-EC** contact (contact-inhibited growth, see the *Methods* section in supplementary Text S3 for details).

## Results

In this section, we discuss how adhesion in the **BrM-RPE-POS** complex forms an effective physical barrier to CNV and how adhesion failures increase the risk of CNV initiation. We then relate the different modes of adhesion failure to the resulting **CNV** loci (CNV types), **CNV** progression and translocation (changes of CNV locus). We also discuss the **CNV** dynamics for individual adhesion scenarios and individual simulations representative of those adhesion scenarios.

All simulations begin with either no **tip cell** or one **tip cell**. In our simulations, the **tip cell** degrades **BrM** via **MMP** secretion, forming a roughly one cell diameter hole in **BrM** which allows it to cross **BrM** into the **retina**. The **tip cell** does not divide; 24 **hours** after the start of the simulation, it differentiates into a **stalk cell**, ending its degradation of **BrM**. All other **stalk cells** descend from this **stalk cell**. **Stalk cells** at the tip of angiogenic sprouts behave like biological tip cells. This tip- cell-like behavior allows **stalk cells** to migrate away from existing **stalk-cell** clusters, releasing their contact-inhibition. They then grow and divide when they reach a preset doubling-volume. **CNV** refers to the ensemble of **stalk cells** in a simulation. We define the time of **CNV onset** (the ***CNV*** *initiation time*) to be the time at which the total number of **stalk cells** exceeds three. For implementation parameter values and additional simulation details see the *Methods* section in supplementary Text S3.

We simulated 108 different adhesion *scenarios* (sets of adhesion parameters) (ID: 1 to 108) assigning one of three levels: *normal*: 3, *moderately impaired*: 2 and *severely impaired* (*weak*): 1 to each of the five key adhesion parameters: 1) the **RPE-RPE labile adhesion** strength (*RRl*), 2) the **RPE-RPE plastic coupling** strength (*RRp*), 3) the **RPE-BrM labile adhesion** strength (*RBl*), 4) the **RPE-BrM plastic coupling** strength (*RBp*), and 5) the **RPE-POS labile adhesion** strength (*ROl*). For each adhesion scenario we simulated our **retina** both in the absence and presence of a **tip cell** for one simulated **year**. In the presence of a **tip cell**, **CNV** may initiate (see below, in the *Necessary and Sufficient Conditions for CNV Initiation* section). We ran 10 simulation replicas for each adhesion scenario in which we included a **tip cell** (1080 simulations), and 3 simulation replicas for each adhesion scenario in the absence of a **tip cell** (324 simulations). Although not all the adhesion scenarios are physiologically likely (for example, Table S7: adhesion scenario ID: 90), a comprehensive exploration of the adhesion-parameter space clarifies the role of each corresponding mechanism in **CNV** initiation and progression.

The results of our simulations are 3D time-varying **structures** and **fields**. Quantitative comparison and classification of patterns and changes in patterns in 3D are often challenging. We have developed a morphometric quantification and classification algorithm (see the *Quantification and Classification of Simulations* subsection in supplementary Text S3 for details) that is able to classify CNV patterns, *i.e.* CNV types, and their time-dependent (CNV-type) changes, *i.e.* CNV progression (Table 3, Table 4, Table 5). Our algorithm calculates a *morphometric weight*, *MW*, based on the total contact area between **stalk cells** and **BrM**, and between **stalk cells** and the **POS**. A *MW* close to 1 indicates that most **stalk cells** are confined between the **RPE** and **BrM** (**sub-RPE**) in **Type 1 CNV**. A *MW* close to 0 indicates that most **stalk cells** are confined between the **RPE** and **POS** (**sub-retinal**) in **Type 2 CNV**. A ***MW*** close to 0.5 usually indicates **Type 3 CNV** (see the *Quantification and Classification of Simulations* subsection in supplementary Text S3 for additional conditions when *MW*∼0.5). Our computational classification is compatible with current static histological classifications and can be applied to images of appropriately labeled histological sections and 3D microscopy images of the retina.

**Table 3:** Classification of CNV type based on Morphometric Weight.

Morphometric Weights (*MW*s)	CNV Type
*MW*≥0.75	**Type 1**
*MW*≤0.25	**Type 2**
0.25<*MW*<0.75	**Type 3**

We define the type of **CNV** based on the mean morphometric weight during a three **month** window. A *MW*≥0.75 throughout the window indicates that most **stalk cells** lie between **BrM** and the **RPE** (in the **sub-RPE** space) and do not contact the **POS**. We therefore assign the time window to **Type 1**. A *MW*≤0.25 throughout the window indicates that most **stalk cells** lie between **RPE** and the **POS** (in **sub-retinal** space) and do not contact **BrM**. We therefore assign the time window to **Type 2 CNV**. 0.25<*MW*<0.75 usually indicates that **stalk cells** occur in both the sub-**RPE** and **sub**-**retinal** spaces. In a few exceptional cases (Table S10) with 0.25<*MW*<0.75, most **stalk cells** lie between neighboring **RPE cells** rather than in either the **sub**-**RPE** or **sub**-**retinal** spaces (discussed in the *Stable Type 3 CNV* subsection in supplementary Text S5).

**Table 4:** (Temporal) Nomenclature for CNV.

CNV Classification	Relevant Adhesion Scenarios
**Early Type 1** (**ET1**)	Table S2
**Late Type 1** (**LT1**)	-
**Early Type 2** (**ET2**)	Table S3
**Late Type 2** (**LT2**)	-
**Early Type 3** (**ET3**)	Table S4
**Late Type 3** (**LT3**)	-

To classify **CNV** progression dynamics during a simulated **year**, we determine the ***early*** and ***late*** loci of **stalk cells** using the mean *MW*s during the first and last three **months** of each simulation (we can make this calculation whether or not **CNV** initiates).

**Table 5:** Nomenclature for CNV dynamics.

Dynamics Classification	CNV Dynamics	Relevant Adhesion Scenarios
Stable Type 1 (***S11***)	**Early Type 1→Late Type 1**	Table S5
Sub-RPE to Sub-Retinal Translocation (***T12***)	**Early Type 1→Late Type 2**	Table S6
Sub-RPE to Sub-Retinal Progression (***P13***)	**Early Type 1→Late Type 3**	Table S7
Sub-Retinal to Sub-RPE Translocation (***T21***)	**Early Type 2→Late Type 1**	Not Observed
Stable Type 2 (***S22***)	**Early Type 2→Late Type 2**	Table S8
Sub-Retinal to Sub-RPE Progression (***P23***)	**Early Type 2→Late Type 3**	Table S9
Type 3 to Sub-RPE Translocation (***T31***)	**Early Type 3→Late Type 1**	Not Observed
Type 3 to Sub-Retinal Translocation (***T32***)	**Early Type 3→Late Type 2**	Not Observed
Stable Type 3 (***S33***)	**Early Type 3→Late Type 3**	Table S10

We classify **CNV** progression dynamics in each simulation based on its **early** and **late** **CNV types**, allowing for nine **CNV**-dynamics scenarios. We use the term *progression* when a simulation replica initially develops either **Early Type 1 CNV** or **Early Type 2 CNV** and then develops **Late Type 3 CNV** and *translocation* when a replica initially develops either **Early Type 1 CNV** or **Early Type 3 CNV**, then **Late Type 2 CNV** or initially develops either **Early Type 2 CNV** or **Early Type 3 CNV**, then **Late Type 1 CNV**. Three translocation scenarios, **T21**, **T31** and **T32**, did not occur in our simulations.

**Figure 3:**
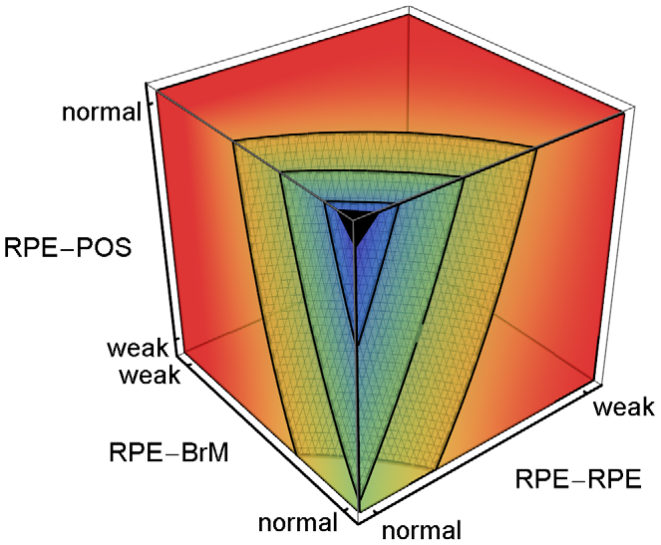
CNV Initiation probability dependence on key adhesion mechanisms. 3D plot of the regression-inferred **CNV** initiation probability (*P*_init_) vs. three key adhesion strengths using ten simulation replicas for each adhesion scenario in the 3D parameter space obtained by setting *RRp* = *RRl* and *RBp* = *RBl*. Red corresponds to *P*_init_ = 1 and purple to *P*_init_ = 0. The black region at the top-front corner indicates the locus of normal adhesion. The three isosurfaces of **CNV** initiation probability correspond to *P*_init_ = 0.25 (front), 0.5 (middle) and 0.75 (back). The five adhesion parameters and their (multi)linear combinations account for 88% of the observed variance in **CNV** initiation probability (adjusted *R^2^* = 0.83). Regression predicts a minimum **CNV** initiation probability of 0.08 for normal adhesion, much higher than observed in either our simulations or experiments. For normal **RPE-POS labile adhesion**, moderate impairment of either **RPE-RPE** (*RRp* = *RRl*) or **RPE-BrM** (*RBp* = *RBl*) **junctional adhesion** increases the **CNV** initiation probability to ∼50%. Severe impairment of **RPE-POS** increases the **CNV** initiation probability to ∼50% even when both **RPE-RPE** and **RPE-BrM** are normal.

We use multiple-regression analysis to relate the **CNV** initiation probability, types, progression and dynamics to the typical adhesion scenarios which cause them (*e.g.*, Figures 3–6). When statistical inference is ambiguous (*R* ^2^<0.7), we look at individual simulation time series in detail.

**Figure 4 pcbi-1002440-g004:**
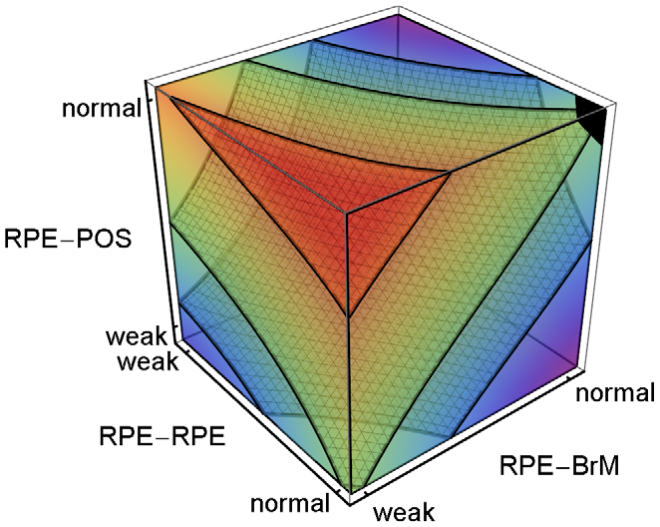
Sub-RPE CNV dependence on adhesion. 3D plot of the regression-inferred average *MW* using 10 simulation replicas for each adhesion scenario in the 3D parameter space obtained by setting *RRp* = *RRl* and *RBp* = *RBl*. The average *MW* shows the **stalk cell** locus even when **CNV** fails to initiate, so a region *prone* to **ET1 CNV** develops **ET1 CNV** only if **CNV** initiates. Red corresponds to *MW* = 1 and purple corresponds to *MW* = 0. The black region at the top-left corner indicates the locus of normal adhesion. *MW* = 1.0 for **RPE-RPE junctional adhesion** normal, **RPE-BrM junctional adhesion** severely impaired (weak) and **RPE-POS labile adhesion** normal. The three isosurfaces correspond to *MW* = 0.25 (back), 0.5 (middle) and 0.90 (front). The five adhesion parameters and their (multi)linear combinations account for 93% of the observed variance in average *MW* for all 108 adhesion scenarios (adjusted *R* ^2^ = 0.89). Severe impairment of **RPE-POS labile adhesion** greatly reduces the *MW*, so **ET1 CNV** can only occur when **RPE-POS labile adhesion** is near normal. Scenarios with severe impairment of **RPE-BrM junctional adhesion** (*RBp* = *RBl* = 1), and normal **RPE-POS labile adhesion** are prone to **ET1 CNV** for a wide range of **RPE-RPE junctional adhesion** impairment (*MW*>0.95 for *RRp* = *RRl*>1.5). The red region with *MW*>0.9 has *P*_init_>0.8. We have rotated the axes from their orientation in Figure 3 to show the regions in the parameter space prone to **ET1 CNV**. To show the structure of the isosurfaces, we have rotated the axes relative to Figure 3.

**Figure 5 pcbi-1002440-g005:**
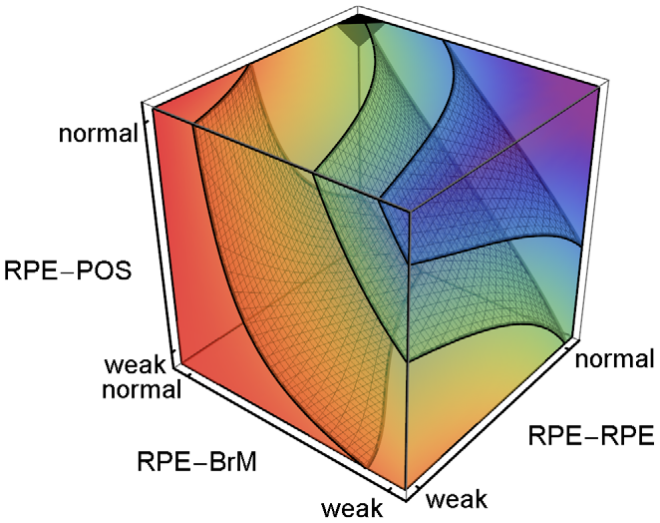
Sub-Retinal CNV dependence on adhesion. 3D plot of the regression-inferred average (1−*MW*) using 10 simulation replicas for each adhesion scenario in the 3D parameter space obtained by setting *RRp* = *RRl* and *RBp* = *RBl*. The average (1−*MW*) shows the **stalk cell** locus even when **CNV** fails to initiate, so a region *prone* to **ET2 CNV**, develops **ET2 CNV** only if **CNV** initiates. Red corresponds to (1−*MW*) = 1 and purple corresponds to (1−*MW*) = 0. The black region at the top-back corner indicates the locus of normal adhesion. The three isosurfaces correspond to (1−*MW*) = 0.25 (right), 0.5 (middle) and 0.90 (left). The five adhesion parameters and their (multi)linear combinations account for 93% of the observed variance in average *MW* for all 108 adhesion scenarios (*R* ^2^ = 0.89). The red region with (1−*MW*)>0.9, can be divided into three sub-regions: 1) When **RPE-RPE junctional adhesion** is normal, **RPE-BrM junctional adhesion** is moderately impaired, and **RPE-POS labile adhesion** is severely impaired (weak). 2) When **RPE-RPE junctional adhesion** is severely impaired (weak) and **RPE-BrM junctional adhesion** is normal, independent of **RPE-POS labile adhesion**. 3) When **RPE-RPE** adhesion is weak, **RPE-BrM** adhesion is moderately to severely impaired, and **RPE-POS** adhesion is severely impaired. The red region does not include all adhesion scenarios in Table S3 leading to **Early Type 2 CNV**. To show the structure of the isosurfaces, we have rotated the axes relative to Figure 3.

**Figure 6:**
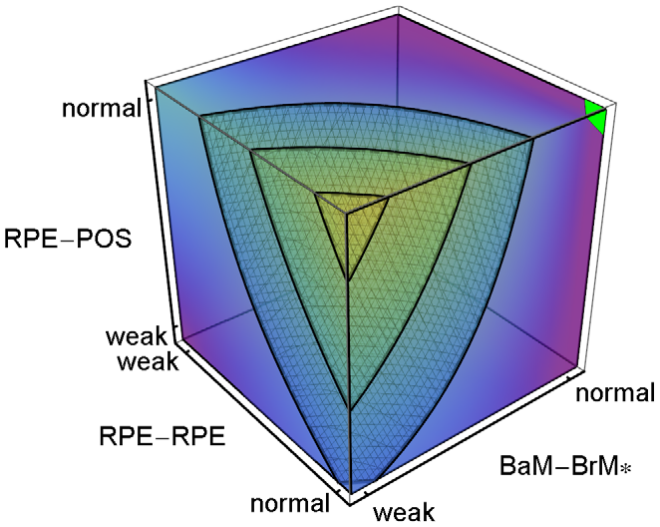
Stable Type 1 CNV dependence on adhesion. 3D plot of the regression-inferred probability of occurrence of **Stable Type 1 CNV** (**S11 CNV** probability) using 10 simulation replicas for each adhesion scenario in the *asymmetrically* reduced parameter space obtained by setting *RRp* = *RRl* and *RBp* = 3 (indicated by the RPE-BrM* axis label). Red corresponds to a **S11 CNV** probability of 1 and purple corresponds to a **S11 CNV** probability of 0. The black region at the top-left corner indicates the locus of normal adhesion. The maximal regression-inferred probability of **S11 CNV** is 0.93 when **RPE-RPE junctional adhesion** is normal (*RRp* = *RRl*), **RPE-BrM labile adhesion** is severely impaired (*RBl* = 1), **RPE-BrM plastic coupling** is normal (*RBp* = 3), and **RPE-POS labile adhesion** is normal. The three isosurfaces correspond to **S11 CNV** probabilities of 0.25 (back), 0.5 (middle) and 0.8 (front). The five parameters and their (multi)linear combinations account for 76% of the observed variance in the probability of occurrence of **S11 CNV** (*R* ^2^ = 0.67). Severe impairment of **RPE-POS labile adhesion** and **RPE-RPE junctional adhesion** greatly reduces *MW*, so **S11 CNV** can only occur when both adhesion strengths are near normal. To show the structure of the isosurfaces, we have rotated the axes relative to Figure 3.

### Necessary and Sufficient Conditions for CNV Initiation

**CNV** initiation in our simulations requires: 1) a **tip cell**, and 2) adhesion failures. A **tip cell** is not sufficient to initiate **CNV** if all adhesions are normal. Even when a **tip cell** makes a hole in **BrM**, crosses **BrM** and differentiates into a **stalk cell CNV** does not initiate if all adhesions are normal (Table S1, adhesion scenario ID:1).

The strong adhesion of **RPE cells** to neighboring **RPE cells**, **POSs** and **BrM** means that the **BrM-RPE-POS** ensemble behaves as a mechanically stable *complex*. This complex effectively obstructs CNV by limiting the proliferation and invasion of **stalk cells** into the **sub-RPE** and **sub-retinal** spaces. We discuss, in greater detail below, how different modes of adhesion failure in the **complex** allow **stalk cells** to proliferate and invade. Chemotaxis greatly affects how **stalk cells** invade the **sub-RPE** and **sub-retinal** spaces. **Stalk cells** migrate via chemotaxis up gradients of both **short-diffusing** and **RPE-derived VEGF-A**. **RPE-derived VEGF-A** is especially important because its concentration is maximal in the **RPE**, encouraging **stalk cells** to migrate from the **CC** into the **sub-RPE** and **sub-retinal** spaces. Adhesion between components of the **BrM-RPE-POS** complex opposes such invasion, inhibiting **CNV** initiation. Our simulations show that finite-strength adhesive interactions among the components of the **BrM-RPE-POS** complex can prevent invasion by **stalk cells** if the adhesion forces are greater than the forces which **stalk cells** exert on the **RPE-RPE** and **RPE-BrM** boundaries due to chemotaxis. Because of the complicated interactions among components during angiogenesis, the existence of such adhesion thresholds is not obvious *a priori*. Our simulations therefore allow us to refine our understanding of these thresholds to show that EC-EC adhesion is also important in vivo. Self-organization of **ECs** into a capillary network pattern requires: 1) Strong chemotaxis forces that balance **EC-EC** adhesion (**ECs** form clusters rather than networks when chemotaxis to both **short-diffusing** and **RPE-derived VEGF-A** is weak), 2) the **EC-EC** adhesion strength must be comparable to the adhesion strengths in the **BrM-RPE-POS** complex. Thus the strength of adhesion among **RPE-RPE**, **RPE-BrM** and **RPE-POS** required to resist chemotaxing **ECs** also depends on **EC-EC** adhesivity.

We performed multiple-regression analysis (see the *Methods* section in supplementary Text S3 for details) against five adhesion parameters (*RRl*, *RRp*, *RBl*, *RBp*, *ROl*) to relate specific adhesion failures to the probability of **CNV** initiation. The five adhesion parameters and their (multi)linear combinations account for 88% of the observed variance in the **CNV** initiation probability (adjusted *R^2^* = 0.83). To visualize the five-dimensional (*5D*) regression relations, we reduce 5D to 3D by assuming that *RRp* = *RRl* and *RBp* = *RBl*. We call this reduction *symmetric* since it assumes that impairing **RPE-RPE labile adhesion** also impairs **RPE-RPE plastic coupling**. *E.g.*, we would expect changes in cytoskeletal architecture due to inflammation to affect both adhesion mechanisms together. We consider *asymmetric reductions* in adhesion later in this section. Figure 3 shows a 3D volumetric visualization of the **CNV** initiation probability (*P*_init_) in the symmetrically reduced parameter space (*RRp* = *RRl* and *RBp* = *RBl*). The three isosurfaces of **CNV** initiation probability correspond to *P*_init_ = 0.25, 0.5 and 0.75 in order from front to back. Regression predicts a minimum **CNV** initiation probability of 0.08 for normal adhesion, while **CNV** did not initiate in any of our simulation replicas for normal adhesion. Thus the simulated **CNV** initiation probability for normal adhesion is effectively 0, as observed clinically, while the regression-inferred 0.08 initiation probability is an artifact of the linear inference, an example of the greater predictive power of mechanistic simulations compared to pure statistical inference.

Figure 3 shows that when **RPE-POS labile adhesion** is normal, even moderate impairment of either **RPE-RPE** (*RRp* = *RRl*) or **RPE-BrM** (*RBp* = *RBl*) **junctional adhesion** increases the **CNV** initiation probability to ∼50%. When both **RPE-RPE** and **RPE-BrM junctional adhesion** are normal (*RRp* = *RRl* = *RBp* = *RBl* = 3) and **RPE-POS labile adhesion** is severely impaired (*ROl* = 1) **CNV** initiation probability is also ∼50%. Thus, for severe impairments of any one of the three adhesion failure mechanisms can each independently induce **CNV**, as predicted both by our regression model and our mechanistic interpretation. Adhesion failures in the **BrM-RPE-POS** complex show strong combinatorial effects. When either **RPE-RPE** or **RPE-BrM junctional adhesion** is moderately impaired and **RPE-POS labile adhesion** is severely impaired, **CNV** initiation probability increases to 100%. Table S1 shows that asymmetrical impairment of either **RPE-RPE** or **RPE-BrM plastic coupling** alone, without impairment of the corresponding **labile adhesion** barely increases the probability of **CNV** initiation. Thus, the **plastic coupling** strengths have only a minor effect on the ability of the **BrM-RPE-POS** complex to oppose **CNV**.

We believe that our simulated initiation probabilities are higher than those observed in experiments due to two simplifying assumptions of our model: 1) We assumed that all **stalk cells** can divide indefinitely. Limiting the number of **stalk-cell** divisions (due to senescence), would lower the probability of CNV initiation. In *in vitro* experiments, less than 2% of endothelial cells have high proliferation potential [72], [73], so angiogenic sprouts often fail to grow, or even regress, if the ECs forming the sprouts have low proliferation potential. 2) We assumed that **stalk cells** adhere equally to both sides of **BrM**, while BrM and CC basement membrane are histologically distinct. In experiments, ECs haptotax to the basement membrane of the CC on the outer side of BrM (Figure 1), reducing their probability of crossing BrM compared to our simulations. We expect that increasing adhesion between **stalk cells** and the **CC**-side of **BrM** would reduce the **CNV** initiation probability, since **stalk cells** would then prefer to remain on the **CC**-side of **BrM**, where contact with the preexisting vasculature would inhibit their growth.

### Early and Late CNV and CNV Progression

To classify **CNV** progression during a simulated **year**, we determine the ***early*** and ***late*** loci of **stalk cells**, using the mean (weighted by the number of **stalk cells**) *MW*s during the first and last three **months** of each simulation whether or not **CNV** initiates (we can calculate the *MW* even if CNV fails to spread and thus in the absence of initiation). The mean *MW* measures the ability of the **BrM-RPE-POS** complex to confine **stalk cells** to specific regions. A *MW*≥0.75 during a given time interval indicates that most **stalk cells** lie between **BrM** and the **RPE** (in the **sub-RPE** space) and that they do not contact the **POS**. We therefore assign the time interval to **Type 1 CNV**. A *MW*≤0.25 during a given time interval indicates that most **stalk cells** lie between the **RPE** and the **POS** (in **sub-retinal** space) and do not contact **BrM**. We therefore assign the time interval to **Type 2 CNV**. 0.25<*MW*<0.75 usually indicates that **stalk cells** occur in both the **sub-RPE** and **sub-retinal** spaces (since few **sub-RPE stalk cells** touch the **POS** and few **sub-retinal** **stalk cells** touch **BrM**). We therefore assign the time interval to **Type 3 CNV**. In a few exceptional cases (Table S10) with 0.25<*MW*<0.75, most **stalk** **cells** lie between neighboring **RPE cells** rather than in either the **sub-RPE** or **sub-retinal** spaces (discussed in the *Stable Type 3 CNV* section in supplementary Text S5). The type of **CNV** during the early window which we call the **early CNV type** (the first three months of a simulation) is especially revealing, because all simulations start from the same initial condition. The **early CNV type** shows the efficacy of the **BrM-RPE-POS** complex in blocking **stalk cell** invasion of the **sub-RPE** and **sub-retinal** spaces. Since tissue structure and cell function can change significantly in the **BrM-RPE-POS** complex during a simulated **year**, changes in **CNV type** between the **early** and **late** windows can result from either structural or barrier-function changes in the **BrM-RPE-POS** complex. For example, the adhesion failures which typically lead to **Early Type 3 CNV** differ from those which typically leading to **Late Type 3 CNV**.

Because our simulations are stochastic, different replicas of the same adhesion scenario can lead to different combinations of **early** and **late types** of **CNV**. Such variation is common clinically and indicates that a simple population average of *MW*s over simulation replicas may reveal neither the **types**, progression dynamics nor degree of heterogeneity of outcomes. To retain this dynamic and population information, we classify **CNV** dynamics (progression) in each simulation separately based on its **early** and **late CNV type**, for a total of 9 CNV dynamics scenarios (Table 5). We use the term *progression* when a simulation replica initially develops either **Early Type 1 CNV** or **Early Type 2 CNV** and then develops **Late Type 3 CNV** and *translocation* when a replica initially develops either **Early Type 1 CNV** or **Early Type 3 CNV**, then **Late Type 2 CNV** or initially develops either **Early Type 2 CNV** or **Early Type 3 CNV**, then **Late Type 1 CNV**. Three translocation scenarios **sub-retinal** to **sub-RPE CNV** (**T21 CNV**), **Type 3** to **sub-RPE CNV** (**T31 CNV**) and **Type 3** to **sub-retinal CNV** (**T32 CNV**) did not occur in our simulations.

**Early Type 1 (ET1) CNV and its progression. Stalk cells** remain confined to the **sub-RPE** space in two main classes of adhesion scenarios: 1) When **RPE-BrM labile adhesion** is moderately to severely impaired, **RPE-BrM plastic coupling** satisfies *RBl+RBp*≤4, and both **RPE-RPE** and **RPE-POS labile adhesion** are normal (*RRl* = 3 and *ROl* = 3). 2) When both **RPE-RPE** and **RPE-BrM labile adhesion** are severely impaired (*RRl* = 1 and *RBl* = 1), **RPE-BrM plastic coupling** is moderately to severely impaired (*RBp*≤2), and both **RPE-RPE plastic coupling** and **RPE-POS labile adhesion** are normal (*RRp* = 3, *ROl* = 3) (Table S2, adhesion scenario ID: 83 and 84). In both classes of adhesion scenarios, **CNV** initiation leads to **Early Type 1 CNV** (**ET1**). Table 6 shows the *MW* and **CNV** initiation probabilities for the adhesion scenarios most prone to **ET1 CNV** (*MW*>0.9).

**Table 6:** Adhesion scenario classification based on early CNV type.

		Typical Adhesion Scenario				
Early CNV	Sub-classes	*RRl*	*RRp*	*RBl*	*RBp*	*ROl*
Type 1	1	3	*	1,2	*RBl+RBp*≤4	3
	2	1	3	1	1,2	3
Type 2	1	3	*	2,3	*	1
	2	1	*	2,3	*	*
	3	1	*RBp+RRp*>3	1	*RBp+RRp*>3	1
Type 3	1	1	*	1,2	*	3

Key: *RRl*: **RPE-RPE labile adhesion** strength, *RRp*: **RPE-RPE plastic coupling** strength, *RBl*: **RPE-BrM labile adhesion** strength, *RBp*: **RPE-BrM plastic coupling** strength, *ROl*: **RPE-POS labile adhesion** strength. Scaled adhesion strengths: 3: normal, 2: moderately impaired, 1: severely impaired (weak),

&ast; : all strength levels.

Multiple-regression analysis of the five adhesivities accounted for 93% of the observed variance in the average *MW* for all 108 adhesion scenarios (adjusted *R* ^2^ = 0.89). Figure 4 shows the **stalk cell** locus regression-inferred from the average *MW* as a function of the adhesion parameters obtained by setting *RRp* = *RRl* and *RBp* = *RBl*. Since Figure 4 shows the **stalk cell** locus even when **CNV** fails to initiate, a region *prone* to **ET1 CNV**, develops **ET1 CNV** only if **CNV** initiates. Severe impairment of **RPE-POS labile adhesion** greatly reduces the *MW*, so **ET1 CNV** can only occur when **RPE-POS labile adhesion** is near normal. Scenarios with severe impairment of **RPE-BrM junctional adhesion** (*RBp* = *RBl* = 1), and normal **RPE-POS labile adhesion** are prone to **ET1 CNV** over a wide range of **RPE-RPE junctional adhesion** impairment (*MW*>0.95 for *RRp* = *RRl*>1.5). The red region with *MW*>0.9 has *P*_init_>0.8 (Figure 4).

**Figure 7:**
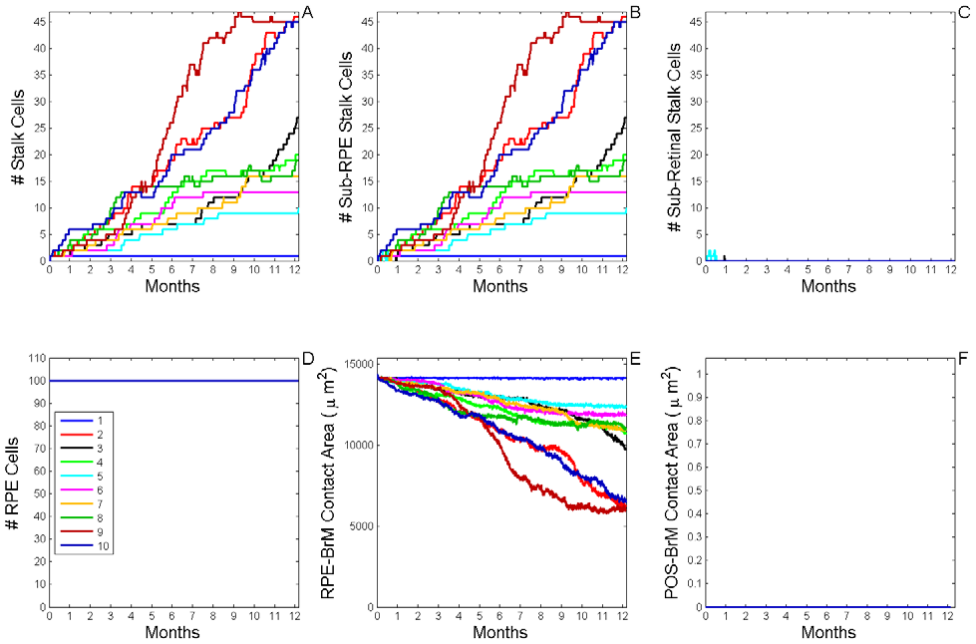
Dynamics of stable Type 1 CNV (S11 CNV). A) Total number of **stalk cells** vs. **time**. B) Total number of **stalk cells** confined in the **sub-RPE** space vs. **time**. C) Total number of **stalk cells** in contact with the **POS** (**stalk cells** in the **sub-retinal** space) vs. **time**. D) Total number of **RPE cells** vs. **time**. E) Total contact area between **RPE cells** and **BrM** vs. **time**. F) Total contact area between **POS cells** and **BrM** vs. **time**. The different colors represent the dynamics of 10 simulation replicas of the adhesion scenario (*RRl* = 3, *RRp* = 3, *RBl* = 2, *RBp* = 2, *ROl* = 3) (Table S5, adhesion scenario ID: 38). (A, B) **CNV** initiates in 9 out of 10 simulation replicas. All develop **Early Type 1 CNV**. **CNV** remains confined in the **sub-RPE** space during one simulated **year** (**Stable Type 1 CNV**). A Fully developed **sub-RPE** capillary network contains about 45 **stalk cells** (∼3000 **cells**/mm^2^). In 5 simulation replicas a few **stalk cells** die during the simulated **year** due to lack of **RPE-derived VEGF-A**. (C) **Stalk cells** have minimal contact with the **POS**. (D, E) The **RPE** remains viable and its total contact area with **BrM** decreases as **stalk cells** proliferate. (F) The **POS** never contacts **BrM**, indicating that the **RPE** does not develop any holes.

**Figure 8:**
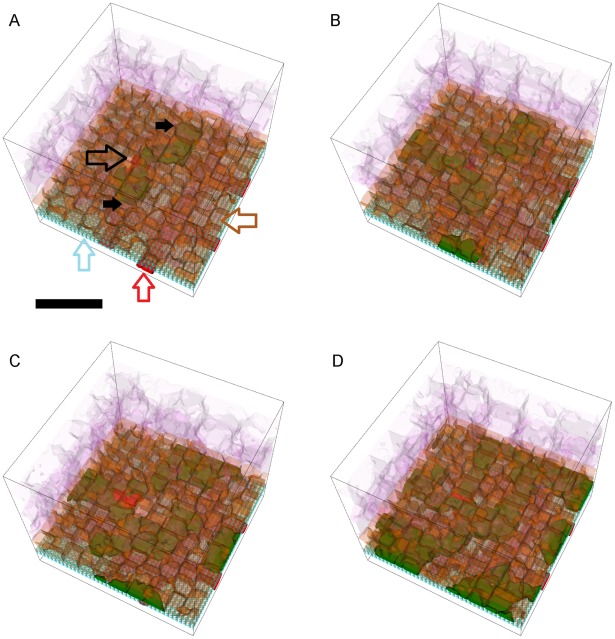
Snapshots of a simulation replica with stable Type 1 CNV. 3D visualization of a simulation replica exhibiting **Stable Type 1 CNV** over one simulated **year** (adhesion scenario ID: 38, simulation ID: 902) (*RRl* = 3, *RRp* = 3, *RBl* = 2, *RBp* = 2, *ROl* = 3). Snapshots of the simulation at **months** 3 (A), 6 (B), 9 (C) and 12 (D). (A) **Stalk cells** (black arrows) invade the **sub-RPE** space through a hole (black outline arrow) in **BrM** (light blue outline arrow) that the **tip cell** opens during the first 24 **hours**. Brown outline arrow shows the **RPE cells**. Red outline arrow shows the **CC** (B, C) **Stalk cells** proliferate until they fill the **sub-RPE** space in **month** 9, after which proliferation slows down (D) The 45 **stalk cells** form a connected capillary network in the **sub-RPE** space. **Cell type** colors: 1) **POS** and **PIS**: light purple, 2) **RPE**: brown, 3) **Stalk cells**: green, 4) **Vascular cells** (**CC**): red, 5) **BrM**: light blue. Scale bar ∼50 µm. We have rendered the boundaries of individual cells as semi-transparent membranes. **POS**, **PIS** and **RPE** cells are more transparent to show the underlying structures. See also Video S1.

In most adhesion scenarios that develop **Early Type 1 CNV** with *MW*>90% the **CNV** remains in the **sub-RPE** space during one simulated **year** (**Late** **Type 1 CNV**, *MW*>75%). *I.e.* they exhibit **Stable T1 CNV** (**S11 CNV**) (see Figure 6, Table 7 and supplementary Text S5 for details). Generally, **CNV** *growth speed* differs from replica to replica in adhesion scenarios prone to **S11 CNV** (compare to **S22 CNV** dynamics, below). Figure 7 shows typical **S11 CNV** dynamics for 10 simulation replicas of a single adhesion scenario (*RRl* = 3, *RRp* = 3, *RBl* = 2, *RBp* = 2, *ROl* = 3) (Table S5, adhesion scenario ID: 38). We visualize snapshots of **S11 CNV** dynamics in one replica in Figure 8 and Video S1.

**Table 7:** Adhesion scenario classification based on CNV dynamics.

		Typical Adhesion Scenarios				
CNV Progression Dynamics	Sub-classes	*RRl*	*RRp*	*RBl*	*RBp*	*ROl*
S11	1	3	2, 3	1, 2	3≤*RBl+RBp*≤4	3
T12	1	3	*RRp+RBp*≥4 except *RRp* = *RBp* = 2	1	*RRp+RBp*≥4 except *RRp* = *RBp* = 2	1
P13	1	1	*	1	1, 2	3
S22	1	3	*	2, 3	*	1
	2	1	*	2, 3	*	*
	3	1	*RBp+RRp*>3	1	*RBp+RRp*>3	1
P23	1	1	1, 2	1	1	1
S33	1	1	*	2	*RBl+RBp*≤4	3
	2	1	*	1	3	3

To simplify, we list only the adhesion scenarios most prone to each type of **CNV** progression dynamics. Key: *RRl*: **RPE-RPE labile adhesion** strength, *RRp*: **RPE-RPE plastic coupling** strength, *RBl*: **RPE-BrM labile adhesion** strength, *RBp*: **RPE-BrM plastic coupling** strength, *ROl*: **RPE-POS labile adhesion** strength. Scaled adhesion strengths: 3: normal, 2: moderately impaired, 1: severely impaired (weak), *: all strength levels. See Table 5, for nomenclature for CNV dynamics.

**Figure 9:**
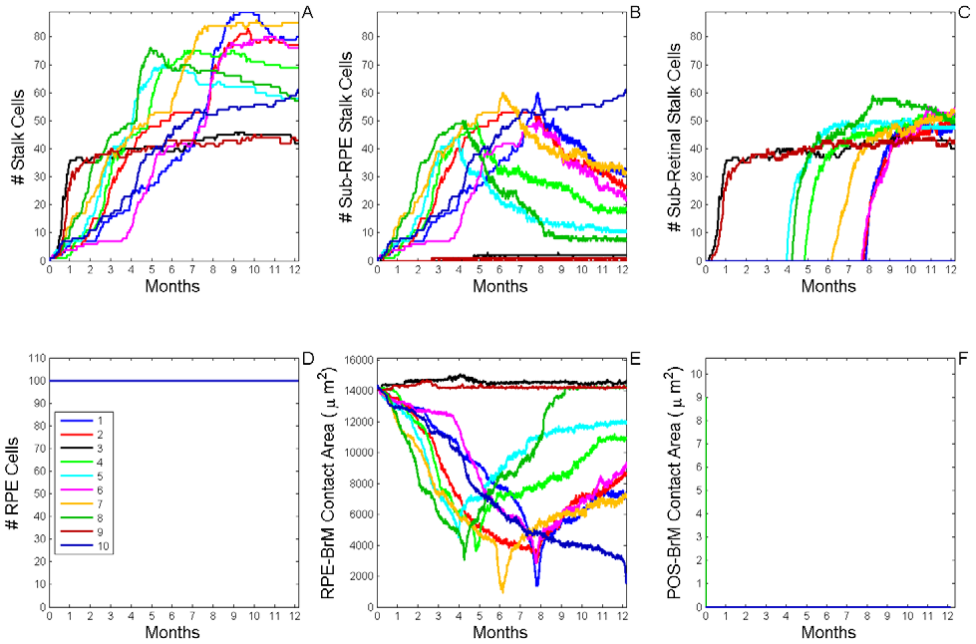
Dynamics of sub-RPE to sub-retinal translocation (T12 Translocation). A) Total number of **stalk cells** vs. **time**. B) Total number of **stalk cells** confined in the **sub-RPE** space vs. **time**. C) Total number of **stalk cells** in contact with the **POS** (**stalk cells** in the **sub-retinal** space) vs. **time**. D) Total number of **RPE cells** vs. **time**. E) Total contact area between **RPE cells** and **BrM** vs. **time**. F) Total contact area between **POS** **cells** and **BrM** vs. **time**. The different colors represent the results of 10 simulation replicas of the adhesion scenario (*RRl* = 3, *RRp* = 3, *RBl* = 1, *RBp* = 1, *ROl* = 1) (Table S6 adhesion scenario ID: 93). (A, B) **CNV** initiates in all replicas. By 3 **months**, most replicas form a developed **sub-RPE** capillary network composed of ∼20 to 40 **stalk cells** (∼1500 to 3000 **cells**/mm^2^). 8 replicas develop **Early Type 1** (**ET1**) **CNV**. Only one replica shows **Stable Type 1** (**S11**) **CNV**. Some **stalk cells** in most replicas die due to lack of **RPE-derived VEGF-A**. (C) Two replicas show **Stable Type 2** (**S22**) **CNV** (**Early** (**ET2**) and **Late Type 2** (**LT2**) **CNV**, black and dark red lines). 7 replicas show **LT2 CNV**. (D) The **RPE** remains viable in all replicas. (E) The contact area between the **RPE** and **BrM** decreases as either **ET1 CNV** or **S11 CNV** develops, and remains constant during **ET2 CNV**. **RPE** reattaches to **BrM** during **T12 CNV**. (F) The **POS** contacts **BrM** once, but the contacts area and duration are both small, so the **RPE** does not develop any persistent or substantial holes.

**Figure 10:**
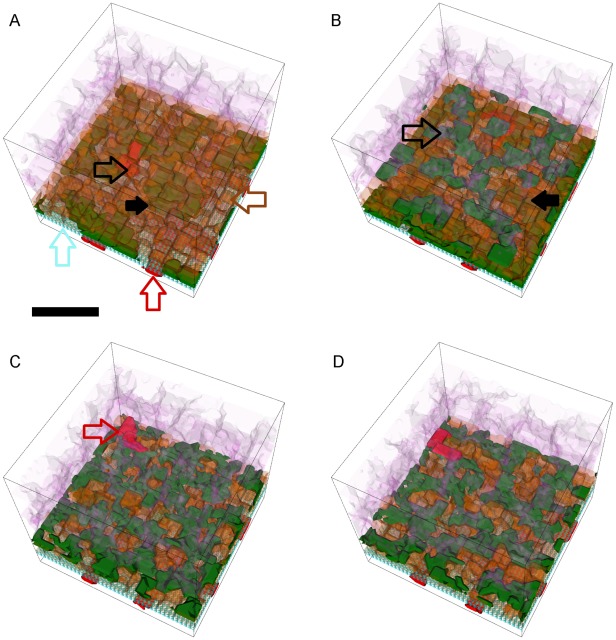
Snapshots of a simulation replica showing sub-RPE to sub-retinal translocation (T12 Translocation). 3D visualization of a simulation replica exhibiting **T12 CNV** translocation during one simulated **year** (*RRl* = 3, *RRp* = 3, *RBl* = 1, *RBp* = 1, *ROl* = 1) (adhesion scenario ID: 93, simulation ID: 849). Snapshots of the simulation at **months** 3 (A), 5 (B), 9 (C) and 12 (D). (A) **Stalk cells** (solid black arrow) invade the **sub-RPE** space through a hole in **BrM** (black outline arrow) and form a capillary network. All **stalk cells** remain in the **sub-RPE** space during the first 3 **months**. A few **vascular cells** fill the hole in **BrM** (black outline arrow) to connect **CNV** capillaries to the **CC** (red outline arrow). Brown outline arrow shows an **RPE cell**. (B) Half of the **stalk cells** (black outline arrow) have crossed the **RPE** and transmigrated into the **sub-retinal** space, forming a new capillary network in the **sub-retinal** space. The black arrow shows a **stalk cell** in the **sub-RPE** space. (C) Most **stalk cells** have transmigrated into the **sub-retinal** space and the **RPE** has completely reattached to **BrM** (Figure 9E, dark green line). A few **vascular cells** of the **CC** have transmigrated into the **sub-retinal** space (red outline arrow) (D) The **sub-retinal** capillary network has fewer **stalk cells** than (C) since **stalk cells** that migrate into the retina far from the **RPE** die. **Cell type** colors: 1) **POS** and **PIS**: light purple, 2) **RPE**: brown, 3) **Stalk cells**: green (**stalk cells** in the **sub-retinal** space have lighter shading), 4) **Vascular cells** (**CC**): red, 5) **BrM**: light blue. Scale bar ∼50 µm. We have rendered the boundaries of individual cells as semi-transparent membranes. **POS**, **PIS** and **RPE** cells are more transparent to show the underlying structures. See also Video S2.

Adhesion scenarios in which some replicas exhibit **T12 CNV** can also have replicas which exhibit either **S22** or **S11** over one simulated **year**. Figure 9 shows **T12 CNV** dynamics for 10 simulation replicas of the adhesion scenario (*RRl* = 3, *RRp* = 3, *RBl* = 1, *RBp* = 1, *ROl* = 1) (Table S6, adhesion scenario ID: 93). We visualize snapshots of the **T12 CNV** dynamics in one replica in Figure 10 and Video S2.

**Figure 11:**
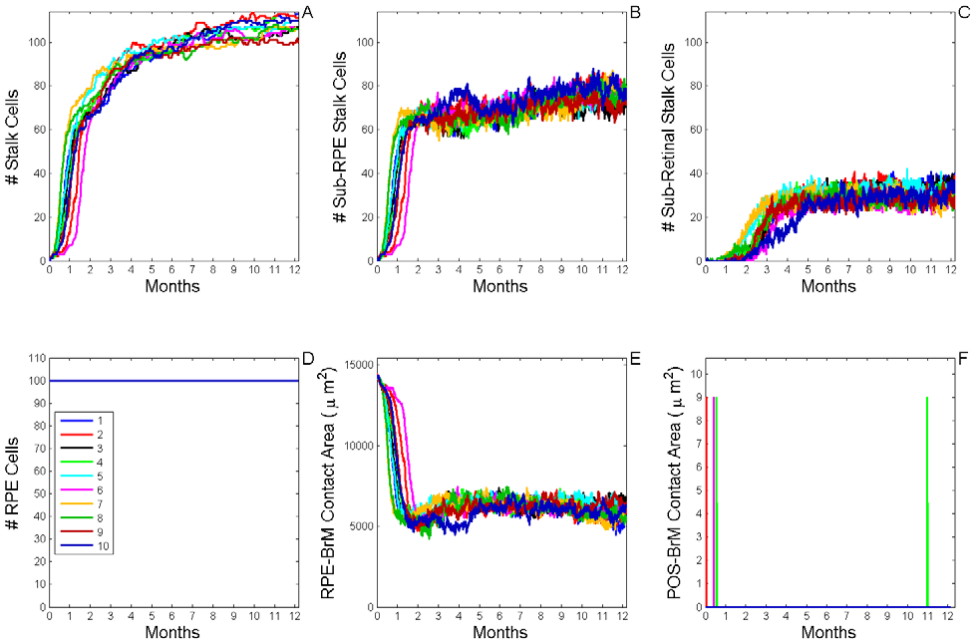
Dynamics of sub-RPE CNV to sub-retinal CNV progression (P13 Progression). A) Total number of **stalk cells** vs. **time**. B) Total number of **stalk cells** confined in the **sub-RPE** space vs. **time**. C) Total number of **stalk cells** in contact with the **POS** (**stalk cells** in the **sub-retinal** space) vs. **time**. D) Total number of **RPE cells** vs. **time**. E) Total contact area between **RPE cells** and **BrM** vs. **time**. F) Total contact area between **POS cells** and **BrM** vs. **time**. The different colors represent the results of 10 simulation replica of the adhesion scenario (*RRl* = 1, *RRp* = 3, *RBl* = 1, *RBp* = 2, *ROl* = 3) (Table S7, adhesion scenario ID: 83). **CNV** initiates in all replicas and all develop **ET1 CNV**. A few **stalk cells** in most replicas die due to lack of **RPE-derived VEGF-A**. (C) **Stalk cells** cross the **RPE** and invade the **sub-retinal** space once the number of stalk cells in the **sub-RPE** space reaches ∼60 **cells**, which usually occurs within first two **months** after initiation. **CNV** progression to the **sub-retinal** space is complete around **month** 5. (D) The **RPE** remains viable in all replicas. (E) The contact area between the **RPE** and **BrM** decreases as **ET1 CNV** develops, and remains constant afterwards throughout **LT3 CNV**. (F) The **POS** contacts **BrM** a few times, but the contact area and duration are both small, so the **RPE** does not develop any persistent or substantial holes.

**Figure 12:**
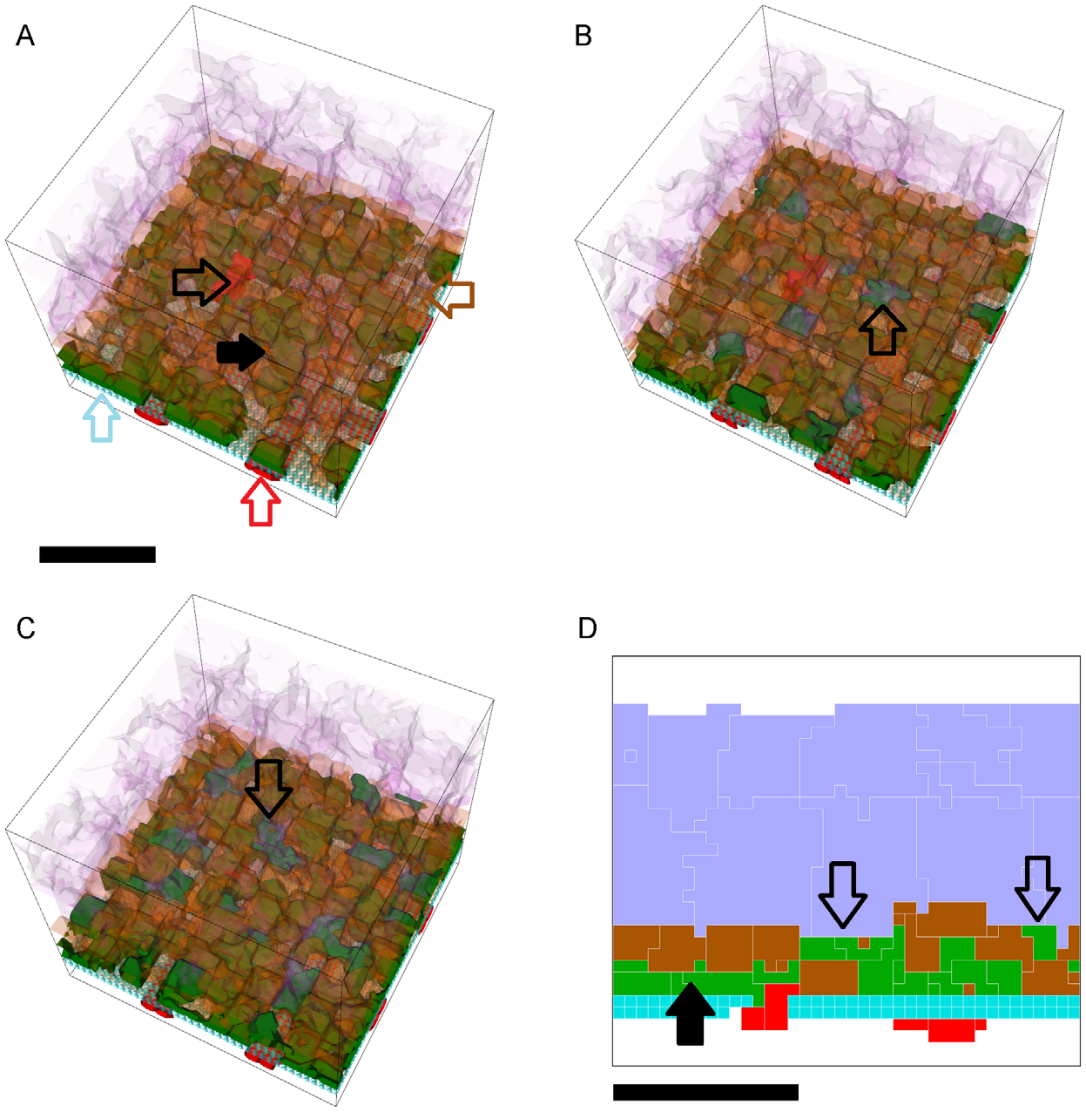
Snapshots of a simulation replica showing sub-RPE CNV to sub-retinal CNV progression (P13 Progression). 3D and 2D visualizations of a simulation replica exhibiting **P13 CNV** progression during one simulated **year** (*RRl* = 1, *RRp* = 3, *RBl* = 1, *RBp* = 2, *ROl* = 3) (Table S7, adhesion scenario ID: 83, simulation ID: 515). Snapshots of the simulation at **months** 1 (A), 2 (B), 6 (C) and 12 (D). (A) **Stalk cells** (solid black arrow) invade the **sub-RPE** space through a hole in **BrM** (blue outline arrow) and form a capillary network. The **vascular cells** (black outline arrow) of the **CC** (red outline arrow) occupy the hole that the **tip cell** forms during the first 24 **hours** of the simulation, connecting the **CNV** capillaries to the **CC**. All **stalk cells** remain in the **sub-RPE** space during the first **month** of the simulation. (B) A few **stalk cells** (black outline arrow) cross the **RPE** into the **sub-retinal** space. (C) Additional **stalk cells** migrate into the **sub-retinal** space and form vascular cords (black outline arrow). (D) A 2D cross-section of the **retina** showing the hole in **BrM**. The **stalk cells** form a **sub-RPE** capillary network (black arrow) connected to a **sub-retinal** capillary network (black outline arrows). Two **vascular cells** connect the **CC** to the **CNV** capillaries through the hole in **BrM**. **Cell type** colors: 1) **POS** and **PIS**: light purple, 2) **RPE**: brown, 3) **Stalk cells**: green (**stalk cells** in the **sub-retinal** space have lighter shading), 4) **Vascular cells** (**CC**): red, 5) **BrM**: light blue. Scale bar ∼50 µm. We have rendered the boundaries of individual cells as semi-transparent membranes. **POS**, **PIS** and **RPE** cells are more transparent to show the underlying structures. See also Video S3.

**CNV** dynamics is very similar across all replicas in adhesion scenarios prone to **P13 CNV** and much less heterogeneous than for **T12 CNV**. Figure 11 shows typical **P13 CNV** dynamics for 10 simulation replicas of the adhesion scenario (*RRl* = 1, *RRp* = 3, *RBl* = 1, *RBp* = 2, *ROl* = 3) (Table S7, adhesion scenario ID: 83). We visualize snapshots of **P13 CNV** dynamics in one replica in Figure 12 and Video S3.

#### Early Type 2 (ET2) CNV and its progression

**Stalk cells** initially invade the **sub-retinal** space (**Early Type 2 CNV**) in three main classes of adhesion scenarios: 1) When **RPE-RPE labile adhesion** is normal (*RRl* = 3), **RPE-BrM labile adhesion** is normal or moderately impaired (*RBl*≥2), and **RPE-POS labile adhesion** is severely impaired (*ROl* = 1). 2) When **RPE-RPE labile adhesion** is severely impaired (*RRl* = 1) and **RPE-BrM labile adhesion** is either normal or moderately impaired (*RBl*≥2). 3) When **RPE-RPE**, **RPE-BrM** and **RPE-POS labile adhesion** are severely impaired (*RRl* = *RBl* = *ROl* = 1), and the combination of **RPE-RPE** and **RPE-BrM plastic coupling** satisfies *RBp+RRp*>3. Unless all **labile adhesions** are severely impaired, impairment of either **RPE-RPE** or **RPE-BrM plastic coupling** has little effect on the average *MW*, though it does increase the **CNV** initiation probability. For example, adhesion scenarios ID: 22 and 24, which differ only in their **RPE-BrM plastic coupling**, exhibit the same mean *MW*; however, *P*_init_ = 0.8 for normal **RPE-BrM plastic coupling** (ID: 22) and *P*_init_ = 1 for severely impaired **RPE-BrM plastic coupling** (ID: 24).

Figure 5 shows (1−*MW*), which measures the degree of *confinement* of **stalk cells** to the **sub-retinal** space, based on the regression-inferred average *MW* (see, the *Early Type 1 (ET1) CNV* subsection, above) as a function of the five adhesion parameters, reduced to 3D by setting *RRp* = *RRl* and *RBp* = *RBl*. The red region with (1−*MW*)>0.9, can be divided into three sub-regions: 1) When **RPE-RPE junctional adhesion** is normal, **RPE-BrM junctional adhesion** is moderately impaired, and **RPE-POS labile adhesion** is severely impaired (weak). 2) When **RPE-RPE junctional adhesion** is severely impaired (weak) and **RPE-BrM junctional adhesion** is normal, independent of **RPE-POS labile adhesion**. 3) When **RPE-RPE junctional adhesion** is severely impaired (weak), **RPE-BrM junctional adhesion** is moderately to severely impaired, and **RPE-POS labile adhesion** is severely impaired. Figure 5 does not include all the adhesion scenarios in Table S3 leading to **Early Type 2 CNV**.

**Figure 13:**
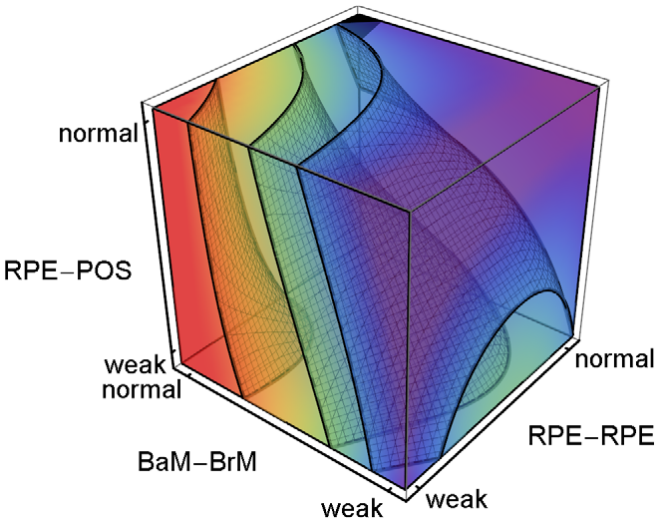
Stable Type 2 CNV dependence on adhesion. 3D plot of the regression-inferred probability of occurrence of **Stable Type 2 CNV** (**S22 CNV** probability) using 10 simulation replicas for each adhesion scenario in the 3D parameter space obtained by setting *RRp* = *RRl* and *RBp* = *RBl*. Red corresponds to a **S22 CNV** probability of 1 and purple corresponds to a **S22 CNV** probability of 0. The black region at the top-back corner indicates the locus of normal adhesion. The three isosurfaces correspond to **S22 CNV** probabilities of 0.25 (right), 0.5 (middle) and 0.9 (left). The five parameters and their (multi)linear combinations account for 89% of the observed variance in the probability of occurrence of **S22 CNV** in all 108 adhesion scenarios (adjusted *R* ^2^ = 0.84 ). **S22 CNV** occurs primarily when **RPE-RPE junctional adhesion** is moderately to severely impaired, **RPE-BrM junctional adhesion** is normal or moderately impaired, independent of **RPE-POS labile** adhesion (red region with **S22 CNV** probability>0.9). The red region does not include all adhesion scenarios in Table S8 leading to **S22 CNV**. To show the structure of the isosurfaces, we have rotated the axes relative to Figure 3.

**Figure 14:**
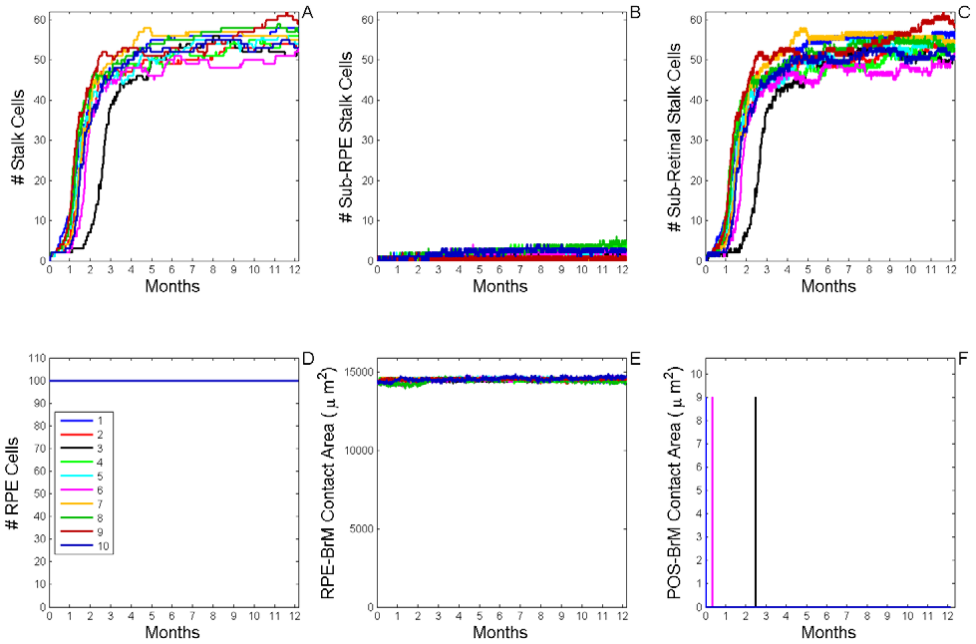
Dynamics of stable Type 2 CNV (S22 CNV). A) Total number of **stalk cells** vs. **time**. B) Total number of **stalk cells** confined in the **sub-RPE** space vs. **time**. C) Total number of **stalk cells** in contact with the **POS** (**stalk cells** in the **sub-retinal** space) vs. **time**. D) Total number of **RPE cells** vs. **time**. E) Total contact area between **RPE** **cells** and **BrM** vs. **time**. F) Total contact area between **POS cells** and **BrM** vs. **time**. The different colors represent the results of 10 simulation replicas of the adhesion scenario (*RRl* = 1, *RRp* = 1, *RBl* = 3, *RBp* = 3, *ROl* = 3) (Table S8, adhesion scenario ID: 16). (A, C) **CNV** initiates in all replicas and all develop **ET2 CNV** during the first three **months** of the simulation. All replicas exhibit **S22 CNV**. A few **stalk cells** in most replicas die due to lack of **RPE-derived VEGF-A**. (C) Few or no **stalk cells** reach the **sub-RPE** space. (D) The **RPE** remains viable in all replicas. (E) The contact area between the **RPE** and **BrM** does not change as **S22** develops. (F) The **POS** contacts **BrM** a few times, but the contact area and duration are both small, so the **RPE** does not develop any persistent or substantial holes.

**Figure 15:**
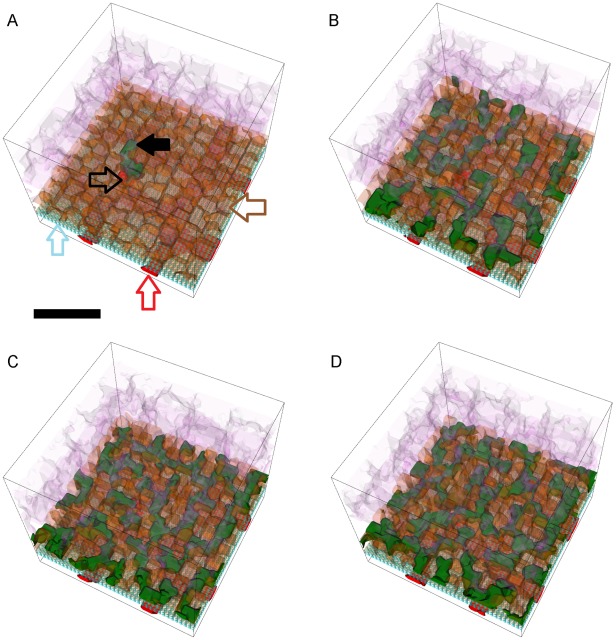
Snapshots of a simulation replica showing stable Type CNV (S22 CNV). 3D visualization of a simulation replica showing **S22 CNV** in one simulated **year** (*RRl* = 1, *RRp* = 1, *RBl* = 3, *RBp* = 3, *ROl* = 3) (adhesion scenario ID: 16, simulation ID: 556). Snapshots of the simulation at **months** 1 (A), 2 (B), 6 (C) and 12 (D). (A) **Stalk cells** (solid black arrow) invade the **sub-retinal** space through a hole in **BrM** (black outline arrow) and form a partially developed capillary network (B). **CNV** finishes **sub-retinal** invasion around **month** 5 and remains in the **sub-retinal** space throughout **LT2 CNV** (C–D). A few **vascular cells** (A, black outline arrow) fill the hole in **BrM** to connect the **CNV** capillaries to the **CC** (red outline arrow). Brown outline arrow shows an **RPE cell**. **Cell type** colors: 1) **POS** and **PIS**: light purple, 2) **RPE**: brown (**stalk cells** in the **sub-retinal** space have lighter shading), 3) **Stalk cells**: green, 4) **Vascular cells** (**CC**): red, 5) **BrM**: light blue. Scale bar ∼50 µm. We have rendered the boundaries of individual cells as semi-transparent membranes. **POS**, **PIS** and **RPE** cells are more transparent to show the underlying structures. See also Video S4.

In **Stable Type 2 CNV** (**S22 CNV**), **stalk cells** initially invade the **sub-retinal** space to develop **Early Type 2 CNV** and remain confined in the **sub-retinal** space in **Late Type 2 CNV**. Most adhesion scenarios that develop **ET2 CNV** in which the *MW* remains less than 0.15 during the first three months also exhibit **S22 CNV** (Figure 13, Table 7 and supplementary Text S5). Generally, **CNV** dynamics is very similar across all replicas of the adhesion scenarios prone to **S22 CNV** (Table 7 and supplementary Text S5). As for **P13 CNV**, the variability from replica to replica is smaller than for **S11 CNV**. Figure 14 shows typical **S22 CNV** dynamics for 10 simulation replicas of the adhesion scenario (*RRl* = 1, *RRp* = 1, *RBl* = 3, *RBp* = 3, *ROl* = 3) (Table S8, adhesion scenario ID: 16). We show snapshots of the **S22 CNV** dynamics in one replica in Figure 15 and Video S4.

**Figure 16:**
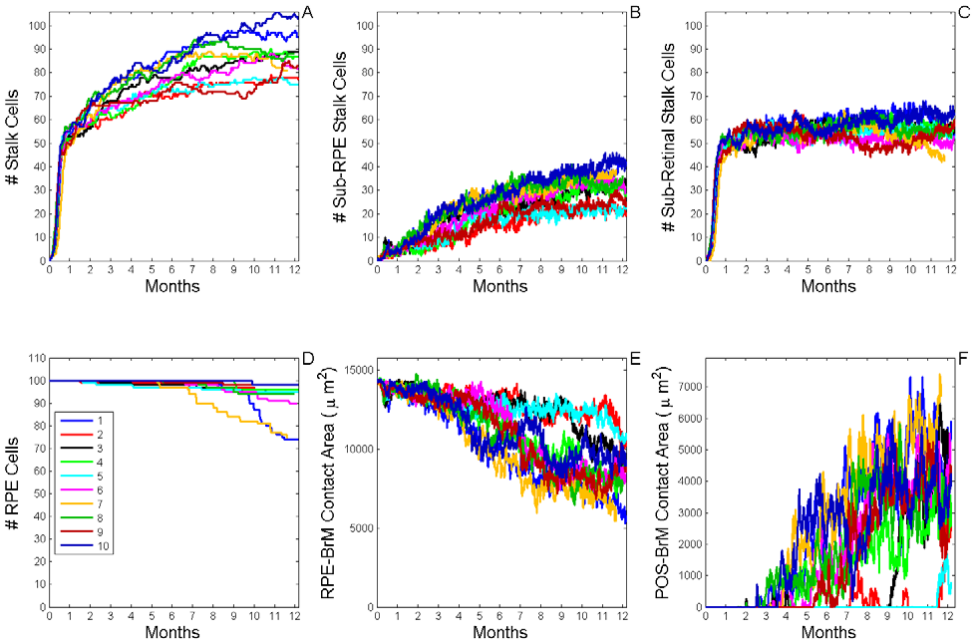
Dynamics of sub-retinal CNV to sub-RPE CNV progression (P23 CNV Progression). A) Total number of **stalk cells** vs. **time**. B) Total number of **stalk cells** confined in the **sub-RPE** space vs. **time**. C) Total number of **stalk cells** in contact with the **POS** (**stalk cells** in the **sub-retinal** space) vs. **time**. D) Total number of **RPE cells** vs. **time**. E) Total contact area between **RPE cells** and **BrM** vs. **time**. F) Total contact area between **POS cells** and **BrM** vs. **time**. The different colors represent the results of 10 simulation replicas of the adhesion scenario (*RRl* = 1, *RRp* = 1, *RBl* = 1, *RBp* = 1, *ROl* = 1) (Table S9, adhesion scenario ID: 108). **CNV** initiates in all replicas and all develop **ET2 CNV**. A few **stalk cells** in most replicas die due to lack of **RPE-derived VEGF-A**. (B) **Stalk cells** cross the **RPE** and invade the **sub-RPE** space once the number of **stalk cells** in the **sub-RPE** space reaches ∼50 **cells**, which usually occurs during the first **month** after initiation. **Stalk cells** gradually invade the **sub-RPE** space during one simulated **year**. (D) Up to 30 **RPE cells** (30% of the total) die. The number of **RPE cell** deaths increases with the number of **sub-RPE stalk cells**. (E) The contact area between the **RPE** and **BrM** decreases as **P23 CNV** develops. (F) In all replicas the **POS** contacts **BrM** persistently and extensively, as the **RPE** develops substantial holes (see Figure 17).

**Figure 17:**
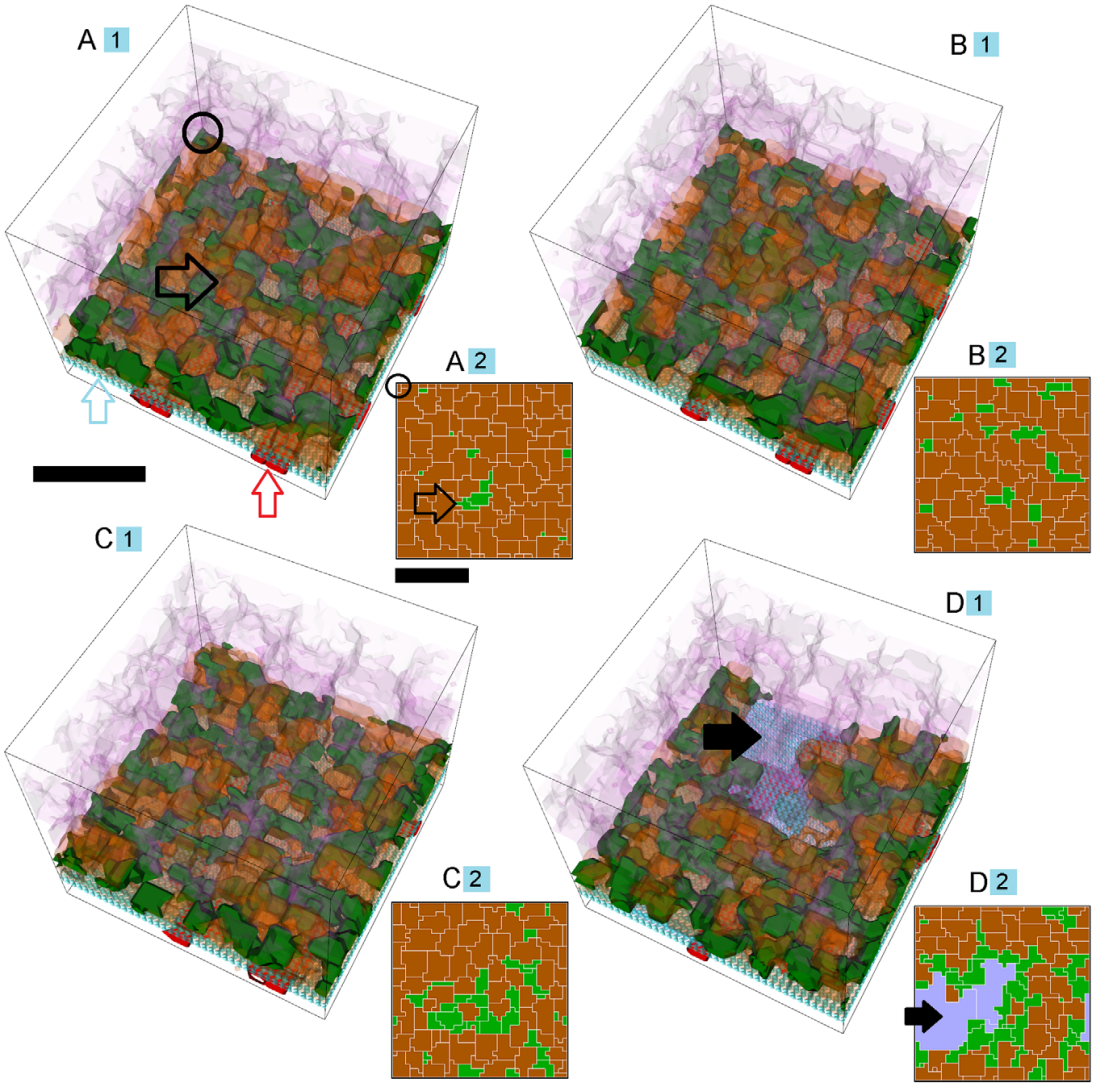
Snapshots of a simulation replica exhibiting sub-retinal CNV to sub-RPE CNV progression (P23 CNV). 3D and 2D visualization of a simulation replica forming **P23 CNV** in one simulated **year** (*RRl* = 1, *RRp* = 1, *RBl* = 1, *RBp* = 1, *ROl* = 1) (adhesion scenario ID: 108, simulation ID: 1080). Snapshots of the simulation at **months** 1 (A), 3 (B), 6 (C) and 12 (D). (A2-D2) Cross-sections of (A1-D1) parallel and adjacent to **BrM**, so **stalk cells** shown in (A2-D2) contact **BrM**. The black open circles (A1-2) at the top corner and outline back arrows (A1-2) at the location of the hole in **BrM** are guides to the eye to align A2 to A1. The alignment is consistent across all panels. (A) **Stalk cells** (solid black arrow) invade the **sub-retinal** space through the hole in **BrM** (A1-2, black outline arrows) that the **tip cell** form during the first 24 **hours** of the simulation and form a fully developed **sub-retinal** capillary network by **month** 1. (A2) Only a few **stalk cells**, mostly near the hole in **BrM**, invade the **sub-RPE** space during the first **month**. (B1, C1) The **sub-retinal** capillary network does not grow significantly. (B2, C2) Additional **stalk cells** invade the **sub-RPE** space. (D) More **stalk cells** invade the **sub-RPE** space, disrupting the **RPE** and causing a micro-tear (D1-2, black arrows). The **POS** contacts **BrM** at the location of the **RPE** tear. **Cell type** colors: 1) **POS** and **PIS**: light purple, 2) **RPE**: brown (**stalk cells** in the **sub-retinal** space have lighter shading), 3) **Stalk cells**: green (3D-visualized **stalk cells** in the **sub-retinal** space have lighter shading), 4) **Vascular cells** (**CC**): red, 5) **BrM**: light blue. Scale bars ∼50 µm. We have rendered the boundaries of individual cells in A1-D1 as semi-transparent membranes. **POS**, **PIS** and **RPE** cells are rendered more transparent to show the underlying structures. See also Video S5.

**CNV** dynamics is very similar across all replicas of the adhesion scenarios prone to **P23 CNV** (Table 7 and supplementary Text S5). Variability from replica to replica is low and comparable to the variability observed in **P13 CNV** and **S22 CNV**. Figure 16 shows typical **P23 CNV** dynamics for 10 simulation replicas of the adhesion scenario where all adhesions are severely impaired (*RRl* = 1, *RRp* = 1, *RBl* = 1, *RBp* = 1, *ROl* = 1) (adhesion scenario ID: 108). We visualize snapshots of the **P23 CNV** dynamics in one replica in Figure 17 and Video S5.

#### Early Type 3 (ET3) CNV and its progression

In **Early Type 3** (**ET3**) **CNV**, **Stalk cells** initially grow both between the **RPE** and **BrM** and between the **RPE** and the **POS**. Most adhesion scenarios have severely impaired **RPE-RPE labile adhesion** (*RRl* = 1), normal **RPE-POS labile adhesion** (*ROl* = 3) and either severely or moderately impaired **RPE-BrM labile adhesion** (*RBl*≤2) (Table S4). In these adhesion scenarios, **RPE-RPE** and **RPE-BrM plastic coupling** have little effect on mean *MW* and **CNV** initiation probability. For example, adhesion scenarios ID: 82 (*RRp* = 3), 85 (*RRp* = *2*) and 88 (*RRp* = 1) have similar mean *MW* and **CNV** initiation probability, despite differing in their **RPE-RPE plastic coupling** strengths (*RRp*).

**Figure 18:**
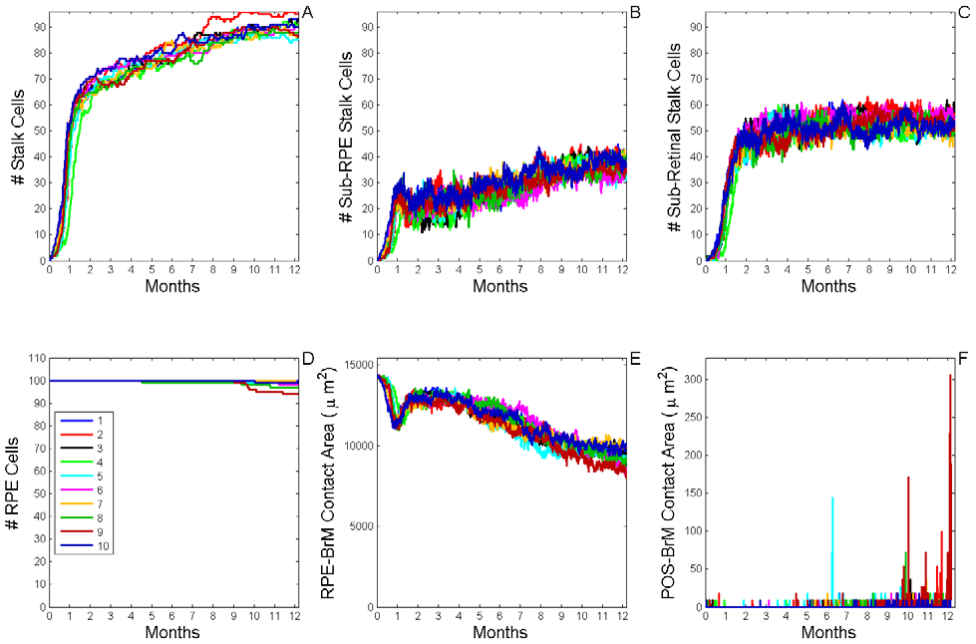
Dynamics of stable Type 3 CNV (S33 CNV). A) Total number of **stalk cells** vs. **time**. B) Total number of **stalk cells** confined in the **sub-RPE** space vs. **time**. C) Total number of **stalk cells** in contact with the **POS** (**stalk cells** in the **sub-retinal** space) vs. **time**. D) Total number of **RPE cells** vs. **time**. E) Total contact area between **RPE cells** and **BrM** vs. **time**. F) Total contact area between **POS cells** and **BrM** vs. **time**. The different colors represent the results of 10 simulation replicas of the adhesion scenario (*RRl* = 1, *RRp* = 1, *RBl* = 2, *RBp* = 2, *ROl* = 3) (Table S10, adhesion scenario ID: 53). (A, B, C) **CNV** initiates in all replicas and all replicas develop **ET3 CNV**. During the first **month** after initiation, stalk cells gradually invade both the **sub-RPE** space and the **sub-retinal** space, with more invading the **sub-RPE** space. Between **months** 1 and 2 about 30% of the **sub-RPE stalk cells** transmigrate into the **sub-retinal** space. After **month** 3, the number of **sub-RPE stalk cells** increases slowly, while the number of **sub-retinal stalk cells** remains constant. (E) During the first **month** of the simulation, the contact area between the **RPE** and **BrM** rapidly decreases as **stalk cells** invade the **sub-RPE** space. Between **months** 1 and 2, the contact area between the **RPE** and **BrM** rapidly increases as **sub-RPE stalk cells** transmigrate into the **sub-retinal** space. The contact area between the **RPE** and **BrM** slowly decreases after **month** 3 throughout the simulated **year**. (D) A few **RPE cells** die in most replicas. (F) In a few replicas the **POS** persistently contacts **BrM**, as the **RPE** develops small holes.

**Figure 19:**
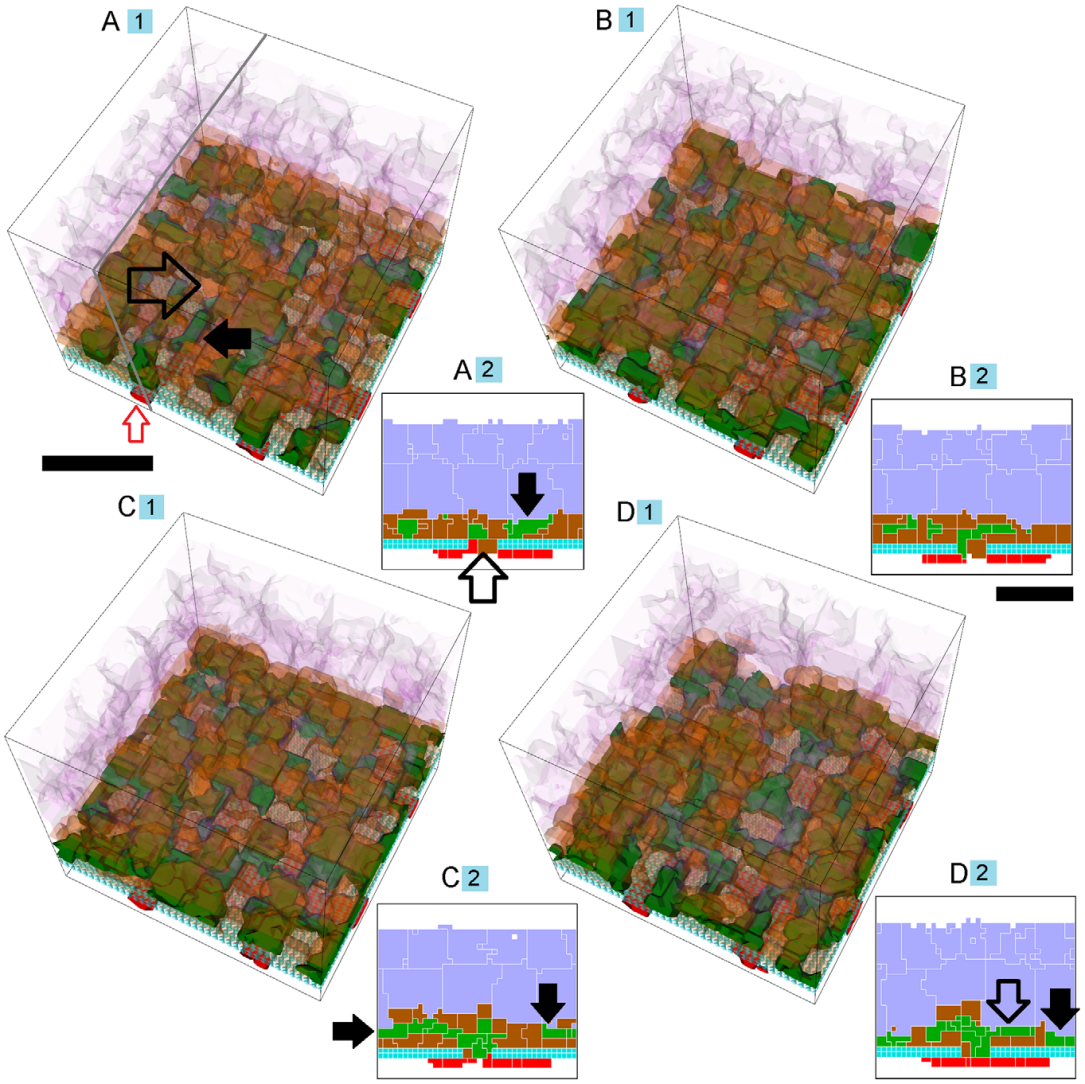
Snapshots of a simulation replica exhibiting stable Type 3 CNV (S33 CNV). 3D and 2D visualization of a simulation replica developing **S33 CNV** in one simulated **year** (*RRl* = 1, *RRp* = 1, *RBl* = 2, *RBp* = 2, *ROl* = 3) (adhesion scenario ID: 53, simulation ID: 917). Snapshots of the simulation at **months** 1 (A), 2 (B), 6 (C) and 12 (D). (A2-D2) Cross-sections of (A1-D1). All cross-section planes in (A1-D1) panels defined by the two thick black lines in A1. (A) **Stalk cells** invade the **sub-RPE** space through a hole in **BrM** (A1-2, black outline arrows) that the **tip cell** form during the first 24 **hours** of the simulation. These **stalk cells** then form a fully developed **sub-RPE** capillary network. (A2) Only a few **stalk cells** (black arrow, A1-2) reach the **sub-retinal** space during the first **month**. (B1, C1) The **sub-retinal** and **sub-RPE** capillary networks do not grow significantly. (C2) A capillary (black arrows), enveloped by a bilayer of **RPE cells**, connects the **sub-retinal** space to the **CC** via the hole in **BrM** (D) **Stalk cells** disrupt the **RPE**, forming small holes in the **RPE** (D2, black arrow). The **stalk cells** at the location of the hole in the **RPE** (D2, black arrow) contact both the **POSs** and **BrM**. The black outline arrow shows **sub-retinal stalk cells**. **Cell** **type** colors: 1) **POS** and **PIS**: light purple, 2) **RPE**: brown, 3) **Stalk cells**: green, 4) **Vascular cells** (**CC**): red, 5) **BrM**: light blue. Scale bar ∼50 µm. We have rendered the boundaries of individual cells in A1-D1 as semi-transparent membranes. **POS**, **PIS** and **RPE** cells are rendered more transparent to show the underlying structures. See also Video S6.

Generally, **CNV** dynamics is very similar across all replicas of the adhesion scenarios prone to **S33 CNV** (Table 7 and supplementary Text S5). Variability from replica to replica is comparable to the variability in **P13 CNV**, **S22 CNV** and **P23 CNV**. Figure 18 shows the typical **S33 CNV** dynamics in 10 simulation replicas of the adhesion scenario (*RRl* = 1, *RRp* = 1, *RBl* = 2, *RBp* = 2, *ROl* = 3) (Table S10, adhesion scenario ID: 53). We visualize snapshots of **S33 CNV** dynamics in one replica in Figure 19 and Video S6.

## Discussion

Our simulations show that variations in five key adhesion strengths suffice to explain many of the experimentally and clinically observed dependencies of CNV initiation on drusen, inflammation, retinal detachment and iatrogenic to reproduce the main observed types and progression dynamics of CNV associated with those defects. Since pathological conditions can cause multiple adhesion failures in the BrM-RPE-POS complex, we simulated factorial combinations of graded impairments of the five adhesion types to explore the effects of biologically-coupled adhesion failures. In this section, we discuss the effects of both individual adhesion failures and their combinations on **CNV** initiation, **type** and **dynamics** and explain their clinical and experimental relevance.

Table 1 aggregates a spectrum of clinical and experimental observations for a variety of conditions, with rough estimates of the degree of impairment of the three main inter-component adhesions present in the BrM-RPE-POS complex. Table 1 also lists the most common types of CNV associated with each condition. In this section, we compare clinical and experimental observations for these conditions to the results of our simulations for appropriate adhesion scenarios. Since we assume adhesion is constant over the duration of the simulation, to understand the effects of the gradual changes in adhesion which occur in patients as CNV develops, we must look successively at the results of multiple simulations for an appropriate series of adhesion impairments comparable at each time to those of the disease as it progresses.

### Clinical and Experimental Types of CNV and their Relation to Simulations

#### CNV due to soft drusen in older humans

As we discussed earlier (see the section *Current Hypotheses for CNV Initiation and Progression*), soft drusen significantly reduce the adhesion of the basement membrane of the RPE to BrM (RBaM-BrM adhesion). Sub-RPE CNV often starts by growing between these weakly adhered layers. Although soft drusen are seen clinically, the early stages of invasion of ECs into the sub-RPE space have little effect on visual acuity, so early EC invasions can remain unreported and unnoticed. In these patients, initial vision acuity prior to CNV initiation is not severely impaired compared to typical age-controlled visual acuity, suggesting that their photoreceptors remain healthy or at most moderately impaired compared to age-matched controls. Since the RPE plays multiple roles in maintaining both photoreceptors (*e.g.* through the phagocytosis of spent disks from the photo receptors) and the outer retina (*e.g.* through the transport of fluids), near-normal photoreceptors can only persist in the presence of near-normal RPE cells. Near-normal vision also requires that the POS remain attached to the RPE without accumulation of sub-retinal fluid or retinal detachment, indicating that RPE-POS adhesion must remain near-normal. Based on these observations, we hypothesize that in patients with soft drusen, both RPE-RPE and RPE-POS adhesion are near-normal, but BaM-BrM adhesion is impaired with the level of impairment differing patient-to-patient. This spectrum of adhesion impairments in the BrM-RPE-POS complex is comparable to the first sub-class of adhesions scenarios which are prone to **Early Type 1 CNV** (Table 6). Within this sub-class, variation in the degree of adhesion impairment of **RPE-RPE** plastic coupling, **RPE-BrM** labile adhesion and **RPE-BrM** plastic coupling affects many aspects of **CNV** in simulations, including the **CNV** initiation probability, **CNV** onset time and **CNV** dynamics. Additionally, some adhesion scenarios in this class show significant variability among simulation replicas with identical adhesivities.

Patients with multiple large soft drusen or *confluent drusen* (*soft indistinct drusen*) have a higher chance of developing CNV [74], suggesting that CNV initiation probability depends on total area of drusen and their shape. In standard clinical usage, drusen with a diameter <63 µm are called *small drusen* and those with a diameter >125 µm are called *large drusen*. Confluent drusen or soft indistinct drusen form when multiple soft drusen touch and merge. Confluent drusen strongly correlate with CNV and serous RPE detachment due to fluid accumulation between BrM and the RPE, indicating severe impairment of RBrM-BrM adhesion due to the larger area of impaired RBrM-BrM adhesion and high lipid levels in the drusen affected regions. These experimental observations suggest that the probability of CNV initiation increases with both the degree and total area of RBrM-BrM adhesion impairment. In our simulations, the total area of impaired adhesion is the same in all replicas, so it does not affect the **CNV** initiation probability. However, the **CNV** initiation probability increases with the degree of impairment of **RPE-BrM junctional adhesion** in those adhesion scenarios exhibiting **ET1 CNV** (Table 6, **ET1 CNV** first sub-class), supporting our experimentally-based hypothesis.

The locus of **stalk cells** in all simulation replicas of the first sub-class of **ET1 CNV** agrees with that in clinical and sub-clinical (sub-clinical CNV appears in histology but not clinically) sub-RPE CNV, the typical early CNV type in these patients. It also agrees with the appearance of drusen-like deposits and the initial stages of EC invasion into BrM observed in a fat-fed aged-mouse model (16 months old) exposed to non-phototoxic levels of blue light [75]. However, CNV did not progress in this mouse model [75].

While experimental and clinical results are incomplete, as we discussed above, they do suggest that patients with stable sub-RPE CNV do not suffer high rates of severe RPE detachment, indicating that their RBaM-BrM adhesion is not severely impaired. Among simulations with **ET1 CNV**, those in which **RPE-BrM junctional adhesion** is not too severely impaired (Table 7, *RBl* = 1 or 2 and 3≤*RBl+RBp*≤4), exhibit **Stable Type 1 CNV**, agreeing with our interpretation of this clinical observation.

Since we hypothesize that more severe impairment of RBaM-BrM adhesion facilitates CNV spreading and progression, we expect higher variability of outcomes in patients with moderately impaired adhesion than in patients with severely impaired adhesion. In older patients, CNV progression timing and the size and growth rate of the CNV-affected area vary significantly patient-to-patient [5], [76]–[78]. About one third of untreated sub-RPE CNV cases in older patients remain stable for extended periods of time (∼30% remain stable 3 years after diagnosis). A slightly larger population of apparently similar patients progress to more damaging sub-retinal CNV in a short period of time (∼40% develop sub-retinal CNV within the 12 months after diagnosis). According to a different study [76], sub-RPE CNV lesion size doubles in 12 months in ∼30% of patients and quadruples in ∼40% of patients and remained stable in the remainder. Such patient-to-patient variability corresponds closely to the variability we observe in **stalk cell** proliferation, **CNV** area growth and onset time in our corresponding simulations. As in clinical observations, simulations of adhesion scenarios with moderately impaired **RPE-BrM junctional adhesion** (Table 7, *RBl* = 1 or 2 and 3≤*RBl+RBp*≤4,) have greater variability in stalk cell proliferation, CNV growth rate and CNV onset time than simulations with severely impaired **RPE-BrM junctional adhesion** (Table 6, *RRl* = 3, *RRp* = 3, *RBl* = 1, *RBp* = 1, *ROl* = 3). For example, in the 10 simulation replicas of an adhesion scenario with moderately impaired **RPE-BrM junctional adhesion** and all other adhesion normal (Table 7, *RRl* = 3, *RRp* = 3, *RBl* = 2, *RBp* = 2, *ROl* = 3) (Figure 7) nine out of ten replicas initiate **CNV**, but the total number of stalk cells after one simulated **year** shows a four-fold variation (from a minimum of 10 **stalk cells** to a maximum of 45 **stalk cells**) and the **CNV** onset time varies from a minimum of two **weeks** to a maximum of 4 **months** (after formation of the initial hole in **BrM**). For less impaired adhesion, when the **plastic** component of **RPE-BrM junctional adhesion** is severely impaired but the **labile** component is normal (Table S1, *RRl* = 3, *RRp* = 3, *RBl* = 3, *RBp* = 1, *ROl* = 3), **CNV** initiates in only 30% of simulations, the initiation onset time is around 10 **months** (after formation of the initial hole in **BrM**) and the total number of **stalk cells** remains less than 6 in all replicas during the simulated **year**.

Overall, the **stalk cell** growth and proliferation rate among simulations that develop **ET1 CNV**, is slowest in those simulations exhibiting **S11 CNV** and fastest in those simulations exhibiting **P13 CNV**. The **stalk cell** division rate (the frequency of **stalk cell** division within the tissue) in **S11 CNV** ranges from one or two cell divisions per **year** to one cell division every 48 **hours**. The **stalk cell** division rate in **P13 CNV** is less variable, about one cell division every ∼12 **hours** during **ET1 CNV** (Figure 11A, the first **month** in most replicas). The long initiation delays and slow sub-RPE CNV development observed in some of our simulation replicas correspond to the slow sub-RPE CNV development and long-term CNV stability observed in a small population of patients (<30% as mentioned in [78]). Fast-progressing **ET1 CNV** in some of these simulations agrees with the clinically-observed rapid increase in sub-RPE CNV size in a different set of otherwise similar patients. Based on the observed variability in **CNV** onset time and growth rate in our simulations and its dependence on the degree of adhesion impairment, we believe that the observed clinical patient-to-patient variability may result from both intrinsic stochasticity in certain adhesion regimes and from small patient-to-patient differences in the degree and type of adhesion impairments in the patient's BrM-RPE-POS complex.

In older patients, sub-RPE CNV may later also invade the sub-retinal space (sub-RPE CNV to CNV sub-retinal progression is a common CNV progression scenario). The factors involved in this transition are not well understood. Gradual degradation of the RPE due to sub-RPE hemorrhaging, formation of sub-RPE fibrosis and inflammation triggered by initial sub-RPE CNV are associated with this transition. The death of RPE cells during this degradation indicates that RPE-RPE adhesion is impaired. The rapid vision loss associated with the transition from sub-RPE CNV to sub-retinal CNV, indicates impaired RPE-POS adhesion, though, clinically, we do not know whether RPE-POS adhesion impairment is a cause or result of the transition. Thus these conditions imply impairment of **RPE-RPE junctional adhesion** and/or **RPE-POS labile adhesion** in addition to preexisting impairment of **RPE-BrM junctional adhesion** (due to lipid accumulation). Three classes of adhesion scenarios are relevant: 1) When both **RPE-POS labile adhesion** and **RPE-BrM junctional adhesion** are impaired, 2) when both **RPE-RPE** and **RPE-BrM junctional adhesion** are impaired, and 3) when **RPE-RPE junctional adhesion**, **RPE-POS labile adhesion**, **RPE-BrM junctional adhesion** are impaired. These three classes include all the sub-classes of adhesion scenarios leading to **T12 CNV**, **P13 CNV**, **S22 CNV**, **S23 CNV** and **S33 CNV**. These three classes never lead to **S11 CNV** (for definitions of the sub-classes of adhesion scenarios see Table 7), suggesting that impairment of RPE-RPE and/or RPE-POS adhesion in addition to preexisting impairment of RBaM-BrM in patients is the primary mechanism leading to the sub-RPE CNV to sub-retinal CNV transition.

Clinically, adhesion strengths may change as CNV progresses. However our simulations show that P13 CNV progression can occur in patients even for time-independent adhesion. We can thus use our simulations to develop a prognosis for patients with sub-RPE CNV and in whom RPE-RPE and/or RPE-POS adhesion are impaired in addition to preexisting impairment of RBaM-BrM. Simulations that exhibit **ET1 CNV** and later invade the sub-retinal space in **P13 CNV** (Table 7, *RRl* = 1, independent of *RRp*, *RBl* = 1, *RBp* = 1 or 2, *ROl* = 3) correspond to the most common clinically observed progression of AMD-induced CNV, which begins as sub-RPE CNV and later progresses to involve the sub-retinal space. Simulations with **T12 CNV** (Table 7, *RRl* = 3, *RBl* = 1, *ROl* = 1, *RRp+RBp*≥4 except *RRp* = *RBp* = 2) do not appear to correspond to any standard clinical CNV progression dynamics, perhaps, because the transient nature of the sub-RPE to sub-retinal translocation makes its clinical detection difficult; we hypothesize that T12 CNV may be occurring but is not being diagnosed. Clinically, depending on the time of observation, T12 CNV could be diagnosed as sub-RPE CNV, Type 3 or sub-retinal CNV. Only frequent prospective eye examinations and long-term follow up can determine whether our prediction of clinical sub-RPE to sub-retinal translocations is correct.

Clinically, ET2 and ET3 CNV are not common in drusen-induced CNV in patients. However, the adhesion scenarios that exhibit **ET2** and **ET3** in our simulations might correspond to secondary CNV which develops later to a primary site of CNV in patients with pre-existing ET1 CNV. To explore the relevance of these scenarios, we could conduct simulations beginning with pre-existing **ET1 CNV** instead of a single **tip cell** in the **CC**. For example, we would expect the pre-existing **ET1 CNV** to translocate to the **sub-retinal** space when simulated in adhesion scenarios that exhibit primary **S22 CNV**. Such CNV dynamics would look like **T12 CNV** (discussed above). Clinically, this result implies that if we were to increase RBaM-BrM adhesion therapeutically (*e.g.* by extraction of lipids from BrM or by removing/reducing fluids between RBaM and BrM) in the presence of pre-existing sub-RPE CNV, stalk cells could translocate to the sub-retinal space in a transition to sub-retinal CNV. Since sub-retinal CNV is much more damaging to vision than sub-RPE CNV, this translocation would be a serious iatrogenic side effect. We can make similar analogies for the significance of simulated **S23 CNV** and **S33 CNV**.

Vascular RPE detachment caused by growth of CNV under the RPE is a common complication of sub-RPE CNV in AMD. We observe a corresponding pathology when **RPE-BrM junctional adhesion** is severely impaired (Table 6, **Early Type 1** sub-class 1, *RBl* = *RBp* = 1). In this case, **Early Type 1 CNV** results in later **RPE** detachment, leading to either **T12 CNV** translocation or **P13 CNV** progression in conjunction with the formation of holes in the RPE (we will discuss late-stage CNV complications in detail in future papers).

#### Inflammation-induced CNV

Sub-retinal CNV without prior diagnosed sub-RPE CNV occurs, but is not as common in older patients as drusen-induced CNV [79]. It also occurs in young patients in conjunction with acute inflammatory conditions, particularly in cases of serpiginous choroidopathy, multifocal choroiditis and panuveitis [80]. The mechanisms leading to this type of CNV are not well understood and we lack clinical insight into how specific risk factors affect the probability of CNV initiation. Younger patients usually lack both drusen [80] and significant dispersed build up of lipids in BrM. As we discussed earlier active inflammation reduces RPE-RPE epithelial adhesion. Alteration of RPE-RPE epithelial adhesion combined with edema due to active inflammation may also reduce RPE-POS adhesion. We do not know whether inflammation always impairs RPE-RPE and RPE-POS adhesion at the same time, or to the same degree. Since CNV initiation occurs promptly during acute inflammation, we do not expect acute inflammation to decrease RBaM-BrM adhesion significantly. Thus, in young patients inflammation both RPE-RPE epithelial adhesion and RPE-POS adhesion are impaired, but not RBaM-BrM adhesion. This adhesion impairment corresponds to impairments of **RPE-RPE junctional adhesion** and **RPE-POS labile adhesion**, while **RPE-BrM junctional adhesion** is near-normal (Table 6, **Early Type 2 CNV** sub-class 1 and 2). Simulations of these adhesion scenarios consistently exhibit **Early Type 2 CNV** (**ET2 CNV**), in which **stalk cells** initially invade the **sub-retinal** space without prior invasion of the **sub-RPE** space, in agreement with clinically-observed sub-retinal CNV in these patients.

In our simulations, when **RPE-RPE labile adhesion** is severely impaired and all other adhesions are normal (*RRl* = 1, *RRp* = *RBl* = *RBp* = *ROl* = 3) (Table S8, adhesion parameter set ID: 10) **CNV** always initiates, followed by **Early Type 2 CNV** (**ET2 CNV**). When **RPE-POS labile adhesion** is severely impaired and all other adhesions are normal (*RRl* = *RRp* = *RBl* = *RBp* = 3, *ROl* = 1) (Table S8, adhesion parameter set ID: 19), the **CNV** initiation probability is 50% and initiation always leads to **ET2 CNV**. When both **RPE-RPE junctional adhesion** and **RPE-POS labile adhesion** are impaired **CNV**, always initiates and leads to **ET2 CNV** (see also Figure 5, for more information on how **sub-retinal CNV** depends on adhesion). Our results make two predictions that can be tested against clinical and experimental observations: 1) Disruption of RPE-RPE epithelial junctions, due to inflammation, by itself should lead to sub-retinal CNV in patients with a relatively intact retina, independent of any defects in RPE-POS adhesion 2) Disruption of RPE-POS contact, by itself, should increase the probability of developing sub-retinal CNV.

Our understanding of CNV dynamics in young patients is incomplete. Neither sub-retinal CNV to sub-RPE CNV progression (P23 CNV) nor sub-retinal CNV to sub-RPE CNV translocation (T21 CNV) has been observed clinically or histologically (since CNV is not fatal, histological data for young patients with CNV is rare). We currently do not know whether this absence of observation is due to major retinal damage due to sub-retinal CNV, which precludes the later transition (and is not included in our model), or whether Late Type 1 CNV is simply overlooked clinically because sub-retinal CNV causes much more severe vision loss. Our simulations make three predictions relevant to inflammation-induced CNV in young patients: 1) If **RPE-BrM adhesion junctional** is near normal (*RBl* = 2 or 3, independent of *RBp*) any combination of severely impaired **RPE-RPE junctional adhesion** (*RBl* = 1, independent of *RBp*) and **RPE-POS labile adhesion** (*ROl* = 1) will exhibit **Stable Type 2 CNV**. 2) When all adhesions are severely impaired (*RRl* = *RBl* = *RBp* = *ROl* = 1, *RRp* = 1 or 2), **Sub-retinal CNV** to **sub-RPE CNV** progression (**P23 CNV**) will occur. 3) **Sub-retinal CNV** to **sub-RPE CNV** translocation (**T21 CNV**) is unlikely. These predictions mean that once CNV invades the sub-retinal space, it will not leave this space, so CNV lesions will expand primarily in the sub-retinal space as long as RBaM-BrM is not severely impaired.

Chemotoxicity (10% solution of naphthalene force-fed by gavage for 5 weeks) in a rabbit model [81] causes degradation of photoreceptors and proliferation of RPE leading to sub-retinal CNV. In this rabbit model, the RPE cells proliferating phagocytose damaged photoreceptors during the first three weeks. Sub-retinal CNV follows about 3 months after the beginning of treatment. Because RPE cells with normal epithelial adhesion do not proliferate, we hypothesize that chemotoxicity in this animal model reduces RPE-RPE epithelial adhesion, allowing RPE cells to proliferate. Since the photoreceptors are damaged, we infer that RPE-POS adhesion is severely disrupted. BrM remains intact for the first 3 weeks of treatment. Initial signs of BrM invasion by RPE cells and ECs appear about 3 months after the beginning of treatment. Due to newly synthesized extracellular matrix at the location of the CNV, the RPE basement membrane and BrM become irregular after 6 months, with bundles of extracellular microfilaments connecting RPE basement membrane to BrM. These modifications of the RPE basement membrane and BrM, suggest that RBaM-BrM adhesion is initially normal and gradually decreases to moderately impaired over a period of six months. These adhesion impairments resemble adhesion impairments in younger patients with inflammation. For these adhesion scenarios (Table 6, **Early Type 2 CNV** sub-class 1 and 2) our simulations always predict early **sub-retinal CNV**, in agreement with experiments.

#### Iatrogenic CNV

Iatrogenic sub-retinal CNV may develop after laser photocoagulation treatment of diabetic macular edema, central serous retinopathy, proliferative diabetic retinopathy, choroidal vascular and neoplastic lesions, vascular occlusive disease and degenerative retinal-pigment-epithelium disorders (reviewed in [82]). Some believed that the primary mechanism for such iatrogenic induction of sub-retinal CNV to be the creation of breaks in Bruch's membrane, with inflammatory cells, angiogenic factors and choroidal ischemia contributing to the development of CNV in some cases [82]. However, we believe that RPE phototoxicity due to excess (focal) laser exposure is more likely primary cause. Phototoxicity stresses RPE cells which can decrease RPE-RPE epithelial adhesion and RPE-POS adhesion and also promote excess expression of VEGF-A by RPE cells. This condition of pathologies is similar to those caused by inflammation, so we would expect these classes of iatrogenic CNV to resemble inflammation-induced CNV, as is indeed observed.

#### CNV due to sub-retinal injections in animal models

Subretinal injections in most animal models (Table 1) lead to sub-retinal CNV adjacent to the site of injection within weeks, but not to sub-RPE CNV [83]–[85]. CNV initiation probability depends on the type and amount of material injected (Matrigel, polystyrene beads suspended in liquid, vitreous humor, …) (see [46], for a detailed review). Secondary sub-RPE CNV may follow sub-retinal CNV in some animal models, *e.g.*, in a rabbit model [86], a sub-retinal injection of a cocktail containing endotoxins and growth-factors, incorporated in heparin-sepharose leads rapidly to primary sub-retinal CNV and secondary sub-RPE CNV forms farther from site of injection between 2 weeks and 8 months later [86]. The mechanisms leading to this secondary sub-RPE CNV are not well understood. Ni et al. [86] believed that the major factors were changes in RPE cell function due to diffusion of soluble mediators originating from the area of primary CNV, *e.g.* from atrophied primary RPE cells or newly-formed activated secondary RPE cells or changes in function of endothelial cells, photoreceptors and Müller cells in regions peripheral to the primary CNV, *e.g.*, increased expression of FGF-1, FGF-2, TGF-alpha and VEGF.

Subretinal injections can cause acute physical retinal detachment, instantaneously destroying RPE-POS contact at the site of injection. However, RPE active pumping and passive flow gradually remove the excess sub-retinal fluid, allowing the retina to reattach within a few days. Sub-retinal injection also almost always induces significant inflammation, which gradually reduces RPE-RPE epithelial adhesion over a period lasting a few days to a few weeks. Such condition of transient detachment and followed by long-lasting inflammation induces RPE migration [83] and proliferation [86] (*RPE activation*). Because RPE cells neither proliferate nor migrate when they are in epithelium and RPE-RPE epithelial adhesion remain normal, the combination of RPE migration and proliferation suggest that RPE-RPE epithelial adhesion may decrease significantly over a period of a few days to a few weeks. Inflammation can also cause the death of photoreceptors [81], suggesting prolonged disruption of RPE-POS contact. Sub-retinal injections can also directly cause focal rupture in all layers of BrM, suggesting that RBaM-BrM adhesion is initially (near) normal at least far from the location of the rupture.

Based on these experimental observations, sub-retinal injections appear mainly to impair RPE-RPE and RPE-POS adhesion comparable to the adhesion scenarios prone to **Early Type 2 CNV** (Table 6, **Early Type 2 CNV** sub-class 1 and 2) and in inflammation-induced CNV in younger patients. Thus our simulations both agree with the cause, initiation and progression of sub-retinal CNV in these animal models.

Our simulations predict that for secondary sub-RPE CNV to develop near a pre-existing sub-retinal CNV RPE-BrM (RBaM-BrM) adhesion must be severely impaired, to develop, independent of the degree of impairment of other types of adhesion in the BrM-RPE-POS complex. To validate our prediction, experiments would need to examine in detail the interface between BrM and the RPE basement membrane in retinal regions far from site of injection and before any initiation of sub-RPE CNV.

In these animal models, sub-retinal injection often causes rapid (less than a month) CNV initiation. We can infer that injection impairs RPE-RPE adhesion because of observed inflammation and RPE proliferation and RPE-POS adhesion because of photoreceptor degradation [81], [83], [86]. In agreement with these experiments, our simulations show that severe impairment of **RPE-RPE labile adhesion**, when all other adhesions are normal (*RRl* = 1, *RRp* = *RBl* = *RBp* = *ROl* = 3) (Table S8, adhesion parameter set ID: 10), increases the probability of **CNV** initiation within a **month** to 100%. In our simulations prolonged impairment of **RPE-POS labile adhesion** when all other adhesions are normal (*RRl* = *RRp* = *RBl* = *RBp* = 3, *ROl* = 1) (Table S8, adhesion parameter set ID: 19) increases initiation probability to 50%, higher than observed for experimental RPE-POS detachment. This discrepancy may result from the long (up to six **month**) onset time for **CNV** in this adhesion scenario. Six months is much longer than the CNV initiation time due RPE-RPE adhesion impairment and the typical time for retinal reattachment and is too long to see in most experiments. Experiments exploring the effects of RPE-POS adhesion impairment on CNV would need to disrupt RPE-POS adhesion for several months without inducing severe inflammation, *e.g.* by sub-retinal injection of neutral polystyrene microbeads (coated with anti-inflammation compounds).

#### The role of overexpression of VEGF

We now consider how local cytokines and growth factors that can increase the chemotactic activity of endothelial cells could affect CNV initiation and progression. Our simulations did not explore the role of these factors on the chemotactic activity of ECs directly. However, our simulations using different VEGF-A levels suggest that increased chemotactic activity of **stalk cells** should increase the **CNV initiation** probability, as in our simulations in which **RPE** expresses **RPE-derived VEGF-A** at twice the normal level. We would also expect that if these factors were short-diffusing and thus formed local gradients, they should promote sub-RPE CNV if their source were localized in the sub-RPE space, or promote sub-retinal CNV if their source were localized in the sub-retinal space. If these factors were long-diffusing, we would expect that the resulting global increase in chemotactic activity of **stalk cells** would not affect either early or late CNV loci, only the probability of initiation and the rate of progression.

In transgenic mice with inducible expression of VEGF in their RPE cells, induction of excess VEGF only induces CNV if combined with sub-retinal injections which disrupt the RPE [13]. In our simulations, when **RPE** overexpresses **RPE-derived VEGF-A** at twice the normal level, the probability of CNV initiation increases (data not shown), but the **Early CNV** types and **CNV** dynamics do not change. Thus, our simulations show that VEGF overexpression can increase the CNV initiation probability, but does not determine either the early or late CNV loci.

In experiments, ocular hypoxia caused by systemic hypoxia usually promotes retinal angiogenesis, but has no observed effect on the RPE and does not induce choroidal angiogenesis (reviewed in [40]). We do not know what levels of PO_2_ can trigger hypoxic signaling by RPE cells. Based on experimentally-measured parameters, our simulations show that the PO_2_ at the RPE decrease from ∼65 mmHg to ∼49 mmHg when the PO_2_ at the CC decreases from 80 mmHg to 60 mmHg during systemic hypoxia in the anatomically normal retina (under light-adapted condition). If we assume that biological RPE cells are hypoxic during systemic hypoxia, then the PO_2_ below which RPE cells become hypoxic is 49 mmHg. However, PO_2_∼50 mmHg is significantly higher than both PO_2_∼20 mmHg, the typical PO_2_ in the inner retina and PO_2_∼1 mmHg, the PO_2_ at which mitochondria work at their maximum metabolic rate. In our simulations, **RPE cells** become hypoxic (PO_2_<49 mmHg) only after **RPE** detachment, a CNV-associated complication which we will discuss in a future paper. Thus, neither the threshold for RPE hypoxia nor RPE hypoxic signaling affects the results we present in this paper.

#### The nature of the BrM-RPE-POS complex barrier to CNV (activated ECs)

As we discussed in the *Angiogenic and Antiangiogenic factors* section in supplementary Text S1, activated endothelial cells are present in the normal choriocapillaris, so the frequency of ECs crossing BrM is significant even in the normal eye. Clinically, the probability of CNV initiation before age 50 in a normal retina is negligible. Our adhesion-based hypotheses for CNV initiation and progression may resolve this discrepancy. In simulations when all adhesions are normal (referenced to a normal human eyes aged less than 50 years old) activated **ECs** and small holes in **BrM** never initiate CNV, suggesting that strong adhesion among BrM-RPE-POS components is the crucial mechanism preventing activated **ECs** from invading the **sub-RPE** and **sub-retinal** space once they have crossed BrM.

### Future Directions and Suggestions

Our current model does not include several mechanisms which may also be important to CNV. In future refinements, we will include multiple types of basal deposits and fibrosis (synthesis of new ECM) explicitly to clarify their role in the initiation and progression of CNV. We particularly are interested in how differences in the size and structure of soft and hard drusen affect the initiation and progression of CNV and the frequency of occurrence of RPE detachment and RPE tear formation after therapeutic intervention to treat CNV.

Many hypothesized mechanisms for CNV initiation and progression involve irregularities in transport. We plan more realistic models including capillary maturation, blood flow and retinal-CC fluid flow to study how oxygen, nutrient and waste transport promote or inhibit CNV.

Since cell adhesion is essential to multicellularity and is important in embryonic development, homeostatic maintenance of adult tissues and diseases like cancer, its importance in CNV is, perhaps, not surprising, given CNV's many parallels with tumor-induced angiogenesis. However, *the role of adhesion in CNV has not been widely appreciated*, so neither the relationship between known CNV risk factors and specific adhesion failures, nor the actual adhesivities in the retina and RPE have been quantified experimentally. Quantitative measurements of these adhesion properties and their regulation in the normal and pathological retina would allow more clinically relevant models. Such experiments would greatly reduce uncertainty in our model definition, improve our understanding of CNV initiation and progression. Since measuring adhesivities directly may be difficult, especially in humans, our model also allows us to quantify adhesion failures by looking at how they affect CNV initiation and progression and matching those computational outcomes with experimental observations, then correlating the simulated adhesion changes with experimental risk factors.

Beyond retinal CNV, our results on CNV initiation and epithelial breakdown apply to any tissue in which a basement membrane separates a capillary network from a nearby epithelium, *e.g.* lung and gut. We expect that the relationships between specific classes of adhesion failures and the loci and dynamics of CNV which we observe in our simulations should carry over to the neovascularization-dependent pathologies of those tissues.

Ultimately, a database of verified simulations for different adhesion scenarios might allow systematic CNV prediction based on clinically-measurable properties of the eye, especially if the adhesion properties can be inferred noninvasively, *e.g.* by measuring optically, changes in CC or RPE morphology or autofluorescence due to lipid accumulation. In the absence of direct measurements of adhesivity, our simulations allow us to infer adhesion defects from pathologies. For example, if a patient with a functional retina exhibits micro-detachments of the RPE (due to lipid accumulation or small soft drusen), the relationship between the number and degree of detachments and the underlying classes of adhesion failures predicts the probability of CNV initiation and the distribution of CNV onset times, making clinically-useful suggestions for frequency of follow-up examinations and possible prophylactic interventions. To aid diagnosis and treatment we can also develop statistical analyses of the most significant scenarios capable of producing the observed pattern of disruption in each patient (including the probability of initiation, the time of onset of progression and progression speed,…). Follow-up observations could then narrow the range of admitted hypothetical scenarios to improve the accuracy of the prognosis. More accurate diagnoses may improve both administration of drugs and disease management.

One crucial aspect of a model-based approach to CNV diagnosis, prognosis and treatment is that both the simulation database and the statistical predictors could be continuously refined using feedback from both clinical and histopathological sources, so they would improve with use, providing a platform to integrate clinical and histopathological data for even more accurate diagnosis and prognosis.

## Methods

Our simulations use the Glazier-Graner-Hogeweg model (*GGH*, also known as the Cellular Potts Model, *CPM*), a multi-cell, lattice-based, stochastic methodology which describes biological cells and their interactions in terms of *Effective Energies* and constraints [87]. GGH applications include models of vascular tumors [65], avascular tumor growth [88], biofilms [89], chick limb growth [90], somitogenesis [91], blood flow and thrombus development [92] and angiogenesis [66], [71], [93], [94]. See supplementary Text S3 for details of implementation of objects and processes listed in Table 2. Tables 8–12 summarize the key variables and parameters in our simulations (see also Text S6 for simulation files used to run replicas of adhesion scenario ID: 38).

**Table 8:** Geometrical and transport parameters.

Geometrical Parameters		
Name	Description	Values
*L*_OS_	**POS** layer thickness	∼30 µm (compare to [97])
*L*_IS_	**PIS** layer thickness	∼24 µm (compare to [97])
*L*_OLM_	Location of OLM measured from **RPE** side of **BrM**	∼67 µm (compare to [97])
*L*_BrM_	**BrM** thickness	6 µm (compare to [24])
*L*_RPE_	**RPE** thickness	12 µm (compare to [24])

**Table 9 pcbi-1002440-t009:** Field object names.

Fields		
Name	Description	Units
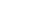	**Oxygen** partial pressure at 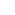	mmHg
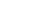	**RPE-derived VEGF-A** at 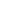	molecule/voxel
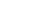	**Short-diffusing VEGF-A** secreted by **ECs** at 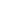	molecule/voxel
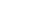	**MMP** secreted by **tip cells** at 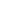	molecule/voxel

**Table 10 pcbi-1002440-t010:** Labile adhesion parameters (contact energies).

Cell Types	Stalk	BrM	RPE	POS	PIS	Medium
**Stalk**	−20	−10	−10	−10	−10	3
**BrM**		−12	−38/−28/−18	0	0	−1
**RPE**			−40/−18	−16/−1	−16/−1	3
**POS**				−16	−16	3
**PIS**					−16	3
**Medium**						0

Negative contact energies represent adhesive interactions; positive contact energies represent repulsive interactions. More negative contact energies indicate stronger adhesive interactions. (/) separates the reference, moderately impaired and severely impaired levels of **labile adhesion**.

**Table 11 pcbi-1002440-t011:** Labile adhesion strengths (contact energies).

		Labile Adhesion Strength		
Cell-Type Pairs	Name	Normal: 3	Moderately Impaired: 2	Severely Impaired: 1
**RPE-RPE**	*RRl*	−40	-	−18
**RPE-BrM**	*RBl*	−38	−28	−18
**RPE-POS**	*ROl*	−16	-	−1
**POS-POS**	**-**	−16	-	-
**PIS-PIS**	**-**	−16	-	-

More negative contact energies indicate stronger adhesive interactions. (-) denotes **labile adhesion** strengths not used in our simulations.

**Table 12:** Plastic coupling strengths (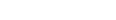) links between cell-type pairs.

		Plastic Coupling Strength		
Cell-Type Pairs	Name	Normal: 3	Impaired: 2	Severely Impaired: 1
**RPE-RPE**	*RRp*	300	60	30
**RPE-BrM**	*RBp*	300	60	30
**POS-POS**	-	30	-	-
**PIS-PIS**	-	30	-	-
**PIS-POS**	-	30	-	-
**Vascular-Vascular**	-	200	-	-
**Stalk-Vascular**	-	150	-	-
**Tip-Vascular**	-	50	-	-
**Stalk-Stalk**	-	50	-	-
**Stalk-Tip**	-	50	-	-
**Vascular-BrM**	-	200	-	-
**Stalk-BrM**	-	25	-	-
**Tip-BrM**	-	25	-	-

Larger plastic coupling strengths represent stiffer linear springs. (-) denotes values of 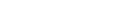 not used in our simulations.

### Implementation

All our simulations use the open-source CompuCell3D simulation environment (http://www.compucell3d.org/) [95]. We ran our simulations on a computer cluster (Quarry, Indiana University) using CompuCell3D v3.4.2. A typical simulation replica takes about 30 hours on a single core of a 2.0 GHz quad-core Intel Xeon 5335 processor. We stored the **cell** and **field**-lattice configurations every 6 simulated **hours** and rendered each snapshot using the integrated post-processing rendering provided by CompuCell3D.

## Supporting Information

Table S1 **Adhesion Scenarios with Infrequent or No CNV Initiation.**
(PDF)

Table S2 **Adhesion Scenarios Prone to Early Type 1 CNV (MW>0.9) if CNV Initiates.**
(PDF)

Table S3 **Adhesion Scenarios Prone to Early Type 2 CNV (MW<0.05) if CNV Initiates.**
(PDF)

Table S4 **Adhesion Scenarios Prone to Early Type 3 CNV (0.35<MW<0.65) if CNV initiates.**
(PDF)

Table S5 **Adhesion Scenarios Prone to Stable Type 1 CNV (S11 CNV Probability>0.9).**
(PDF)

Table S6 **Adhesion Scenarios Prone to Sub-RPE to Sub-Retinal Translocation (T12 Translocation).**
(PDF)

Table S7 **Selected Adhesion Scenarios Prone to Sub-RPE to Sub-Retinal Progression (P13 Progression) (P13 Probability>0.7).**
(PDF)

Table S8 **Adhesion Scenarios Prone to Stable Type 2 CNV (S22) (S22 Probability>0.9).**
(PDF)

Table S9 **Adhesion Scenarios Prone to Sub-Retinal CNV to Sub-RPE CNV Progression (P23 CNV) (P23 Probability>0.6).**
(PDF)

Table S10 **Adhesion Scenarios Prone to Stable Type 3 CNV (S33 CNV) (S33 Probability>0.9).**
(PDF)

Text S1 **Biological Components and Processes in CNV.**
(PDF)

Text S2 **Simplifying Assumptions of Our Model.**
(PDF)

Text S3 **GGH Implementation of the Multi-cell Model.**
(PDF)

Text S4 **Modeling Terminology.**
(PDF)

Text S5 **CNV Progression Dynamics.**
(PDF)

Text S6 **CompuCell3D Code to Simulate Adhesion Scenario ID: 38.**
(ZIP)

Video S1 **Time-Series of a Simulation Replica with Stable Type 1 CNV.** 3D visualization of a simulation replica exhibiting **Stable Type 1 CNV** over one simulated **year** (adhesion scenario ID: 38, simulation ID: 902) (*RRl* = 3, *RRp* = 3, *RBl* = 2, *RBp* = 2, *ROl* = 3). **Stalk cells** invade the **sub-RPE** space through a hole in **BrM** that the **tip cell** opens during the first 24 **hours**. **Stalk cells** proliferate until they fill the **sub**-**RPE** space in **month** 9, after which proliferation slows down. The 45 **stalk cells** form a connected capillary network in the **sub-RPE** space. **Cell** **type** colors: 1) **POS** and **PIS**: light purple, 2) **RPE**: brown, 3) **Stalk cells**: green, 4) **Vascular cells** (**CC**): red, 5) **BrM**: light blue. We have rendered the boundaries of individual cells as semi-transparent membranes. **POS**, **PIS** and **RPE** cells are more transparent to show the underlying structures.
(AVI)

Video S2 **Time-Series of a Simulation Replica Showing Sub-RPE to Sub-Retinal Translocation (T12 Translocation).** 3D visualization of a simulation replica exhibiting **T12 CNV** translocation during one simulated **year** (*RRl* = 3, *RRp* = 3, *RBl* = 1, *RBp* = 1, *ROl* = 1) (adhesion scenario ID: 93, simulation ID: 849). **Stalk cells** invade the **sub-RPE** space through a hole in **BrM** and form a capillary network. All **stalk cells** remain in the **sub-RPE** space during the first 3 **months**. A few **vascular cells** fill the hole in **BrM** to connect **CNV** capillaries to the **CC**. Half of the **stalk cells** have crossed the **RPE** and transmigrated into the **sub-retinal** space by **month** 5, forming a new capillary network in the **sub-retinal** space. Most **stalk cells** have transmigrated into the **sub-retinal** space and the **RPE** has completely reattached to **BrM** by **month** 9. A few **vascular cells** of the **CC** have transmigrated into the **sub-retinal** space. The **sub-retinal** capillary network has fewer **stalk cells** than the capillary in **month** 9 since **stalk cells** that migrate into the retina far from the **RPE** die. **Cell** **type** colors: 1) **POS** and **PIS**: light purple, 2) **RPE**: brown, 3) **Stalk cells**: green (**stalk cells** in the **sub-retinal** space have lighter shading), 4) **Vascular cells** (**CC**): red, 5) **BrM**: light blue. We have rendered the boundaries of individual cells as semi-transparent membranes. **POS**, **PIS** and **RPE** cells are more transparent to show the underlying structures.
(AVI)

Video S3 **Time-Series of a Simulation Replica Showing Sub-RPE CNV to Sub-Retinal CNV Progression (P13 Progression).** 3D visualizations of a simulation replica exhibiting **P13 CNV** progression during one simulated **year** (*RRl* = 1, *RRp* = 3, *RBl* = 1, *RBp* = 2, *ROl* = 3) (adhesion scenario ID: 83, simulation ID: 515). **Stalk cells** invade the **sub-RPE** space through a hole in **BrM** and form a capillary network. The **vascular cells** of the **CC** occupy the hole that the **tip cell** forms during the first 24 **hours** of the simulation, connecting the **CNV** capillaries to the **CC**. All **stalk cells** remain in the **sub-RPE** space during the first **month** of the simulation. **Month** 2: A few **stalk cells** cross the **RPE** into the **sub-retinal** space. **Month** 6: Additional **stalk cells** migrate into the **sub-retinal** space and form vascular cords. The **stalk cells** form a **sub-RPE** capillary network connected to a **sub-retinal** capillary network. **Cell type** colors: 1) **POS** and **PIS**: light purple, 2) **RPE**: brown, 3) **Stalk cells**: green (**stalk cells** in the **sub-retinal** space have lighter shading), 4) **Vascular cells** (**CC**): red, 5) **BrM**: light blue. We have rendered the boundaries of individual cells as semi-transparent membranes. **POS**, **PIS** and **RPE** cells are more transparent to show the underlying structures.
(AVI)

Video S4 **Time-Series of a Simulation Replica Showing Stable Type CNV (S22 CNV).** 3D visualization of a simulation replica showing **S22 CNV** in one simulated **year** (*RRl* = 1, *RRp* = 1, *RBl* = 3, *RBp* = 3, *ROl* = 3) (adhesion scenario ID: 16, simulation ID: 556). **Stalk cells** invade the **sub-retinal** space through a hole in **BrM** and form a partially developed capillary network by **month** 2. **CNV** finishes **sub-retinal** invasion around **month** 5 and remains in the **sub-retinal** space throughout **LT2 CNV**. **Cell type** colors: 1) **POS** and **PIS**: light purple, 2) **RPE**: brown (**stalk cells** in the **sub-retinal** space have lighter shading), 3) **Stalk cells**: green, 4) **Vascular cells** (**CC**): red, 5) **BrM**: light blue. We have rendered the boundaries of individual cells as semi-transparent membranes. **POS**, **PIS** and **RPE** cells are more transparent to show the underlying structures.
(AVI)

Video S5 **Time-Series of a Simulation Replica Exhibiting Sub-Retinal CNV to Sub-RPE CNV Progression (P23 CNV).** 3D visualization of a simulation replica forming **P23 CNV** in one simulated **year** (*RRl* = 1, *RRp* = 1, *RBl* = 1, *RBp* = 1, *ROl* = 1) (adhesion scenario ID: 108, simulation ID: 1080). **Stalk cells** invade the **sub-retinal** space through the hole in **BrM** that the **tip cell** form during the first 24 **hours** of the simulation and form a fully developed **sub-retinal** capillary network by **month** 1. Only a few **stalk cells**, mostly near the hole in **BrM**, invade the **sub-RPE** space during the first **month**. Additional **stalk cells** invade the **sub-RPE** space by **month** 6, disrupting the **RPE** and causing a micro-tear. The **POS** contacts **BrM** at the location of the **RPE** tear. **Cell type** colors: 1) **POS** and **PIS**: light purple, 2) **RPE**: brown, 3) **Stalk cells**: green (**stalk cells** in the **sub-retinal** space have lighter shading), 4) **Vascular cells** (**CC**): red, 5) **BrM**: light blue. We have rendered the boundaries of individual cells as semi-transparent membranes. **POS**, **PIS** and **RPE** cells are more transparent to show the underlying structures.
(AVI)

Video S6 **Time-Series of a Simulation Replica Exhibiting Stable Type 3 CNV (S33 CNV).** 3D visualization of a simulation replica developing **S33 CNV** in one simulated **year** (*RRl* = 1, *RRp* = 1, *RBl* = 2, *RBp* = 2, *ROl* = 3) (adhesion scenario ID: 53, simulation ID: 917). **Stalk cells** invade the **sub-RPE** space through a hole in **BrM** that the **tip cell** form during the first 24 **hours** of the simulation. These **stalk cells** then form a fully developed **sub-RPE** capillary network by **month** 1. Only a few **stalk cells** reach the **sub-retinal** space during the first **month**. The **sub-retinal** and **sub-RPE** capillary networks do not grow significantly between **month** 1 and 2. **RPE cells** envelop **stalk cells**. **Stalk cells** disrupt the **RPE**, forming small holes in the **RPE**. **Cell type** colors: 1) **POS** and **PIS**: light purple, 2) **RPE**: brown, 3) **Stalk cells**: green, 4) **Vascular cells** (**CC**): red, 5) **BrM**: light blue. We have rendered the boundaries of individual cells as semi-transparent membranes. **POS**, **PIS** and **RPE** cells are more transparent to show the underlying structures.
(AVI)
