# Supplementary material for: Adhesion Failures Determine the Pattern of Choroidal Neovascularization in the Eye: A Computer Simulation Study
Source: PLoS Comput Biol. 2012 May 3;8(5):e1002440. doi: 10.1371/journal.pcbi.1002440 (PMC3342931; doi:10.1371/journal.pcbi.1002440)
Supplement: Table S1 — Adhesion Scenarios with Infrequent or No CNV Initiation. (PDF) [file pcbi.1002440.s001.pdf]

ID	*RRl*	*RRp*	*RBl*	*RBp*	*ROl*	P_{init}
1	3	3	3	3	3	0.00
4	3	2	3	3	3	0.20
2	3	3	3	2	3	0.30
3	3	3	3	1	3	0.30
7	3	1	3	3	3	0.30

Table S1. **Adhesion Scenarios with Infrequent or No CNV Initiation.** Adhesion scenarios that result in **CNV** initiation with probability less than or equal to 0.3 ($P_{init} \leq 0.3$). **CNV** does not initiate when adhesion is normal. Moderate to severe impairment of either **RPE-RPE** or **RPE-BrM plastic coupling** alone, without impairment of the corresponding **labile adhesion** barely increases the probabilities of **CNV** initiation. Key: ID: adhesion scenario ID. *RRl*: **RPE-RPE labile adhesion** strength, *RRp*: **RPE-RPE plastic coupling** strength, *RBl*: **RPE-BrM labile adhesion** strength, *RBp*: **RPE-BrM plastic coupling** strength, *ROl*: **RPE-POS labile adhesion** strength. P_{init}: **CNV** initiation probability calculated from 10 simulation replicas for each adhesion scenario. Scaled adhesion strengths: 3: normal (green), 2: moderately impaired (yellow), 1: severely impaired (weak) (red).
