# Supplementary material for: Adhesion Failures Determine the Pattern of Choroidal Neovascularization in the Eye: A Computer Simulation Study
Source: PLoS Comput Biol. 2012 May 3;8(5):e1002440. doi: 10.1371/journal.pcbi.1002440 (PMC3342931; doi:10.1371/journal.pcbi.1002440)
Supplement: Table S2 — Adhesion Scenarios Prone to Early Type 1 CNV (MW>0.9) if CNV Initiates. (PDF) [file pcbi.1002440.s002.pdf]

ID	*RRl*	*RRp*	*RBl*	*RBp*	*ROl*	P_{init}	<*MW*>
3	3	3	3	1	3	0.30	1.00
38	3	3	2	2	3	0.90	1.00
39	3	3	2	1	3	1.00	1.00
73	3	3	1	3	3	1.00	1.00
74	3	3	1	2	3	1.00	1.00
75	3	3	1	1	3	1.00	1.00
41	3	2	2	2	3	1.00	0.99
76	3	2	1	3	3	1.00	1.00
77	3	2	1	2	3	1.00	1.00
78	3	2	1	1	3	1.00	1.00
79	3	1	1	3	3	1.00	1.00
80	3	1	1	2	3	1.00	1.00
81	3	1	1	1	3	1.00	1.00
83	1	3	1	2	3	1.00	0.90
84	1	3	1	1	3	1.00	0.93

Table S2. **Adhesion Scenarios Prone to Early Type 1 CNV (*MW* > 0.9) if CNV Initiates.** A large *MW* indicates that almost no **stalk cells** cross the **RPE** and come into contact with the **POS**. **Early Type 1 CNV** occurs primarily for two main classes of adhesion scenarios: 1) When **RPE-BrM labile adhesion** is moderately to severely impaired ($RBl + RBp \leq 4$), but **RPE-RPE** and **RPE-POS labile adhesion** are both normal ($RRl = 3$ and $ROl = 3$). 2) When both **RPE-RPE** and **RPE-BrM labile adhesion** are severely impaired ($RRl = 1$ and $RBl = 1$), **RPE-BrM plastic coupling** strength is moderately to severely impaired ($RBp \leq 2$) and both **RPE-RPE plastic coupling** adhesion and **RPE-POS labile adhesion** are normal ($RRp = 3$, $ROl = 3$) (ID: 83 and 84). The **CNV** initiation probability ranges from 0.3 to 1. Key: ID: adhesion scenario ID. *RRl*: **RPE-RPE labile adhesion** strength, *RRp*: **RPE-RPE plastic coupling** strength, *RBl*: **RPE-BrM labile adhesion** strength, *RBp*: **RPE-BrM plastic coupling** strength, *ROl*: **RPE-POS labile adhesion** strength. P_{init}: **CNV** initiation probability. <*MW*>: mean morphometric weight. Both <*MW*> and P_{init} are calculated from 10 simulation replicas for each adhesion scenario. Scaled adhesion strengths: 3: normal (green), 2: moderately impaired (yellow), 1: severely impaired (weak) (red). Adhesion scenarios sequentially sorted largest to smallest in order by *RRl*, then by *RRp*, then by *RBl*, then by *RBp* and then by *ROl*.
