# Supplementary material for: Adhesion Failures Determine the Pattern of Choroidal Neovascularization in the Eye: A Computer Simulation Study
Source: PLoS Comput Biol. 2012 May 3;8(5):e1002440. doi: 10.1371/journal.pcbi.1002440 (PMC3342931; doi:10.1371/journal.pcbi.1002440)
Supplement: Table S3 — Adhesion Scenarios Prone to Early Type 2 CNV (MW<0.05) if CNV Initiates. (PDF) [file pcbi.1002440.s003.pdf]

ID	*RRl*	*RRp*	*RBl*	*RBp*	*ROl*	P_{init}	<*MW*>
19	3	3	3	3	1	0.50	0.04
20	3	3	3	2	1	0.80	0.01
21	3	3	3	1	1	0.90	0.01
55	3	3	2	3	1	0.50	0.03
22	3	2	3	3	1	0.80	0.02
23	3	2	3	2	1	1.00	0.01
24	3	2	3	1	1	1.00	0.02
58	3	2	2	3	1	0.90	0.02
60	3	2	2	1	1	1.00	0.04
25	3	1	3	3	1	0.90	0.01
26	3	1	3	2	1	0.90	0.02
27	3	1	3	1	1	1.00	0.01
61	3	1	2	3	1	1.00	0.01
62	3	1	2	2	1	1.00	0.03
10	1	3	3	3	3	1.00	0.02
28	1	3	3	3	1	1.00	0.00
11	1	3	3	2	3	1.00	0.03
29	1	3	3	2	1	1.00	0.01
12	1	3	3	1	3	1.00	0.03
30	1	3	3	1	1	1.00	0.01
64	1	3	2	3	1	1.00	0.01
65	1	3	2	2	1	1.00	0.01
66	1	3	2	1	1	1.00	0.01
100	1	3	1	3	1	1.00	0.02
101	1	3	1	2	1	1.00	0.05
13	1	2	3	3	3	1.00	0.03
31	1	2	3	3	1	1.00	0.02
14	1	2	3	2	3	1.00	0.03
32	1	2	3	2	1	1.00	0.03
15	1	2	3	1	3	1.00	0.05
33	1	2	3	1	1	1.00	0.03
67	1	2	2	3	1	1.00	0.03
68	1	2	2	2	1	1.00	0.04
69	1	2	2	1	1	1.00	0.04
103	1	2	1	3	1	1.00	0.04
16	1	1	3	3	3	1.00	0.03
34	1	1	3	3	1	1.00	0.02
17	1	1	3	2	3	1.00	0.03
35	1	1	3	2	1	1.00	0.03
36	1	1	3	1	1	1.00	0.03
70	1	1	2	3	1	1.00	0.03
72	1	1	2	1	1	1.00	0.05

106	1	1	1	3	1	1.00	0.05

Table S3. **Adhesion Scenarios Prone to Early Type 2 CNV (*MW* < 0.05) if CNV Initiates.** A small *MW* indicates that most **stalk cells** cross the **RPE** and come into contact with the **POS**. **ET2 CNV** occurs primarily for three main classes of adhesion scenarios: 1) When **RPE-RPE labile adhesion** is normal (*RRl* = 3), **RPE-BrM labile adhesion** is normal or moderately impaired (*RBl* ≥ 2), and **RPE-POS labile adhesion** is severely impaired (*ROl* = 1). 2) When **RPE-RPE labile adhesion** is severely impaired (*RRl* = 1) and **RPE-BrM labile adhesion** is either normal or moderately impaired (*RBl* ≥ 2). 3) When **RPE-RPE**, **RPE-BrM** and **RPE-POS labile adhesion** are severely impaired (*RRl* = *RBl* = *ROl* = 1), and the combination of **RPE-RPE** and **RPE-BrM plastic coupling** satisfies *RBp* + *RRp* > 3. Unless all **labile adhesions** are severely impaired, impairment of either **RPE-RPE** or **RPE-BrM plastic coupling** has little effect on the average *MW*, though it does increase the **CNV** initiation probability. For example, adhesion scenarios ID: 22 and 24, which differ only in their **RPE-BrM plastic coupling**, exhibit the same mean *MW*; however, P_{init} = 0.8 for normal **RPE-BrM plastic coupling** (ID: 22) and P_{init} = 1 for severely impaired **RPE-BrM plastic coupling** (ID: 24). The **CNV** initiation probability ranges from 0.5 to 1. Key: ID: adhesion scenario ID. *RRl*: **RPE-RPE labile adhesion** strength, *RRp*: **RPE-RPE plastic coupling** strength, *RBl*: **RPE-BrM labile adhesion** strength, *RBp*: **RPE-BrM plastic coupling** strength, *ROl*: **RPE-POS labile adhesion** strength. P_{init}: **CNV** initiation probability. <*MW*>: mean morphometric weight. Both <*MW*> and P_{init} calculated from 10 simulation replicas for each adhesion scenario. Scaled adhesion strengths: 3: normal (green), 2: moderately impaired (yellow), 1: severely impaired (weak) (red). Adhesion scenarios sequentially sorted largest to smallest in order by *RRl*, then by *RRp*, then by *RBl*, then by *RBp* and then by *ROl*.
