# Supplementary material for: Adhesion Failures Determine the Pattern of Choroidal Neovascularization in the Eye: A Computer Simulation Study
Source: PLoS Comput Biol. 2012 May 3;8(5):e1002440. doi: 10.1371/journal.pcbi.1002440 (PMC3342931; doi:10.1371/journal.pcbi.1002440)
Supplement: Table S4 — Adhesion Scenarios Prone to Early Type 3 CNV (0.35<MW<0.65) if CNV initiates. (PDF) [file pcbi.1002440.s004.pdf]

ID	*RRl*	*RRp*	*RBl*	*RBp*	*ROl*	P_{init}	<*MW*>
47	1	3	2	2	3	1.00	0.39
48	1	3	2	1	3	1.00	0.49
82	1	3	1	3	3	1.00	0.44
51	1	2	2	1	3	1.00	0.43
85	1	2	1	3	3	1.00	0.44
54	1	1	2	1	3	1.00	0.40
88	1	1	1	3	3	1.00	0.45

Table S4. **Adhesion Scenarios Prone to Early Type 3 CNV (0.35 < *MW* < 0.65) if CNV initiates.** 0.35 < *MW* < 0.65 indicates that **stalk cells** come into contact with both **BrM** and the **POS**. Most adhesion scenarios have severely impaired **RPE-RPE labile adhesion**, normal **RPE-POS labile adhesion** and either severely or moderately impaired **RPE-BrM labile adhesion** ($RBl \leq 2$). **RPE-RPE** and **RPE-BrM plastic coupling** have little effect on the mean *MW* and **CNV** initiation probability. For example, adhesion scenarios ID: 82 (*RRp* = 3), 85 (*RRp* = 2) and 88 (*RRp* = 1) have similar mean *MW* and **CNV** initiation probability regardless of the **RPE-RPE plastic coupling** strength (*RRp*). The **CNV** initiation probability ranges from 0.7 to 1. Key: ID: adhesion scenario ID. *RRl*: **RPE-RPE labile adhesion** strength, *RRp*: **RPE-RPE plastic coupling** strength, *RBl*: **RPE-BrM labile adhesion** strength, *RBp*: **RPE-BrM plastic coupling** strength, *ROl*: **RPE-POS labile adhesion** strength. P_{init}: **CNV** initiation probability. <*MW*>: mean morphometric weight. Both <*MW*> and P_{init} are calculated from 10 simulation replicas for each adhesion scenario. Scaled adhesion strengths: 3: normal (green), 2: moderately impaired (yellow), 1: severely impaired (weak) (red). Adhesion scenarios sequentially sorted largest to smallest in order by *RRl*, then by *RRp*, then by *RBl*, then by *RBp* and then by *ROl*.
