# Supplementary material for: Adhesion Failures Determine the Pattern of Choroidal Neovascularization in the Eye: A Computer Simulation Study
Source: PLoS Comput Biol. 2012 May 3;8(5):e1002440. doi: 10.1371/journal.pcbi.1002440 (PMC3342931; doi:10.1371/journal.pcbi.1002440)
Supplement: Table S5 — Adhesion Scenarios Prone to Stable Type 1 CNV (S11 CNV Probability>0.9). (PDF) [file pcbi.1002440.s005.pdf]

ID	*RRl*	*RRp*	*RBl*	*RBp*	*ROl*	P_{init}	**S11** Probability
3	3	3	3	1	3	0.30	1.00
41	3	2	2	2	3	1.00	1.00
80	3	1	1	2	3	1.00	0.90
79	3	1	1	3	3	1.00	0.90
77	3	2	1	2	3	1.00	0.90
76	3	2	1	3	3	1.00	0.90
74	3	3	1	2	3	1.00	0.90
73	3	3	1	3	3	1.00	0.90
39	3	3	2	1	3	1.00	0.90
38	3	3	2	2	3	0.90	0.90

Table S5. **Adhesion Scenarios Prone to Stable Type 1 CNV (S11 CNV Probability > 0.9)**. Adhesion scenarios that develop **Early Type 1 CNV** (**ET1 CNV**, *MW* > 75%) in which **CNV** remains confined to the **sub-RPE** space (**Late Type 1 CNV**, *MW* > 75%). **S11 CNV** occurs primarily when **RPE-BrM labile adhesion** is moderately to severely impaired, **RPE-BrM plastic coupling** satisfies $3 \leq RBl + RBp \leq 4$, and both **RPE-RPE** and **RPE-POS labile adhesion** are normal ($RRl = 3$ and $ROl = 3$). However, adhesion scenario ID: 3 belongs to a separate class. The **CNV** initiation probability ranges from 0.3 to 1. Key: ID: adhesion scenario ID. *RRl*: **RPE-RPE labile adhesion** strength, *RRp*: **RPE-RPE plastic coupling** strength, *RBl*: **RPE-BrM labile adhesion** strength, *RBp*: **RPE-BrM plastic coupling** strength, *ROl*: **RPE-POS labile adhesion** strength. P_{init}: **CNV** initiation probability. **S11** probability: Probability of occurrence **S11 CNV**. Both **S11** probability and P_{init} are calculated from 10 simulation replicas for each adhesion scenario. Scaled adhesion strengths: 3: normal (green), 2: moderately impaired (yellow), 1: severely impaired (weak) (red). Adhesion scenarios sequentially sorted largest to smallest in order by *RRl*, then by *RRp*, then by *RBl*, then by *RBp* and then by *ROl*.
