# Supplementary material for: Adhesion Failures Determine the Pattern of Choroidal Neovascularization in the Eye: A Computer Simulation Study
Source: PLoS Comput Biol. 2012 May 3;8(5):e1002440. doi: 10.1371/journal.pcbi.1002440 (PMC3342931; doi:10.1371/journal.pcbi.1002440)
Supplement: Table S6 — Adhesion Scenarios Prone to Sub-RPE to Sub-Retinal Translocation (T12 Translocation). (PDF) [file pcbi.1002440.s006.pdf]

ID	*RRl*	*RRp*	*RBl*	*RBp*	*ROl*	P_{init}	**T12** Probability
91	3	3	1	3	1	0.90	0.50
92	3	3	1	2	1	1.00	0.40
93	3	3	1	1	1	1.00	0.70
94	3	2	1	3	1	1.00	0.30
97	3	1	1	3	1	1.00	0.30

Table S6. **Adhesion Scenarios Prone to Sub-RPE to Sub-Retinal Translocation (T12 Translocation). T12** translocation occurs primarily when **RPE-RPE labile adhesion** is normal (*RRl* = 3), both **RPE-BrM** and **RPE-POS labile adhesion** are severely impaired (*RBl* = 1 and *ROl* = 1), and the combination of **RPE-BrM** and **RPE-POS plastic coupling** satisfies $RRp + RBp \geq 4$, except for the case of *RRp* = *RBp* = 2. Key: ID: adhesion scenario ID. *RRl*: **RPE-RPE labile adhesion** strength, *RRp*: **RPE-RPE plastic coupling** strength, *RBl*: **RPE-BrM labile adhesion** strength, *RBp*: **RPE-BrM plastic coupling** strength, *ROl*: **RPE-POS labile adhesion** strength. P_{init}: **CNV** initiation probability. **T12** probability: Probability of occurrence of **T12 CNV**. Both the **T12** probability and P_{init} are calculated from 10 simulation replicas for each adhesion scenario. Scaled adhesion strengths: 3: normal (green), 2: moderately impaired (yellow), 1: severely impaired (weak) (red). Adhesion scenarios sequentially sorted largest to smallest in order by *RRl*, then by *RRp*, then by *RBl*, then by *RBp* and then by *ROl*.
