# Supplementary material for: Adhesion Failures Determine the Pattern of Choroidal Neovascularization in the Eye: A Computer Simulation Study
Source: PLoS Comput Biol. 2012 May 3;8(5):e1002440. doi: 10.1371/journal.pcbi.1002440 (PMC3342931; doi:10.1371/journal.pcbi.1002440)
Supplement: Table S7 — Selected Adhesion Scenarios Prone to Sub-RPE to Sub-Retinal Progression (P13 Progression) (P13 Probability>0.7). (PDF) [file pcbi.1002440.s007.pdf]

| ID | *RRl* | *RRp* | *RBl* | *RBp* | *ROl* | $P_{\text{init}}$ | **P13** Probability |
|---|---|---|---|---|---|---|---|
| 83 | 1 | 3 | 1 | 2 | 3 | 1.00 | 1.00 |
| 86 | 1 | 2 | 1 | 2 | 3 | 1.00 | 1.00 |
| 90 | 1 | 1 | 1 | 1 | 3 | 1.00 | 1.00 |
| 89 | 1 | 1 | 1 | 2 | 3 | 1.00 | 1.00 |

Table S7. **Selected Adhesion Scenarios Prone to Sub-RPE to Sub-Retinal Progression** (**P13 Progression**) **(P13 Probability > 0.7). P13** progression primarily occurs when both **RPE-RPE** and **RPE-BrM labile adhesion** are severely impaired ($RRl$ = 1 and $RBl$ = 1), **RPE-BrM plastic coupling** is moderately to severely impaired ($RBp \leq 2$), and **RPE-POS labile adhesion** is normal ($ROl$ = 3). Key: ID: adhesion scenario ID. *RRl*: **RPE-RPE labile adhesion** strength, *RRp*: **RPE-RPE plastic coupling** strength, *RBl*: **RPE-BrM labile adhesion** strength, *RBp*: **RPE-BrM plastic coupling** strength, *ROl*: **RPE-POS labile adhesion** strength. $P_{\text{init}}$: **CNV** initiation probability. **P13** probability: **P13 CNV** probability of occurrence. Both **P13** probability and $P_{\text{init}}$ are calculated from 10 simulation replicas for each adhesion scenario. Scaled adhesion strengths: 3: normal (green), 2: moderately impaired (yellow), 1: severely impaired (weak) (red). Adhesion scenarios sequentially sorted largest to smallest in order by *RRl*, then by *RRp*, then by *RBl*, then by *RBp* and then by *ROl*.
