# Supplementary material for: Adhesion Failures Determine the Pattern of Choroidal Neovascularization in the Eye: A Computer Simulation Study
Source: PLoS Comput Biol. 2012 May 3;8(5):e1002440. doi: 10.1371/journal.pcbi.1002440 (PMC3342931; doi:10.1371/journal.pcbi.1002440)
Supplement: Table S8 — Adhesion Scenarios Prone to Stable Type 2 CNV (S22) (S22 Probability>0.9). (PDF) [file pcbi.1002440.s008.pdf]

ID	*RRl*	*RRp*	*RBl*	*RBp*	*ROl*	P_{init}	**S22** Probability
21	3	3	3	1	1	0.90	0.90
23	3	2	3	2	1	1.00	1.00
24	3	2	3	1	1	1.00	1.00
58	3	2	2	3	1	0.90	0.90
60	3	2	2	1	1	1.00	1.00
25	3	1	3	3	1	0.90	0.90
26	3	1	3	2	1	0.90	0.90
27	3	1	3	1	1	1.00	1.00
61	3	1	2	3	1	1.00	1.00
62	3	1	2	2	1	1.00	1.00
63	3	1	2	1	1	1.00	1.00
10	1	3	3	3	3	1.00	1.00
28	1	3	3	3	1	1.00	1.00
11	1	3	3	2	3	1.00	1.00
29	1	3	3	2	1	1.00	1.00
12	1	3	3	1	3	1.00	1.00
30	1	3	3	1	1	1.00	1.00
46	1	3	2	3	3	1.00	1.00
64	1	3	2	3	1	1.00	1.00
65	1	3	2	2	1	1.00	1.00
66	1	3	2	1	1	1.00	1.00
100	1	3	1	3	1	1.00	1.00
101	1	3	1	2	1	1.00	1.00
102	1	3	1	1	1	1.00	1.00
13	1	2	3	3	3	1.00	1.00
31	1	2	3	3	1	1.00	1.00
14	1	2	3	2	3	1.00	1.00
32	1	2	3	2	1	1.00	1.00
15	1	2	3	1	3	1.00	1.00
49	1	2	2	3	3	1.00	1.00
67	1	2	2	3	1	1.00	1.00
68	1	2	2	2	1	1.00	1.00
103	1	2	1	3	1	1.00	1.00
104	1	2	1	2	1	1.00	0.90
16	1	1	3	3	3	1.00	1.00
34	1	1	3	3	1	1.00	1.00
17	1	1	3	2	3	1.00	1.00
35	1	1	3	2	1	1.00	1.00
52	1	1	2	3	3	1.00	1.00
70	1	1	2	3	1	1.00	1.00
71	1	1	2	2	1	1.00	0.90
106	1	1	1	3	1	1.00	1.00

Table S8. **Adhesion Scenarios Prone to Stable Type 2 CNV (S22) (S22 Probability > 0.9).** The three main classes of adhesion scenario that cause **ET2 CNV** also predominantly lead to **S22 CNV**: 1) When **RPE-RPE labile adhesion** is normal ($RRl = 3$), **RPE-BrM labile adhesion** is normal or moderately impaired ($RBl \geq 2$), and **RPE-POS labile adhesion** is severely impaired ($ROl = 1$). 2) When **RPE-RPE labile adhesion** is weak ($RRl = 1$), **RPE-BrM labile adhesion** is normal or moderately impaired ($RBl \geq 2$). 3) When **RPE-RPE**, **RPE-BrM** and **RPE-POS labile adhesion** are severely impaired ($RRl = RBl = ROl = 1$), and the combination of **RPE-RPE** and **RPE-BrM plastic coupling** strengths satisfies $RBp + RRp > 3$. Key: ID: adhesion scenario ID. *RRl*: **RPE-RPE labile adhesion** strength, *RRp*: **RPE-RPE plastic coupling** strength, *RBl*: **RPE-BrM labile adhesion** strength, *RBp*: **RPE-BrM plastic coupling** strength, *ROl*: **RPE-POS labile adhesion** strength. P_{init}: **CNV** initiation probability. Both the **S22 CNV** probability and P_{init} are calculated from 10 simulation replicas for each adhesion scenario. Scaled adhesion strengths: 3: normal (green), 2: moderately impaired (yellow), 1: severely impaired (weak) (red). Adhesion scenarios sequentially sorted largest to smallest in order by *RRl*, then by *RRp*, then by *RBl*, then by *RBp* and then by *ROl*.
