# Supplementary material for: Adhesion Failures Determine the Pattern of Choroidal Neovascularization in the Eye: A Computer Simulation Study
Source: PLoS Comput Biol. 2012 May 3;8(5):e1002440. doi: 10.1371/journal.pcbi.1002440 (PMC3342931; doi:10.1371/journal.pcbi.1002440)
Supplement: Table S9 — Adhesion Scenarios Prone to Sub-Retinal CNV to Sub-RPE CNV Progression (P23 CNV) (P23 Probability>0.6). (PDF) [file pcbi.1002440.s009.pdf]

ID	*RRl*	*RRp*	*RBl*	*RBp*	*ROl*	P_{init}	**P23** Probability
105	1	2	1	1	1	1.00	0.60
108	1	1	1	1	1	1.00	1.00

Table S9. **Adhesion Scenarios Prone to Sub-Retinal CNV to Sub-RPE CNV Progression (P23 CNV) (P23 Probability > 0.6). P23 CNV** primarily occurs when **RPE-RPE plastic coupling** is severely or moderately impaired ($RRp \leq 2$) and all other adhesions are severely impaired ($RRl = 1$, $RBl = 1$, $RBp = 1$, $ROl = 1$). Key: ID: adhesion scenario ID. *RRl*: **RPE-RPE labile adhesion** strength, *RRp*: **RPE-RPE plastic coupling** strength, *RBl*: **RPE-BrM labile adhesion** strength, *RBp*: **RPE-BrM plastic coupling** strength, *ROl*: **RPE-POS labile adhesion** strength. P_{init}: **CNV** initiation probability. Both **P23 CNV** probability and P_{init} are calculated from 10 simulation replicas for each adhesion scenario. Scaled adhesion strengths: 3: normal (green), 2: moderately impaired (yellow), 1: severely impaired (weak) (red).
