# Supplementary material for: Adhesion Failures Determine the Pattern of Choroidal Neovascularization in the Eye: A Computer Simulation Study
Source: PLoS Comput Biol. 2012 May 3;8(5):e1002440. doi: 10.1371/journal.pcbi.1002440 (PMC3342931; doi:10.1371/journal.pcbi.1002440)
Supplement: Table S10 — Adhesion Scenarios Prone to Stable Type 3 CNV (S33 CNV) (S33 Probability>0.9). (PDF) [file pcbi.1002440.s010.pdf]

ID	*RRl*	*RRp*	*RBl*	*RBp*	*ROl*	Initiation Probability	**S33** Probability
88	1	1	1	3	3	1.00	1.00
54	1	1	2	1	3	1.00	1.00
53	1	1	2	2	3	1.00	1.00
85	1	2	1	3	3	1.00	1.00
51	1	2	2	1	3	1.00	1.00
50	1	2	2	2	3	1.00	1.00
82	1	3	1	3	3	1.00	1.00
48	1	3	2	1	3	1.00	1.00
47	1	3	2	2	3	1.00	1.00

Table S10. **Adhesion Scenarios Prone to Stable Type 3 CNV (S33 CNV) (S33 Probability > 0.9)**. **S33 CNV** occurs primarily for two classes of adhesion scenarios: 1) When **RPE-RPE labile adhesion** is severely impaired (*RRl* = 1), **RPE-POS labile adhesion** is normal (*ROl* = 3), **RPE-BrM labile adhesion** is moderately impaired (*RBl* = 2) and **RPE-BrM plastic coupling** satisfies $RBl + RBp \leq 4$. 2) When **RPE-RPE labile adhesion** is severely impaired (*RRl* = 1), **RPE-POS labile adhesion** is normal (*ROl* = 3), **RPE-BrM labile adhesion** is severely impaired (*RBl* = 1) and **RPE-BrM plastic coupling** is normal (*RBp* = 3). **RPE-RPE plastic coupling** has no effect on the probability of **CNV** initiation or occurrence of **S33 CNV** in these scenarios. Key: ID: adhesion scenario ID. *RRl*: **RPE-RPE labile adhesion** strength, *RRp*: **RPE-RPE plastic coupling** strength, *RBl*: **RPE-BrM labile adhesion** strength, *RBp*: **RPE-BrM plastic coupling** strength, *ROl*: **RPE-POS labile adhesion** strength. P_{init}: **CNV** initiation probability. Both the **P23 CNV** probability and P_{init} are calculated from 10 simulation replicas for each adhesion scenario. Scaled adhesion strengths: 3: normal (green), 2: moderately impaired (yellow), 1: severely impaired (weak) (red). Adhesion scenarios sequentially sorted largest to smallest in order by *RRl*, then by *RRp*, then by *RBl*, then by *RBp* and then by *ROl*.
