# Supplementary material for: Adhesion Failures Determine the Pattern of Choroidal Neovascularization in the Eye: A Computer Simulation Study
Source: PLoS Comput Biol. 2012 May 3;8(5):e1002440. doi: 10.1371/journal.pcbi.1002440 (PMC3342931; doi:10.1371/journal.pcbi.1002440)
Supplement: Text S1 — Biological Components and Processes in CNV. (PDF) [file pcbi.1002440.s011.pdf]

Text S1

Biological Components and Processes in CNV

CNV involves the interaction of two fundamental structures, the retina with its supporting structures and the choriocapillaris. We briefly review the functional and structural properties of their components in the context of CNV.

Retina, RPE, Bruch's Membrane and CC

The retina consists of two concentric regions, the *inner retina* and the *outer retina* proper (Figure 1). Beneath the outer retina proper lies the retinal pigment epithelium (*RPE*), a monolayer of pigmented epithelial cells situated between the photoreceptors and the choroid. The RPE plays numerous roles in maintenance of the retina. Beneath the RPE, *Bruch's membrane* (*BrM*), a strong, multi-layered, 2-to-4-μm-thick porous membrane structurally stabilizes the RPE (Figure 1). The very dense choriocapillaris (or choroid) lies behind the outer retina, separated from it by BrM and the RPE.

The inner retina, adjacent to the vitreous humor, includes the inner retinal vasculature and layers of neural cells. The outer retina, adjacent to the RPE, includes the rod and cone photoreceptors. Each rod or cone cell has three regions along its axis in the direction perpendicular to the retinal layers: the *cell body*, the *photoreceptor outer segment* (*POS*), which contains the light-absorbing outer-segment disks, and the *photoreceptor inner segment* (*PIS*) (Figure 1). The ensemble of POSs forms a well-defined layer in the retinal plane. Similarly, the ensemble of photoreceptor cell bodies forms the outer nuclear layer (*ONL*) separated from the PIS layer (the ensemble of PISs) by a membrane called the *outer* (or *external*) *limiting membrane* (*OLM*). The OLM supports and orients the cells which cross it and impedes the extracellular diffusion of large molecules [1].

The properties of the retinal layers vary depending on the in-layer distance from the *fovea*. The outer retina is thinner in the *periphery* far from the fovea and thicker in the fovea, where the density of photoreceptors is higher and vision is most acute. CNV is most damaging when it occurs in the subfoveal area. In the central fovea, the inner retina is thinnest and lacks inner-retinal vasculature [2,3].

The thickness, porosity, elasticity and composition of BrM all change due to aging [4] (see [5], for a comprehensive review). Extracellular material (mostly waste byproducts from phagocytosis) accumulates between the RPE and BrM forming *basal deposits*. Basal deposits located between the RPE plasma membrane and its basement membrane (*RBaM*) are called *basal laminar deposits* (*BlamD*). Basal deposits external to the RBaM in the *inner collagenous layer* or *inner collagenous zone* are called *basal linear deposits* (*BlinD*). Deposits that appear clinically as yellowish-white spots in the retina are called *drusen*. *Hard drusen* are mechanically stiff nodular deposits with defined edges and do not strongly correlate with CNV [6]. *Soft drusen* are mechanically softer, have less defined sloping edges and correlate strongly with CNV.

CNV is usually limited to the sub-RPE space (*i.e.* between the RPE and BrM) and sub-retinal space (*i.e.* between the RPE and photoreceptors), though anastomosis of CNV capillaries with the inner-retinal vasculature occasionally occurs [7].

Oxygen Transport and Metabolism in the Retina

Two capillary beds supply oxygen and nutrients to most regions of the retina and remove waste products, the inner-retinal capillaries and the choriocapillaris (*CC*). The CC supplies more than 90 percent of the oxygen to the photoreceptors in dark-adapted conditions and almost 100 percent in light-adapted conditions [8]. The inner retinal capillaries supply oxygen to the inner layers of the retina, maintaining the oxygen partial pressure (PO_2) almost constant in both light and dark-adapted conditions [8,9]. The normal oxygen concentration at the OLM varies slightly, depending on the in-layer distance from the fovea.

The PIS is packed with mitochondria, so photoreceptors have the highest oxygen consumption rates of any cells in the human body. The metabolic activity and oxygen concentration of the photoreceptors depend on the intensity of light they receive. In dark-adapted conditions, photoreceptors consume oxygen at about twice their light-adapted rates.

Adhesion Properties of the RPE, POS and PIS

The lipid bilayer forming the cell membrane is flimsy and cannot, by itself, transmit large forces from cell to cell or from cell to *extracellular matrix* (*ECM*). *Anchoring junctions* solve this problem by forming strong membrane-spanning structures that tether inside the cell to the tension-bearing filaments of the cytoskeleton.

RPE cells are apicobasally polarized. On the lateral surfaces of RPE cells, two *bands of epithelial adhesion junctions* connect to neighboring RPE cells (Figure 2). On the apical- most lateral surfaces of RPE cells, a band of *tight junctions* (*TJ*s) (z*onula occludens*, *ZO*, *Mesh*) seal adjacent RPE cells together, forming the outer blood-retina (*oBRB*) barrier and restricting transport of material (*e.g.* albumin) into and out of the retina [10-12]. On the lateral surfaces of RPE cells, basal to the TJs, *adherens junctions* (*AJ*s) form another junctional band that goes all the way around each cell and mechanically connects the cytoskeleton of each RPE cell to the neighboring RPE cells, giving structural integrity to the RPE. RPE cells form AJs predominantly via N-cadherins [13,14] (RPE cells also produce a small amount of E-cadherin, the most common adhesion molecule in the AJs of most other epithelial tissues). In addition to these junctional bands, *desmosome plaques* (*DP*s) and *gap junctions* distributed on the lateral surfaces of the RPE cells connect neighboring RPE cells. Desmosomes are crucial for tissue integrity, resist calcium-depletion in developed tissue and help to resist shearing forces. When we refer to e*pithelial junctions* without further qualification, we refer to the ensemble of junctional structures that participate in RPE-RPE adhesion, including TJs, AJs and DPs. The basal surfaces of RPE cells adhere to the very thin *basal laminae* of the RBaMs so strongly that the basal laminae behave like extensions of the cells' plasma membranes [15]. *Intergrins* mediate RPE-basal lamina adhesion. The RBaMs attach to the inner collagenous layer of BrM via microfibrils passing through both the elastic layer of BrM and the RBaMs [15,16]. When we refer to the *RPE-BrM complex*, we refer to the RPE-RBaM-BrM ensemble. Soft drusen reduce the adhesion

between the RBaMs and the inner collagenous zone (Figure 1) of BrM and correlate with localized detachment of the RPE from BrM [6,17-19]. Age-related modifications of BrM, especially soft drusen, also inhibit reattachment of transplanted RPE cells to BrM [20,21].

Photoreceptors pass spent photo-sensitive disks to the apical processes of RPE cells. This apical contact attaches photoreceptors to the RPE [22-24] more weakly than would RPE-RPE epithelial adhesion or attachment of RBaMs to BrM, so detachment of photoreceptors from the RPE (*retinal detachment*) due to impact is more likely than *RPE tears* (which break RPE-RPE epithelial junctions) or *RPE detachment* (which breaks RBaM-BrM attachment). Disruptions of RPE-POS contact affect not only the integrity of the oBRB, they also induce pathological cell growth and division in the RPE [25-27], disrupting the RPE epithelial structure and preventing successful therapeutic retinal reattachment (see Table 1).

Photoreceptors have limited or no motility and are held together in constant positions by multiple ECM components in the outer retina and OLM, ensuring consistant positional mapping of the visual field to the photoreceptors and the corresponding neurons in the visual cortex (*somatotopic mapping*). This somatotopic consistency is crucial to the development and maintenance of high-resolution visual perception.

Angiogenic and Antiangiogenic factors

Since laterally adjacent RPE cells form tight junctions, factors secreted by the photoreceptors on the apical side of the RPE do not pass through an intact RPE epithelial sheet to affect the choriocapillaris or CNV capillaries. The RPE secretes two VEGF-A isoforms from its basolateral surfaces to maintain the CC. VEGF-A_{120} diffuses freely and does not bind to heparin-sulfate. More than 75% of RPE-derived VEGF is the VEGF-A_{165} isoform [28] which has a weak affinity for heparin-sulfate, allowing it to diffuse across BrM while remaining resident long enough to bind to the VEGF receptors of the CC (otherwise the constant fluid flow from the vitreous humor to the CC and the high rate of CC blood flow would elute the VEGF before it bound to the EC's VEGF receptors). Mutant mice producing only VEGF-A_{188}, which binds strongly to extracellular matrix and therefore has a short diffusion length, develop a normal CC but suffer CC atrophy and RPE and BrM abnormalities, leading to RPE loss and dramatic choroidal remodeling beginning at 7 months [29], suggesting that RPE-derived free-diffusing VEGF-A isoforms are necessary to maintain the choriocapillaris. These VEGF isoforms may also help support other retinal cell types. When we refer to VEGF-A without qualifications, we mean VEGF-A_{165}, which appears to play the dominant role in CNV. In addition to RPE-derived VEGF-A, ECs, in general, produce multiple isoforms of VEGF-A, among which short-diffusing isoforms can serve as autocrine chemoattractants, playing a key role in capillary patterning (for a detailed discussion of capillary patterning mechanisms, see [30,31]). In many cases, ECs can only sense ECM-bound isoforms of VEGF-A when they are released from the ECM by matrix-degrading enzymes.

Since the retina is the most metabolically active tissue in the body, the density of capillaries in the CC is unusually high. The CC has small inter-capillary distances (~ 20 μm) [32,33] compare to typical inter-capillary distances (~ 100 μm to 200 μm). Since RPE-derived VEGF is essential for to maintain this dense population of ECs in the CC, we hypothesize that RPE-derived VEGF secretion must be substantial. The denser population of ECs consuming RPE-derived VEGF

globally balances the higher secretion rate of RPE-derived VEGF. However, because of inhomogeneities in the CC-BrM-RPE complex [16], we hypothesize that RPE-derived VEGF does not diffuse uniformly, producing relatively high local concentrations of RPE-derived VEGF, sufficient to maintain a substantial population of activated ECs in the CC even in the healthy retina.

RPE cells also produce the antiangiogenic *pigment-epithelium-derived factor* (*PEDF*). At homeostasis, proangiogenic and antiangiogenic factors balance. In the aged human retina, PEDF has a spatial distribution similar to that of VEGF-A [34].

Numerous other diffusible pro and antiangiogenic factors [35,36] may help modulate capillary behavior, but we do not consider them in this paper (see also the *Inflammation* subsection below). While angiogenesis requires proangiogenic factors to dominate antiangiogenic factors, normal angiogenesis requires the factors to remain in rough balance. In pathological situations, when the levels of proangiogenic factors are too large relative to antiangiogenic factors, the resulting vessels do not mature and remain leaky and insufficient at oxygen and nutrient transport.

Angiogenesis and BrM Degradation

High levels of VEGF-A activate normally quiescent endothelial cells in blood vessels. Via a Delta/Notch contact-inhibition selection mechanism, small populations of these activated ECs become *tip cells* which lead angiogenic *sprouts* up gradients of VEGF-A [37,38]. A morphologically distinct population of activated ECs called *stalk* cells form the body of these angiogenic sprouts. While tip and stalk cells are distinct at any instant, they can dynamically exchange identities [39]. Macrophages and other immune cells can substitute for tip cells in their pathfinding role. Tip cells have very low rates of proliferation, while stalk cells proliferate at moderate rates.

Excess proteolytic activity of macrophages and activated endothelial cells can cause focal thinning of BrM [40-44]. Tip cells and immune cells express a number of matrix degrading enzymes which locally break down ECM and BrM, while BrM is continuously reformed by poorly understood means. RPE cells also secrete numerous inactive MMPs and tissue inhibitor of mellapoteinases (*TIMP*s) that inhibit activated MMPs [45-49]; *E.g.*, tip cells and macrophages express transmembrane type-1 MMP (*MT1-MMP*) which activates MMP-2, which plays a key role in tip-cell and macrophage migration and breakdown of basement membrane in tumor invasion [50-54]. A similar mechanism is plausible for tip-cell and immune-cell breakdown of BrM.

Inflammation

Inflammation and immune cells play major roles in CNV. Macrophages in normal retina remove debris accumulated in BrM [55], helping to maintain normal retinal structure and function. However, chronic or excessive acute inflammation can promote CNV initiation and impair the integrity of the RPE, promoting CNV progression. Irregularities in regulation of the complement cascade and overactivity of immune cells may perturb RPE cells, causing them to form more

basal deposits (both soft and hard drusen), which in turn induce a stronger immune response which can initiate angiogenesis [56,57]. *Angiopoietin-2*, a key proangiogenic inflammatory factor, activates quiescent ECs with a response modulated by local VEGF concentrations and increases ECs' directional motility. *Angiopoietin-1*, on the other hand, inhibits activation of ECs and helps newly-formed vessels mature [58]. Pro-inflammatory cytokines, *e.g.*, tumor necrosis factor alpha (*TNF-α*) and *interleukin-1β* and *-8*, result in extensive breakdown of both the oBRB and inner blood-retinal barrier (*iBRB*) which separates inner-retinal capillaries from the outer retina [12,59-62].

Inflammation may affect early and late-stage CNV by weakening RPE-RPE and RPE-POS adhesion. Inflammation also impairs the juxtacrine Delta/Notch inhibitory signaling which couples adjacent ECs and normally promotes ECs quiescence and inhibits tip-cell selection, increasing EC activation [63].

References

1. Bunt-Milam AH, Saari JC, Klock IB, Garwin GG (1985) *Zonulae adherentes* pore size in the external limiting membrane of the rabbit retina. Invest Ophthalmol Vis Sci 26: 1377-1380.
2. Cheng H, Nair G, Walker TA, Kim MK, Pardue MT, et al. (2006) Structural and functional MRI reveals multiple retinal layers. Proc Natl Acad Sci USA 103: 17525-17530.
3. Bagci AM, Shahidi M, Ansari R, Blair M, Blair NP, et al. (2008) Thickness profiles of retinal layers by optical coherence tomography image segmentation. Am J Ophthalmol 146: 679-687.
4. Ramrattan RS, van der Schaft TL, Mooy CM, de Bruijn WC, Mulder PG, et al. (1994) Morphometric analysis of Bruch's membrane, the choriocapillaris, and the choroid in aging. Invest Ophthalmol Vis Sci 35: 2857-2864.
5. Booij JC, Baas DC, Beisekeeva J, Gorgels TG, Bergen AA (2010) The dynamic nature of Bruch's membrane. Prog Retin Eye Res 29: 1-18.
6. Sarks SH, Arnold JJ, Killingsworth MC, Sarks JP (1999) Early drusen formation in the normal and aging eye and their relation to age related maculopathy: a clinicopathological study. Br J Ophthalmol 83: 358-368.
7. Slakter JS, Yannuzzi LA, Schneider U, Sorenson JA, Ciardella A, et al. (2000) Retinal choroidal anastomoses and occult choroidal neovascularization in age-related macular degeneration. Ophthalmology 107: 742-753; discussion 753-744.
8. Wangsa-Wirawan ND, Linsenmeier RA (2003) Retinal oxygen: fundamental and clinical aspects. Arch Ophthalmol 121: 547-557.
9. Linsenmeier RA (1986) Effects of light and darkness on oxygen distribution and consumption in the cat retina. J Gen Physiol 88: 521-542.
10. Simo R, Villarroel M, Corraliza L, Hernandez C, Garcia-Ramirez M (2010) The retinal pigment epithelium: something more than a constituent of the blood-retinal barrier. J Biomed Biotechnol 2010: 190724.
11. Marmor MF (1999) Mechanisms of fluid accumulation in retinal edema. Doc Ophthalmol 97: 239-249.

12. Kaur C, Foulds WS, Ling EA (2008) Blood-retinal barrier in hypoxic ischaemic conditions: basic concepts, clinical features and management. Prog Retin Eye Res 27: 622-647.
13. Burke JM (2008) Epithelial phenotype and the RPE: is the answer blowing in the Wnt? Prog Retin Eye Res 27: 579-595.
14. McKay BS, Irving PE, Skumatz CM, Burke JM (1997) Cell-cell adhesion molecules and the development of an epithelial phenotype in cultured human retinal pigment epithelial cells. Exp Eye Res 65: 661-671.
15. Goldbaum MH, Madden K (1982) A new perspective on Bruch's membrane and the retinal pigment epithelium. Br J Ophthalmol 66: 17-25.
16. Essner E, Gordon SR (1984) Demonstration of microfibrils in Bruch's membrane of the eye. Tissue Cell 16: 779-788.
17. Sarks JP, Sarks SH, Killingsworth MC (1994) Evolution of soft drusen in age-related macular degeneration. Eye (Lond) 8: 269-283.
18. Sarks SH, Van Driel D, Maxwell L, Killingsworth M (1980) Softening of drusen and subretinal neovascularization. Trans Ophthalmol Soc UK 100: 414-422.
19. Spraul CW, Grossniklaus HE (1997) Characteristics of drusen and Bruch's membrane in postmortem eyes with age-related macular degeneration. Arch Ophthalmol 115: 267-273.
20. Gullapalli VK, Sugino IK, Van Patten Y, Shah S, Zarbin MA (2005) Impaired RPE survival on aged submacular human Bruch's membrane. Exp Eye Res 80: 235-248.
21. Tezel TH, Del Priore LV, Kaplan HJ (2004) Reengineering of aged Bruch's membrane to enhance retinal pigment epithelium repopulation. Invest Ophthalmol Vis Sci 45: 3337-3348.
22. Defoe DM, Matsumoto B, Besharse JC (1992) Reconstitution of the photoreceptor-pigment epithelium interface: L-glutamate stimulation of adhesive interactions and rod disc shedding after recombination of dissociated *Xenopus laevis* eyecups. Exp Eye Res 54: 903-911.
23. Matsumoto B, Defoe DM, Besharse JC (1987) Membrane turnover in rod photoreceptors: ensheathment and phagocytosis of outer segment distal tips by pseudopodia of the retinal pigment epithelium. Proc R Soc Lond B Biol Sci 230: 339-354.
24. Gundersen D, Powell SK, Rodriguez-Boulan E (1993) Apical polarization of N-CAM in retinal pigment epithelium is dependent on contact with the neural retina. J Cell Biol 121: 335-343.
25. Johnson NF, Foulds WS (1977) Observations on the retinal pigment epithelium and retinal macrophages in experimental retinal detachment. Br J Ophthalmol 61: 564-572.
26. Anderson DH, Stern WH, Fisher SK, Erickson PA, Borgula GA (1983) Retinal detachment in the cat: the pigment epithelial-photoreceptor interface. Invest Ophthalmol Vis Sci 24: 906-926.
27. Anderson DH, Stern WH, Fisher SK, Erickson PA, Borgula GA (1981) The onset of pigment epithelial proliferation after retinal detachment. Invest Ophthalmol Vis Sci 21: 10-16.
28. Saint-Geniez M, Maldonado AE, D'Amore PA (2006) VEGF expression and receptor activation in the choroid during development and in the adult. Invest Ophthalmol Vis Sci 47: 3135-3142.
29. Saint-Geniez M, Kurihara T, Sekiyama E, Maldonado AE, D'Amore PA (2009) An essential role for RPE-derived soluble VEGF in the maintenance of the choriocapillaris. Proc Natl Acad Sci U S A 106: 18751-18756.

30. Merks RM, Perryn ED, Shirinifard A, Glazier JA (2008) Contact-inhibited chemotaxis in *de novo* and sprouting blood-vessel growth. PLoS Comput Biol 4: e1000163.
31. Shirinifard A, Gens JS, Zaitlen BL, Poplawski NJ, Swat M, et al. (2009) 3D multi-cell simulation of tumor growth and angiogenesis. PLoS One 4: e7190.
32. McLeod DS, Lutty GA (1994) High-resolution histologic analysis of the human choroidal vasculature. Invest Ophthalmol Vis Sci 35: 3799-3811.
33. Spraul CW, Lang GE, Grossniklaus HE, Lang GK (1999) Histologic and morphometric analysis of the choroid, Bruch's membrane, and retinal pigment epithelium in postmortem eyes with age-related macular degeneration and histologic examination of surgically excised choroidal neovascular membranes. Surv Ophthalmol 44: 10-32.
34. Bhutto IA, McLeod DS, Hasegawa T, Kim SY, Merges C, et al. (2006) Pigment epithelium-derived factor (PEDF) and vascular endothelial growth factor (VEGF) in aged human choroid and eyes with age-related macular degeneration. Exp Eye Res 82: 99-110.
35. Martin G, Schlunck G, Hansen LL, Agostini HT (2004) Differential expression of angioregulatory factors in normal and CNV-derived human retinal pigment epithelium. Graefes Arch Clin Exp Ophthalmol 242: 321-326.
36. Kliffen M, Sharma HS, Mooy CM, Kerkvliet S, de Jong PT (1997) Increased expression of angiogenic growth factors in age-related maculopathy. Br J Ophthalmol 81: 154-162.
37. De Smet F, Segura I, De Bock K, Hohensinner PJ, Carmeliet P (2009) Mechanisms of vessel branching: filopodia on endothelial tip cells lead the way. Arterioscler Thromb Vasc Biol 29: 639-649.
38. Holderfield MT, Hughes CC (2008) Crosstalk between vascular endothelial growth factor, notch, and transforming growth factor-beta in vascular morphogenesis. Circ Res 102: 637-652.
39. Jakobsson L, Franco CA, Bentley K, Collins RT, Ponsioen B, et al. (2010) Endothelial cells dynamically compete for the tip cell position during angiogenic sprouting. Nat Cell Biol 12: 943-953.
40. Heriot WJ, Henkind P, Bellhorn RW, Burns MS (1984) Choroidal neovascularization can digest Bruch's membrane. A prior break is not essential. Ophthalmology 91: 1603-1608.
41. Killingsworth MC, Sarks JP, Sarks SH (1990) Macrophages related to Bruch's membrane in age-related macular degeneration. Eye (Lond) 4: 613-621.
42. Chen H, Liu B, Lukas TJ, Neufeld AH (2008) The aged retinal pigment epithelium/choroid: a potential substratum for the pathogenesis of age-related macular degeneration. PLoS One 3: e2339.
43. Cousins SW, Espinosa-Heidmann DG, Alexandridou A, Sall J, Dubovy S, et al. (2002) The role of aging, high fat diet and blue light exposure in an experimental mouse model for basal laminar deposit formation. Exp Eye Res 75: 543-553.
44. Penfold P, Killingsworth M, Sarks S (1984) An ultrastructural study of the role of leucocytes and fibroblasts in the breakdown of Bruch's membrane. Aust J Ophthalmol 12: 23-31.
45. Guo L, Hussain AA, Limb GA, Marshall J (1999) Age-dependent variation in metalloproteinase activity of isolated human Bruch's membrane and choroid. Invest Ophthalmol Vis Sci 40: 2676-2682.
46. Hoffmann S, He S, Ehren M, Ryan SJ, Wiedemann P, et al. (2006) MMP-2 and MMP-9 secretion by RPE is stimulated by angiogenic molecules found in choroidal neovascular membranes. Retina 26: 454-461.

47. Kvanta A, Shen WY, Sarman S, Seregard S, Steen B, et al. (2000) Matrix metalloproteinase (MMP) expression in experimental choroidal neovascularization. Curr Eye Res 21: 684-690.
48. Lambert V, Wielockx B, Munaut C, Galopin C, Jost M, et al. (2003) MMP-2 and MMP-9 synergize in promoting choroidal neovascularization. FASEB J 17: 2290-2292.
49. Ottino P, Finley J, Rojo E, Ottlecz A, Lambrou GN, et al. (2004) Hypoxia activates matrix metalloproteinase expression and the VEGF system in monkey choroid-retinal endothelial cells: Involvement of cytosolic phospholipase A2 activity. Mol Vis 10: 341-350.
50. Karagiannis ED, Popel AS (2006) Distinct modes of collagen type I proteolysis by matrix metalloproteinase (MMP) 2 and membrane type I MMP during the migration of a tip endothelial cell: insights from a computational model. J Theor Biol 238: 124-145.
51. Sato H, Takino T (2010) Coordinate action of membrane-type matrix metalloproteinase-1 (MT1-MMP) and MMP-2 enhances pericellular proteolysis and invasion. Cancer Sci 101: 843-847.
52. van Hinsbergh VW, Koolwijk P (2008) Endothelial sprouting and angiogenesis: matrix metalloproteinases in the lead. Cardiovasc Res 78: 203-212.
53. Xiong W, Knispel R, MacTaggart J, Greiner TC, Weiss SJ, et al. (2009) Membrane-type 1 matrix metalloproteinase regulates macrophage-dependent elastolytic activity and aneurysm formation *in vivo*. J Biol Chem 284: 1765-1771.
54. Yana I, Sagara H, Takaki S, Takatsu K, Nakamura K, et al. (2007) Crosstalk between neovessels and mural cells directs the site-specific expression of MT1-MMP to endothelial tip cells. J Cell Sci 120: 1607-1614.
55. Cherepanoff S, McMenamin P, Gillies MC, Kettle E, Sarks SH (2010) Bruch's membrane and choroidal macrophages in early and advanced age-related macular degeneration. Br J Ophthalmol 94: 918-925.
56. Anderson DH, Mullins RF, Hageman GS, Johnson LV (2002) A role for local inflammation in the formation of drusen in the aging eye. Am J Ophthalmol 134: 411-431.
57. Hageman GS, Luthert PJ, Victor Chong NH, Johnson LV, Anderson DH, et al. (2001) An integrated hypothesis that considers drusen as biomarkers of immune-mediated processes at the RPE-Bruch's membrane interface in aging and age-related macular degeneration. Prog Retin Eye Res 20: 705-732.
58. Tsigkos S, Koutsilieris M, Papapetropoulos A (2003) Angiopoietins in angiogenesis and beyond. Expert Opin Investig Drugs 12: 933-941.
59. Zech JC, Pouvreau I, Cotinet A, Goureau O, Le Varlet B, et al. (1998) Effect of cytokines and nitric oxide on tight junctions in cultured rat retinal pigment epithelium. Invest Ophthalmol Vis Sci 39: 1600-1608.
60. Luna JD, Chan CC, Derevjanik NL, Mahlow J, Chiu C, et al. (1997) Blood-retinal barrier (BRB) breakdown in experimental autoimmune uveoretinitis: comparison with vascular endothelial growth factor, tumor necrosis factor α, and interleukin-1β-mediated breakdown. J Neurosci Res 49: 268-280.
61. Elner VM, Strieter RM, Elner SG, Baggiolini M, Lindley I, et al. (1990) Neutrophil chemotactic factor (IL-8) gene expression by cytokine-treated retinal pigment epithelial cells. Am J Pathol 136: 745-750.
62. Harhaj NS, Antonetti DA (2004) Regulation of tight junctions and loss of barrier function in pathophysiology. Int J Biochem Cell Biol 36: 1206-1237.

63. Sainson RC, Johnston DA, Chu HC, Holderfield MT, Nakatsu MN, et al. (2008) TNF primes endothelial cells for angiogenic sprouting by inducing a tip cell phenotype. Blood 111: 4997-5007.
