# Supplementary material for: Adhesion Failures Determine the Pattern of Choroidal Neovascularization in the Eye: A Computer Simulation Study
Source: PLoS Comput Biol. 2012 May 3;8(5):e1002440. doi: 10.1371/journal.pcbi.1002440 (PMC3342931; doi:10.1371/journal.pcbi.1002440)
Supplement: Text S2 — Simplifying Assumptions of Our Model. (PDF) [file pcbi.1002440.s012.pdf]

Text S2

Simplifying Assumptions of Our Model

1. *In vivo* cell and ECM adhesion properties vary across a retina. We neglect these inhomogeneities to simplify interpretation of our results and because our simulations only cover patches of 120×120 μm^2, a scale over which adhesion and attachment properties are fairly uniform *in vivo*. We can partially reconstruct the properties of CNV over larger retinal regions by combining smaller patches with appropriate adhesion properties.
2. Under normal conditions, RPE cells do not proliferate *in vivo*, though they can proliferate under pathological conditions. **RPE cells** do not proliferate in our model.
3. All **RPE cells** have the same volume.
4. Photoreceptors degenerate in advanced AMD. Modeled photoreceptors (**POS** and **PIS**) do not die.
5. Pathological conditions often gradually weaken both the epithelial adhesion and attachment of RPE cells to BrM as CNV progresses. We do not include these adhesion changes.
6. Since we represent neither the basal lamina of RPE cells nor the RBaM explicitly**,** we represent the combined biological adhesion of the RPE to its basement membrane and of the basement membrane to BrM by **junctional adhesion** between **RPE cells** and **BrM**.
7. Cell-cell and cell-ECM adhesion depends on the time after contact, since it requires the accumulation of transmembrane and cytoplasmic molecules at the contact surface. Our model assumes that **adhesion** is patent (functional) immediately on contact.
8. RPE cells are polarized. In our model **RPE cells** are not intrinsically polarized but do effectively polarize when organized in epithelia.
9. We select and activate a single **tip cell** manually, so that the location of the initial **tip** cell is the same in all simulation replicas.
10. **Tip cells** degrade **BrM** only during the first 24 **hours**. Since **tip cell** properties are quite generic, a **tip cell** could also represent a cell of another type, *e.g.*, a macrophages or an endothelial cell penetrating into the retina.
11. **Tip cells** differentiate into **stalk cells** after 24 **hours**, independent of environmental factors. **Stalk cells** at the leading edge of a sprout retain the stalk-guiding ability of tip cells.
12. *In vivo*, only a small population of endothelial cells have colony-forming potential. In our model all **stalk cells** have infinite proliferative potential.

13. We simplify the complex multi-layered structure of BrM into a passive one-layered **BrM**.
14. CNV is usually contained in a fibrovascular tissue. We do not model ECM synthesis and remodeling (except BrM breakdown) and ignore the fibrous components of other types of ECM.
15. We combine all vascular growth factors into two freely-diffusing **VEGF fields**, one fast diffusing and one slow diffusing, and neglect cell-membrane-bound and ECM-bound VEGF.
16. We assume that all **RPE-cell** surfaces uniformly secrete **RPE-derived VEGF-A** at a uniform rate depending on the level of **Oxygen partial pressure**.
17. **RPE** cells secrete **RPE-derived VEGF-A** at a higher rate when the **Oxygen partial pressure** drops below 49 mmHg (see the *Implementation Parameters* section in supplementary Text S3 for details).
18. We ignore passive and pumped fluid flow from the vitreous humor to the CC and its effects on VEGF and oxygen transport.
19. We represent inflammation only through its effects on adhesion and epithelial integrity.
20. We neglect inflammatory cells and their contribution to angiogenesis.
21. We ignore fluid accumulation, *e.g.* due to inflammation.
22. We model continuous light-adapted conditions rather than alternation between light-adapted and dark-adapted conditions.
23. We do not model blood flow or the effects of vessel diameter and length. We simplify oxygen transport by assuming a constant rate of **Oxygen** secretion throughout an **EC**'s volume.
