# Supplementary material for: Adhesion Failures Determine the Pattern of Choroidal Neovascularization in the Eye: A Computer Simulation Study
Source: PLoS Comput Biol. 2012 May 3;8(5):e1002440. doi: 10.1371/journal.pcbi.1002440 (PMC3342931; doi:10.1371/journal.pcbi.1002440)
Supplement: Text S3 — GGH Implementation of the Multi-cell Model. (PDF) [file pcbi.1002440.s013.pdf]

Text S3

Methods

The Glazier-Graner-Hogeweg Method

In the GGH, each simulated generalized **cell** consists of a domain of *lattice sites*, or *voxels*, at locations $\vec{i}$, sharing the same *cell index*, σ, in a ***cell-lattice***. Each **cell** has an associated ***cell type***, τ. Any material object, including biological cells, subcompartment of biological cells, ECM or fluid medium can be represented by one or more generalized **cells**. Our simulations employ several types of generalized **cell** which represent entire biological cells, two which represent clusters of compartments of biological cells (**PIS** and **POS**), one which represents the extracellular fluid medium (**medium**) and one which represents small amounts of BrM (**BrM**). The *Effective Energy* governs changes in the **cell-lattice** configuration. We describe the concentration of secreted morphogens and oxygen within the cells, medium and ECM macroscopically by concentration **fields** discretized at the resolution of the **cell-lattice**. **Fields** evolve according to partial-differential equations (*PDEs*) describing their diffusion, secretion, absorption and decay.

Motility, Labile adhesion, Volume and Surface Area

The basic GGH *Effective Energy* describes **cells** that have constrained volumes and surface areas interacting via **labile differential adhesion**:

$$H = \sum_{\substack{\vec{i},\vec{j} \\ \text{neighbors}}} J\left(\sigma\left(\vec{i}\right),\sigma\left(\vec{j}\right)\right)\left(1-\delta\left(\sigma\left(\vec{i}\right),\sigma\left(\vec{j}\right)\right)\right)+ \sum_{\substack{\sigma \\ \text{cells}}}\left[\lambda_{\text{vol}}\left(\sigma\right)\left(v\left(\sigma\right)-V_{\text{t}}\left(\sigma\right)\right)^2+\lambda_{\text{surf}}\left(\sigma\right)\left(s\left(\sigma\right)-S_{\text{t}}\left(\sigma\right)\right)^2\right], \quad (1)$$

where $v\left(\sigma\right)$ is the total number of lattice sites in **cell** σ, which is constrained to be close to the target volume $V_{\text{t}}\left(\sigma\right)$ (*i.e.*, deviation of $v\left(\sigma\right)$ from $V_{\text{t}}\left(\sigma\right)$ increases the *Effective Energy*), and $\lambda_{\text{vol}}\left(\tau\left(\sigma\right)\right)$ is the inverse compressibility of **cells** of type τ. Similarly, $\lambda_{\text{surf}}\left(\tau\left(\sigma\right)\right)\left(s\left(\sigma\right)-S_{\text{t}}\left(\sigma\right)\right)^2$ constrains the surface area of **cell** σ, $s\left(\sigma\right)$ to be close to the target surface area $S_t(\sigma)$. $J\left(\tau\left(\sigma\left(\vec{i}\right)\right),\tau\left(\sigma\left(\vec{j}\right)\right)\right)$ is the contact energy per unit area between **cells** $\sigma\left(\vec{i}\right)$ and $\sigma\left(\vec{j}\right)$. Higher (more positive) contact energies between **cells** result in greater repulsion

between the **cells** and lower (more negative) contact energies between **cells** result in greater adhesion between the **cells**. $2\lambda_{\text{vol}}\left(\tau(\sigma)\right)\left(V_{\text{t}}(\sigma)-v(\sigma)\right)$ is the internal pressure in **cell** σ.

To simulate the cytoskeletally-driven reorganization of **cells** and tissues, we model **cell** protrusions and retractions using a *modified Metropolis dynamics*. For each attempt of a **cell** to *displace* a neighbor, we select at random a **cell** boundary (*source voxel*) and a neighboring *target voxel* and calculate the change in the Effective Energy ΔH_{GGH}, if the source **cell** displaced the target **cell** at that voxel. If ΔH_{GGH} is negative, *i.e.*, the change is energetically favorable, we make it. If ΔH_{GGH} is positive, we accept the change with probability $e^{-\Delta H_{GGH}/T}$, where T describes the amplitude of cytoskeletal fluctuations in the **cells**. In our CNV simulations, all **cells** of a certain type have the same intrinsic motility $T\left(\tau(\sigma)\right)$. On a lattice with N sites, N displacement attempts represent our basic unit of time, one *Monte Carlo Step* (*MCS*). One MCS corresponds to 216 seconds (see *Simulation Parameters and Initial Configuration*).

We define a special, generalized **cell** representing the extracellular medium (**medium**). The total volume and surface area of **medium** are not constrained. **Medium** voxels can be both source voxels, *e.g.* during retraction of the trailing-edge of a **cell**, and target voxels, *e.g.* during formation of lamellipodia. Since **medium** does not have cytoskeletal fluctuations, we use the amplitude of cytoskeletal fluctuations of the non-**medium** target or source **cell** to determine the acceptance of a displacement involving **medium**.

We define a special, generalized **cell** representing a small volume of BrM (**BrM**). **BrM** generalized **cells** occupy one voxel and their total volume and surface area are constrained. We set both λ_{vol} and λ_{surf} for **BrM** very large and T for **BrM** to zero, so the ensemble of **BrM cells** behaves like a solid non-diffusing material. Biological BrM is modeled by a set of **BrM cells**/voxels arranged on a pair of flat quasi-two-dimensional layers, each one voxel thick.

Plastic Coupling

In the GGH model, **plastic coupling** simulates cytoskeletally-coupled junctional structures (*e.g.* TJs, AJs and desmosomes) as breakable linear springs that mechanically connect the centers-of-mass of neighboring **cells** and or of **cells** to adjacent regions of **BrM**:

$$H_{\text{plastic}}=\sum_{\text{links}}\lambda_{\text{plastic}}\left(\tau(\sigma),\tau(\rho)\right)\left(\ell(\sigma,\rho)-\ell_{\text{target}}(\sigma,\rho)\right)^2, \quad (2)$$

where $\ell(\sigma,\rho)$ is the distance between the centers-of-mass of the neighboring generalized **cells** σ and ρ, $\ell_{\text{target}}(\sigma,\rho)$ is a target length defining the unstretched distance between the objects and $\lambda_{\text{plastic}}(\tau(\sigma),\tau(\rho))$ is the spring constant of the links that depends on the type of generalized **cells** σ and ρ (Table 12). We calculate $\Delta H_{\text{plastic}}$ for each displacement attempt and add it to the change in the Effective Energy ΔH_{GGH}. For small displacements, equivalent to small stress, H_{plastic} constrains $\ell(\sigma,\rho)$ to be close to $\ell_{\text{target}}(\sigma,\rho)$. However, a link breaks whenever its $\ell(\sigma,\rho)$ reaches twice its target equilibrium (unstretched) length, so a material composed of generalized **cells** coupled by such links is *quasi-plastic* or *plastic*. The total number of such links for each **cell** is limited to a certain number depending on type of **cells** σ and ρ. For example, an **RPE** cells can form up to 6 links with its neighboring **RPE cells** and up to 6 links with **BrM**. When a link breaks both generalized **cells** to which it connected can form a new link with their neighbors with a probability, depending on an *activation energy* that we add to ΔH_{GGH}. Large negative activation energies increase the probability of formation new links. The target equilibrium lengths of new links are prespecified. We increase the target equilibrium length between **stalk cells** and other **ECs** as **stalk cells** grow (see *Growth, Proliferation and Death*).

Fields

We represent **RPE-derived VEGF-A** as a **field** $V_{\text{L}}(\vec{x})$ with units of molecules per μm^3, which diffuses with diffusion coefficient $D_{\text{VEGF}}^{\text{RPE}}$ everywhere and decays at a rate γ_{VEGF}. **RPE cells** secrete **RPE-derived VEGF-A** into $V_{\text{L}}(\vec{x})$ uniformly at all their voxels at a constant basal rate SV_{basal}^{RPE} (molecules per voxel per 216 seconds) during normoxia. During hypoxia (for a definition, see the *Simulation Parameters and Initial Configuration section* below) individual **RPE cells** secrete **RPE-derived VEGF-A** into the **RPE-derived VEGF-A field** at a higher rate, SV_{max}^{RPE} (molecules per voxel per 216 seconds). We assign hypoxic **RPE cells**, to the **HRPE cell type**. **Vascular cells** in our model take up **RPE-derived VEGF-A** from the concentration **field** uniformly at a constant rate QV_{EC} (molecules per voxel per 216 seconds). Both **stalk cells** and the **tip cell** take up twice as much **RPE-derived VEGF-A** per voxel per unit time as **vascular cells**. So $V_{\text{L}}(\vec{x})$ evolves according to:

$$\frac{\partial V_{\mathrm{L}}\left(\vec{x}\right)}{\partial t} = D_{\mathrm{VEGF}}^{\mathrm{RPE}}\,\nabla^2 V_{\mathrm{L}}\left(\vec{x}\right) - \gamma_{\mathrm{VEGF}}\,V_{\mathrm{L}}\left(\vec{x}\right) + S\left(\vec{x}\right) - Q\left(\vec{x}\right),$$
$$S\left(\vec{x}\right) = SV_{\mathrm{basal}}^{\mathrm{RPE}}\,\delta\left(\tau\left(\sigma\left(\vec{x}\right)\right),\mathbf{RPE}\right) + SV_{\mathrm{max}}^{\mathrm{RPE}}\,\delta\left(\tau\left(\sigma\left(\vec{x}\right)\right),\mathbf{HRPE}\right),$$
$$Q\left(\vec{x}\right) = QV_{\mathrm{EC}}\left(\delta\left(\tau\left(\sigma\left(\vec{x}\right)\right),\mathbf{CC}\right) + 2\,\delta\left(\tau\left(\sigma\left(\vec{x}\right)\right),\mathbf{CNV}\right)\right), \tag{3}$$

where $\delta\left(\tau\left(\sigma\left(\vec{x}\right)\right),\mathbf{RPE}\right)=1$ inside **RPE cells** and 0 elsewhere, $\delta\left(\tau\left(\sigma\left(\vec{x}\right)\right),\mathbf{HRPE}\right)=1$ inside **HRPE cells** and 0 elsewhere, $\delta\left(\tau\left(\sigma\left(\vec{x}\right)\right),\mathbf{CC}\right)=1$ inside **vascular cells** and 0 elsewhere and $\delta\left(\tau\left(\sigma\left(\vec{x}\right)\right),\mathbf{CNV}\right)=1$ inside **stalk cells** and the **tip cell** and 0 elsewhere.

We represent **short-diffusing VEGF-A** as a **field** $V_{\mathrm{S}}\left(\vec{x}\right)$ with units of molecule per μm^3, which diffuses at a constant rate $D_{\mathrm{VEGF}}^{\mathrm{EC}}$ everywhere and degrades at a constant rate γ_{VEGF} in the **medium**. **ECs** (**tip**, **stalk** and **vascular cells**) secrete **short-diffusing VEGF-A** into $V_{\mathrm{S}}\left(\vec{x}\right)$ uniformly at all their voxels at a constant rate SV^{EC}, so $V_{\mathrm{S}}\left(\vec{x}\right)$ evolves according to:

$$\frac{\partial V_{\mathrm{S}}\left(\vec{x}\right)}{\partial t} = D_{\mathrm{VEGF}}^{\mathrm{EC}}\,\nabla^2 V_{\mathrm{S}}\left(\vec{x}\right) - \gamma_{\mathrm{VEGF}}\,V_{\mathrm{S}}\left(\vec{x}\right) + SV^{\mathrm{EC}}\,\delta\left(\tau\left(\sigma\left(\vec{x}\right)\right),\mathrm{EC}\right), \tag{4}$$

where $\delta\left(\tau\left(\sigma\left(\vec{x}\right)\right),\mathbf{EC}\right)=1$ inside **vascular**, **stalk cells** and the **tip cell** and 0 elsewhere.

We represent partial pressure of **Oxygen** by a **field** $PO\left(\vec{x}\right)$ with units of mmHg, which diffuses at a constant rate D_{ox} everywhere. **Vascular cells** secrete **Oxygen** into the **Oxygen field** uniformly at all their voxels at a rate SC_{ox} (O_2 (100 g **CC** tissue min)$^{-1}$) that maintains $PO\left(\vec{x}\right)$ averaged over all voxels of the **CC** at 80 mmHg. **Stalk** cell of the **CNV**, secrete **Oxygen** uniformly at all their voxels at a rate NC_{ox} that maintains $PO\left(\vec{x}\right)$ averaged over all the voxels of **stalk cells** at 65 mmHg (for a detailed discussion see the *Oxygen Transport Parameters* section, below). **PIS cell-parts** take up **Oxygen** uniformly at a constant rate, QO_{PIS} with units of ml O_2 (min)$^{-1}$ per 100g of **PIS** tissue. Since the rate of diffusion of oxygen is fast compare to all other processes in the model, its time-dependent concentration is close to its stationary profile, so we simulate its transport using a stationary diffusion equation:

$$k\,D_{\text{ox}}\,\nabla^2 PO\left(\vec{x}\right)+SC_{\text{ox}}\,\delta\left(\tau\left(\sigma\left(\vec{x}\right)\right),\mathbf{CC}\right)+SN_{\text{ox}}\,\delta\left(\tau\left(\sigma\left(\vec{x}\right)\right),\mathbf{CNV}\right)$$
$$-QO_{\text{PIS}}\,\delta\left(\tau\left(\sigma\left(\vec{x}\right)\right),\mathbf{PIS}\right)=0, \tag{5}$$

where *k* is the solubility of oxygen per gram of retinal tissue and NC_{ox} and SC_{ox} are the amounts of oxygen secretion per unit time (ml O_2 (100 g tissue min)$^{-1}$, 1 MCS = 3.6 minutes) locally from the **CC** and **stalk cells**, respectively (see Table 8 for the parameters), $\delta\left(\tau\left(\sigma\left(\vec{x}\right)\right),\mathbf{CNV}\right)=1$ inside **stalk cells** and 0 elsewhere and $\delta\left(\tau\left(\sigma\left(\vec{x}\right)\right),\mathbf{CC}\right)=1$ inside the **CC** and 0 elsewhere.

We represent the **MMP** concentration as a **field** $M\left(\vec{x}\right)$ with units of molecule per μm^3, which diffuses at a constant rate D_{MMP} and degrades at a constant rate η_{MMP} everywhere. The **tip cell** secretes **MMP** into $M\left(\vec{x}\right)$ uniformly at all their voxels at a constant rate SM^{tip} (with units of molecules per voxel per 216 seconds), so $M\left(\vec{x}\right)$ evolves according to:

$$\frac{\partial M\left(\vec{x}\right)}{\partial t}=D_{\text{MMP}}\,\nabla^2 M\left(\vec{x}\right)-\gamma_{\text{MMP}}\,M\left(\vec{x}\right)+SM^{\text{tip}}\,\delta\left(\tau\left(\sigma\left(\vec{x}\right)\right),\mathbf{tip}\right), \tag{6}$$

where $\delta\left(\tau\left(\sigma\left(\vec{x}\right)\right),\mathbf{tip}\right)=1$ inside the **tip cell** and 0 elsewhere.

Chemotaxis

We include a chemotaxis term in the Effective Energy to model the VEGF-A response of ECs during angiogenesis. We add a saturated Savill-Hogeweg-type chemotaxis term with contact inhibition to the basic ΔH_{GGH} of the Effective Energy to represent the net effect of **ECs**' preferential formation of pseudopods in response to gradients of both **RPE-derived** and **short-diffusing VEGF-A** at **EC** boundaries with any generalized cell type except **ECs**:

$$\Delta H_{\text{chemotaxis}}=-\lambda_{\text{VS}}\left(V_{\text{S}}\left(\vec{i}_{\text{target}}\right)-V_{\text{S}}\left(\vec{i}_{\text{source}}\right)\right)-\lambda_{\text{VL}}\left(\frac{V_{\text{L}}\left(\vec{i}_{\text{target}}\right)}{V_{\text{L0}}+V_{\text{L}}\left(\vec{i}_{\text{target}}\right)}-\frac{V_{\text{L}}\left(\vec{i}_{\text{source}}\right)}{V_{\text{L0}}+V_{\text{L}}\left(\vec{i}_{\text{source}}\right)}\right), \tag{7}$$

where λ_{VS} and λ_{VL} are chemotaxis response coefficients and V_{L0} regulates saturation of the chemotaxis response to **RPE-derived VEGF-A**. Due to contact inhibition at **EC-EC** boundaries, $\Delta H_{\text{chemotaxis}}=0$. Thus the total change in the Effective Energy is:

$$\Delta H_{\text{total}} = \Delta H_{\text{GGH}} + \Delta H_{\text{chemotaxis}}. \tag{8}$$

The effects of the saturation of the chemotaxis response to **short-diffusing VEGF** on capillary formation have been studied elsewhere [1].

Growth, Proliferation and Death

As described in [2], growth of **stalk cells** is inhibited when the common contact area between **stalk cells** and other **ECs** is greater than a threshold. This threshold contact area is fixed and does not change as the **stalk cells** grow. We describe **stalk cell** growth and proliferation by increasing the target volume of **stalk cells** according to:

$$\frac{dV_{\text{t}}(\sigma)}{dt} = \frac{G_{\text{S}} V_{\text{L}}\left(\vec{i}_{\text{CM}}(\sigma)\right)}{V_0 + V_{\text{L}}\left(\vec{i}_{\text{CM}}(\sigma)\right)}, \tag{9}$$

when growth is not inhibited. We divide the volume of a **stalk cell** equally between two daughter **stalk cells** when the volume reaches the doubling volume, 1728 μm^3. G_S in units of $\mu m^3 \, min^{-1}$ is the *maximum growth rate*, V_0 is the concentration of **RPE-derived VEGF-A** at which the growth rate is half its maximum and $V_{\text{L}}\left(\vec{i}_{\text{CM}}(\sigma)\right)$ is the concentration of **RPE-derived VEGF-A** at the center-of-mass of the **stalk cell**. All voxels of a **stalk cell** (σ) take up diffusing **RPE-derived VEGF-A**. This uptake creates an intracellular gradient with a minimum concentration at the center of the **cell**. $V_{\text{L}}\left(\vec{i}_{\text{CM}}(\sigma)\right)$ is typically 10 times lower than the average $V_{\text{L}}\left(\vec{x}\right)$ at the boundary of the **cell**. We pick G_S so that **stalk cells** not touching other **ECs** divide every 18 hours if $V_{\text{L}}\left(\vec{i}_{\text{CM}}(\sigma)\right)$ is 0.1 pM.

As **stalk cells** grow, the equilibrium lengths of their **plastic** links with other **EC**s also increase. Since the distance between **stalk cells** in contact with **BrM** does not change as they grow, we do not increase the equilibrium lengths of the **plastic links** between **stalk cells** and **BrM** generalized **cells**. Because **stalk cells** are long and thin, their typical length grows as v rather than $v^{1/2}$:

$$\ell_{\text{target}}(\sigma,\rho) = \theta \times \left(v(\sigma) + v(\rho)\right), \tag{10}$$

where $v(\sigma)$ and $v(\rho)$ are the volumes of a **stalk cell** and an **EC**. θ is a constant that produces a $\ell_{\text{target}}(\sigma,\rho)$ roughly equal to the prespecified initial $\ell_{\text{target}}(\sigma,\rho)$ before **stalk-cell** growth. This growth in $\ell_{\text{target}}(\sigma,\rho)$ prevents **junctional adhesions** from restricting **cell** growth.

If at any time an **RPE cell** loses all contact both with other **RPE cells** and **BrM**, it dies. We set both the target volume and target surface area of the dying cell to zero. When its actual volume reaches 0, we remove the **cell**. Similarly, **ECs** die when the level of **RPE-derived VEGF-A** at their center-of-mass at any time falls below a threshold level, $V_{\mathrm{L}}^{\mathrm{T}}$. In our simulations, $V_{\mathrm{L}}^{\mathrm{T}}$ is 1000 times smaller than the level of **RPE-derived VEGF-A** at the center-of-mass of **vascular cells** of the **CC** in anatomically normal **CC-BrM-RPE-retina** (about 0.1 fM).

To model BrM degradation, we reduce the target volume of **BrM** voxels, $V_{\mathrm{t}}(\sigma)$, at a rate, G_B (the **BrM** *degradation rate*), proportional to concentration of the **MMP field** ($M(\vec{i})$) at that voxel:

$$\frac{dV_{\mathrm{t}}(\sigma)}{dt} = -G_B\, M(\vec{i}), \tag{11}$$

where G_B is a positive constant. We remove **BrM** voxels when their target volume ($V_{\mathrm{t}}(\sigma)$) becomes zero.

Simulation Parameters and Initial Configuration

The ratios of the parameter values in the Effective Energy in our simulations determine the relative amplitudes of different **cell** behaviors. *E.g.*, the strength of chemotaxis to **RPE-derived VEGF-A** relative to **RPE-RPE** and **RPE-BrM junctional adhesion** and **RPE-POS labile adhesion** determines the **CNV type** and dynamics. We chose to keep the chemotaxis strength of **ECs** constant and change the five adhesion strength parameters: 1) the **RPE-RPE labile adhesion** strength (*RRl*), 2) the **RPE-RPE plastic coupling** strength (*RRp*), 3) the **RPE-BrM labile adhesion** strength (*RBl*), 4) the **RPE-BrM plastic coupling** strength (*RBp*), 5) the **RPE-POS labile adhesion** strength (*ROl*).

Since many parameters in our GGH model have not yet been measured experimentally, we must fit them to match measurable aspects of **cell** behavior in our simulations to those observed in experiments. *E.g.*, the intrinsic cell motility ($T(\tau(\sigma))$) is not easy to measure experimentally, but the diffusion constant of cells in aggregates can be measured in both simulation and experiment and corresponds to a unique intrinsic motility ($T(\tau(\sigma))$) in a simulation. Similarly, we can determine the effective form and strength of the chemotaxis response in our simulations from experimental dose-response curves for cell migration in response to concentration gradients of a chemoattractant.

Geometrical Parameters

The retina has a quasi-two-dimensional geometry. We take the smaller dimension, which extends from the choroid to the inner layers of the retina as the *z*-axis and choose the *x* and *y* axes so the retinal layers lie parallel to the *xy*-plane. Since a normal retina has a fairly uniform structure in the direction of its extended dimensions, we assume that that the **cell** and **field** lattices are periodic in the *x* and *y* directions, but not in the *z* direction. RPE cells form an epithelium that is ~ 11 μm thick in the macula [3]. The diameter of RPE cells in the macula is typically ~ 15 μm [4]. Assuming a hexagonal shape for RPE cells, their total volume is ~ 1600 μm^3. Since ECs in capillaries have irregular shapes, measuring EC volume is difficult experimentally. We use cubic voxels with a volume of 27 μm^3 (a side of 3 μm). At this spatial resolution, **RPE cells** are large enough so discretization artifacts are tolerable. Simulated **RPE cells** and **ECs** have volumes of about 1728 μm^3 and 864 μm^3 respectively. Individual photoreceptors are about 2 μm in diameter and about 65 μm in length. We coarse-grain 2 of their 3 main compartments as **cell-parts**; **POS cell-part** have a volume of 32 × 32 × 32 μm^3 and **PIS cell-part** having a volume of 24 × 24 × 24 μm^3. Each **cell-part** represents a cluster of many photoreceptors. BrM thickness typically ranges from 2 μm in young adults to 4 μm in older adults. We build **BrM** out of two layer of small blocks, each of which of which has a volume of 27 μm^3, so the entire **BrM** has a volume of 120 × 120 × 6 μm^3. The total simulated volume is 120 × 120 × 78 μm^3. We assume that generalized **cells** have nearly the density of water.

We initialize our simulations with a simplified configuration representing a normal retina, where the **RPE** contacts and adheres to the **POS cell-parts** apically, adheres to neighboring **RPE cells** laterally and adheres to **BrM** basally (Figure 1). The **CC** forms a rectangular quasi-two-dimensional mesh that adheres to the outer side of **BrM**.

Effective Ranges of cell-cell, cell-CEM and cell-Field interactions

Endothelial cells use long thin fliopodial processes (from 5 μm to 100 μm in length) to explore their microenvironment, increasing their ability to find defects in the BrM-RPE-POS complex and regions with higher VEGF concentrations. The adhesive interactions of modeled **stalk cells** have a range of the sum of the **membrane fluctuation range** of 6 μm (2 **pixels**) and the **adhesion interaction range** of 6 μm (2 **pixels**). The range of chemotaxis interactions is the maximum **membrane fluctuation range** of 6 μm (2 **pixels**). Thus the simulated interaction ranges are smaller than the experimental ranges. However, this difference may be less significant than it first appears, because in our simulations the average thickness of the **RPE** layer is 12 μm, so a **stalk cell** next to **RPE** still can explore the **sub-retinal** space via both adhesion and chemotaxis, as in experiments.

Temporal Parameters and Cell Motility

We relate the simulation's MCS time-scale to seconds by comparing **cell** migration speeds in simulations to typical cell migration speeds in experiments. Bauer *et al* [5] estimated migration speeds of about 6 µm/day for invading angiogenic sprouts during tumor-induce angiogenesis. Growing stable capillaries in simulations restricts the average migration speeds of **stalk cells** to less than 0.005 voxels per MCS computed over 100 MCS intervals since faster **stalk cell** migration speeds produces unstable vasculature. Equating 6 µm/day to the maximal **stalk cell** speed of 0.005 voxel/MCS for a voxel size ~ 3 µm fixes the time-scale to 400 MCS ~ 24 hours or 1 MCS ~ 216 seconds.

Adhesion Parameters

We modeled the complex phenomena of epithelial adhesion between RPE cells, *i.e.* the ensemble of AJs, TJs, desmosome plaques and gap junctions, as **junctional adhesion**, a combination of **labile adhesion** at **cell** boundaries and **plastic coupling** between neighboring **RPE** cells. We explored adhesion impairments combinatorially with either one (*asymmetric*) or both (*symmetric*) components of the modeled **junctional adhesion** moderately or severely impaired. We can represent adhesion in aged or damaged RPE cells using symmetrical adhesion impairment, since all cellular processes are impaired in such cells. We can represent adhesion strength in young, but disturbed RPE cells, *e.g.* after sub-retinal injection in animals, using asymmetrical adhesion impairment. We are agnostic as to which junctional components asymmetric adhesion impairment affects. Indeed, multiple mechanisms could contribute to asymmetric impairment: 1) Microenvironmental conditions may differentially affect the strength of epithelial-junction components (AJs, TJs, …). For example, calcium-depletion greatly reduces the strength of AJs, but not of desmosome. We represent this class of adhesion impairment by moderately impaired or severely impaired **labile adhesion** and normal or moderately impaired **plastic coupling**, requiring that **labile adhesion** be more severely impaired than **plastic coupling**. 2) Different epithelial-junction components may assemble and disassemble at different rates. For example, when cultured epithelial cells reach confluency, N-cadherin accumulates at RPE-RPE contacts, after which N-cadherin organizes into AJ bands when as the cells form an epithelium. TJs and other junctional structures from later. In some diseases, young RPE cells express N-cadherin, but do not form other junctional components, so a normal RPE (epithelium) fails to form. We represent such condition by near-normal **labile adhesion** and impaired **plastic coupling** between **RPE** cells.

Bruch's Membrane Degradation Parameters

BrM is composed of multiple layers of different extracellular proteins. In general, different MMPs diffuse at different rates and degrade specific sets of extracellular proteins. We assume our **MMP** diffuse because membrane-bound MMPs activate diffusible extracellular MMPs. In our model, the **tip cell** degrades **BrM** by secreting a single type of **MMP**. Because **MMP** represents the degradation effects of multiple matrix degrading enzymes, the diffusion constant of the **MMP field**, D_{MMP} , and its degradation rate γ_{MMP} are not available experimentally. In our

model, we set the diffusion length of **MMP**, $L_{\mathrm{MMP}} = \sqrt{\frac{D_{\mathrm{MMP}}}{\gamma_{\mathrm{MMP}}}}$, and the **BrM** degradation rate, G_B, rate so the **tip cell** that secretes **MMP** degrades **BrM** locally and forms a roughly one cell-diameter hole in **BrM**. For a fixed G_B, a larger diffusion length of **MMP**, would produce a larger hole in **BrM**.

Oxygen Transport Parameters

Moving inward through the layers of the retina, the O_2 contribution of the choriocapillaries decreases and the contribution from the retinal capillaries increases [6-8]. Near the OLM both sources of oxygen contribute equally and the partial pressure of oxygen reaches its lowest level. Light-adapted photoreceptors consume less oxygen than dark-adapted photoreceptors. So dark-adaptation shifts the position of the O_2 minimum towards the ONL (Figure 1). We neglect this shift, since it negligibly affects the **Oxygen** partial pressure at the location of the **RPE cells**. We impose no-flux boundary condition for $PO\left(\vec{x}\right)$ at z = 0 and fix the **Oxygen** partial pressure at the **OLM** boundary to 18 mmHg (less than the average inner retinal oxygen partial pressure PO_2 ~ 20 mmHg) for both normoxia and hypoxia [7]. We fix PO_2 at the **choriocapillaris** to 80 mmHg [7].

Average experimentally-measured light-adapted and dark-adapted oxygen consumption rates are 2.6 and 5.2 ml O_2 (100 g tissue min)$^{-1}$ [9,10]. Since the PIS consumes nearly 100% of the oxygen and only occupies 20% of the retina by volume, the actual consumption rates of the PIS in light-adapted and dark-adapted conditions are about five times these average oxygen consumption rates: 13 and 26 ml O_2 (100 g tissue min)$^{-1}$ respectively (for a detailed discussion see [7]). We adopt the consumption rates estimated in [7], and model continuous light-adapted conditions rather than alternation between light-adapted and dark-adapted conditions.

In our simulations, we adjust the flux of **Oxygen** per unit volume (Table 8) transferred locally from the **choriocapillaries** to the **retina** to achieve an average $PO\left(\vec{x}\right)$ of 80 mmHg ($PO_{\mathrm{N}}^{\mathrm{CC}}$, averaged over voxels belong to the **CC**) under normoxia. For the parameter values in Table 8, the average **Oxygen** partial pressure at the center-of-mass of **RPE cells** under light-adapted conditions is 65 ± 3 mmHg. Direct measurement of oxygen partial pressure in the human retina is difficult experimentally; however, our predicted average **Oxygen** partial pressure at the center-of-mass of **RPE cells** is close to estimated oxygen partial pressures in humans, based on measured values in animals [6].

In our simulations, **stalk cells** forming **sub-RPE** or **sub-retinal** capillaries contribute little **Oxygen** to the **retina**. We adjust the flux of **Oxygen** per unit volume of **stalk cells** so the average PO_2 (averaged over all **stalk-cell** voxels) is the minimum of two PO_2 levels: 1) 65

mmHg, which is the average $PO(\vec{x})$ at the **RPE** in normal retina, 2) the highest level of $PO(\vec{x})$ on **RPE** side of **BrM**. This oxygen transport scheme ensures that the average PO_2 in **stalk cells** is always less than $PO(\vec{x})$ in the **CC**.

Experimentally, we do not know what levels of PO_2 trigger hypoxic signaling by RPE cells. Animals breathing 10% oxygen, experience mild systemic hypoxia. If we assume that biological RPE cells are hypoxic during systemic hypoxia, we can use our model to calculate the resulting $PO(\vec{x})$ in the RPE. Based on experimentally measured parameters, our simulations of normal retina predict that $PO(\vec{x})$ at the RPE decreases from ~ 65 mmHg to ~ 49 mmHg as the PO_2 at the CC decreases from 80 mmHg to 60 mmHg during systemic hypoxia in an anatomically normal retina under light-adapted condition. We assume that for **Oxygen** partial pressures less than 49 mmHg, **RPE cells** become hypoxic and secrete **RPE-derived VEGF-A** at the maximum rate $SV_{\max}^{RPE}$.

Quantification and Classification of Simulations

From the total contact areas between **stalk cells** and **BrM**, and between **stalk cells** and the **POS** we define the *morphometric weight*:

$$MW = \frac{(\textbf{stalk - BrM} \text{ contact area})}{(\textbf{stalk - BrM} \text{ contact area}) + (\textbf{stalk - POS} \text{ contact area})}. \tag{12}$$

The **stalk-BrM** contact area of a fully-developed capillary network is about the same as the **stalk-POS** contact area of a corresponding volume of fully-developed sub-retinal CNV. So, a *MW* close to 1 indicates that most **stalk cells** are confined between the **RPE** and **BrM** (**sub-RPE**) in **Type 1 CNV**. A *MW* close to 0 usually indicates that most **stalk cells** are confined between the **RPE** and **POS** (**sub-retinal**) in **Type 2 CNV**. A *MW* close to 0.5 indicates **Type 3 CNV**. For each snapshot of a simulation we use the *MW* to estimate the number of **sub-RPE** and **sub-retinal stalk cells**:

$$\begin{aligned} &\#\ \textbf{Sub - RPE stalk cells} = \text{total}\ \#\ \textbf{stalk cells} \times MW, \\ &\#\ \textbf{Sub - retinal stalk cells} = \text{total}\ \#\ \textbf{stalk cells} \times (1 - MW), \end{aligned} \tag{13}$$

We can calculate *MW* even when **CNV** fails to initiate, so the *MW* allows us to determine the **CNV** loci, even when CNV initiation fails. To classify **CNV** progression during a simulated **year**, we determine the ***early*** and ***late*** loci of the **stalk cells**, using the weighted mean *MW*s during the first or last three **months** of each simulation:

$$\langle MW \rangle = \frac{\sum_{i=1}^{N} S_i MW_i}{\sum_{i=1}^{N} S_i}, \quad (14)$$

where N is the total number of snapshots recorded during the first or last three **months**, as appropriate, S_i is total number of stalk cells in ith snapshot and MW_i is the morphometric weight of the ith snapshot.

Stalk cells in simulations that exhibit **Stable Type 3 CNV** often simultaneously touch **BrM** basally, touch **RPE cells** laterally and touch the **POS** apically, so they are contained neither in the **sub-RPE** space nor in the **sub-retinal** space.

If the **RPE** detaches completely from **BrM**, the **stalk-BrM** contact area decreases to zero, so $MW = 0$, incorrectly suggesting that all **stalk cells** are in the **sub-retinal** space. Thus in case of **RPE** detachment, we must invoke other measurements beyond the MW to characterize the **CNV**. We do not consider situations with **RPE** detachment in this paper.

Statistical Methods

We used the JMP statistical analysis package [11] to perform multiple-regressions to relate **CNV** initiation probabilities, types and dynamics to the underlying adhesion scenarios.

References

1. Merks RM, Perryn ED, Shirinifard A, Glazier JA (2008) Contact-inhibited chemotaxis in *de novo* and sprouting blood-vessel growth. PLoS Comput Biol 4: e1000163.
2. Shirinifard A, Gens JS, Zaitlen BL, Poplawski NJ, Swat M, et al. (2009) 3D multi-cell simulation of tumor growth and angiogenesis. PLoS One 4: e7190.
3. Spraul CW, Lang GE, Grossniklaus HE, Lang GK (1999) Histologic and morphometric analysis of the choroid, Bruch's membrane, and retinal pigment epithelium in postmortem eyes with age-related macular degeneration and histologic examination of surgically excised choroidal neovascular membranes. Surv Ophthalmol 44: 10-32.
4. Roorda A, Zhang Y, Duncan JL (2007) High-resolution in vivo imaging of the RPE mosaic in eyes with retinal disease. Invest Ophthalmol Vis Sci 48: 2297-2303.
5. Bauer AL, Jackson TL, Jiang Y (2007) A cell-based model exhibiting branching and anastomosis during tumor-induced angiogenesis. Biophys J 92: 3105-3121.
6. Wangsa-Wirawan ND, Linsenmeier RA (2003) Retinal oxygen: fundamental and clinical aspects. Arch Ophthalmol 121: 547-557.

7. Haugh LM, Linsenmeier RA, Goldstick TK (1990) Mathematical models of the spatial distribution of retinal oxygen tension and consumption, including changes upon illumination. Ann Biomed Eng 18: 19-36.
8. Linsenmeier RA, Braun RD (1992) Oxygen distribution and consumption in the cat retina during normoxia and hypoxemia. J Gen Physiol 99: 177-197.
9. Roos MW (2007) Theoretical estimation of retinal oxygenation during retinal detachment. Comput Biol Med 37: 890-896.
10. Roos MW (2004) Theoretical estimation of retinal oxygenation during retinal artery occlusion. Physiol Meas 25: 1523-1532.
11. Lehman A (2005) JMP for basic univariate and multivariate statistics: a step-by-step guide: SAS Publishing.
12. Sarks SH, Arnold JJ, Killingsworth MC, Sarks JP (1999) Early drusen formation in the normal and aging eye and their relation to age related maculopathy: a clinicopathological study. Br J Ophthalmol 83: 358-368.
13. Spraul CW, Grossniklaus HE (1997) Characteristics of drusen and Bruch's membrane in postmortem eyes with age-related macular degeneration. Arch Ophthalmol 115: 267-273.
14. Sarks JP, Sarks SH, Killingsworth MC (1994) Evolution of soft drusen in age-related macular degeneration. Eye (Lond) 8: 269-283.
15. Sarks SH, Van Driel D, Maxwell L, Killingsworth M (1980) Softening of drusen and subretinal neovascularization. Trans Ophthalmol Soc UK 100: 414-422.
16. Arnold JJ, Sarks SH, Killingsworth MC, Sarks JP (1995) Reticular pseudodrusen. A risk factor in age-related maculopathy. Retina 15: 183-191.
17. Zweifel SA, Imamura Y, Spaide TC, Fujiwara T, Spaide RF (2010) Prevalence and significance of subretinal drusenoid deposits (reticular pseudodrusen) in age-related macular degeneration. Ophthalmology 117: 1775-1781.
18. Cohen SY, Dubois L, Tadayoni R, Delahaye-Mazza C, Debibie C, et al. (2007) Prevalence of reticular pseudodrusen in age-related macular degeneration with newly diagnosed choroidal neovascularisation. Br J Ophthalmol 91: 354-359.
19. Spaide RF (1999) Choroidal neovascularization in younger patients. Curr Opin Ophthalmol 10: 177-181.
20. Johnson NF, Foulds WS (1977) Observations on the retinal pigment epithelium and retinal macrophages in experimental retinal detachment. Br J Ophthalmol 61: 564-572.
21. Kaur C, Foulds WS, Ling EA (2008) Blood-retinal barrier in hypoxic ischaemic conditions: basic concepts, clinical features and management. Prog Retin Eye Res 27: 622-647.
22. Cousins SW, Espinosa-Heidmann DG, Alexandridou A, Sall J, Dubovy S, et al. (2002) The role of aging, high fat diet and blue light exposure in an experimental mouse model for basal laminar deposit formation. Exp Eye Res 75: 543-553.
23. Orzalesi N, Migliavacca L, Miglior S (1994) Subretinal neovascularization after naphthalene damage to the rabbit retina. Invest Ophthalmol Vis Sci 35: 696-705.
24. Baba T, Bhutto IA, Merges C, Grebe R, Emmert D, et al. (2010) A rat model for choroidal neovascularization using subretinal lipid hydroperoxide injection. Am J Pathol 176: 3085-3097.
25. Kimura H, Sakamoto T, Hinton DR, Spee C, Ogura Y, et al. (1995) A new model of subretinal neovascularization in the rabbit. Invest Ophthalmol Vis Sci 36: 2110-2119.
26. Qiu G, Stewart JM, Sadda S, Freda R, Lee S, et al. (2006) A new model of experimental subretinal neovascularization in the rabbit. Exp Eye Res 83: 141-152.

27. Ni M, Holland M, Jarstadmarken H, De Vries G (2005) Time-course of experimental choroidal neovascularization in Dutch-Belted rabbit: clinical and histological evaluation. Exp Eye Res 81: 286-297.
28. Oshima Y, Oshima S, Nambu H, Kachi S, Hackett SF, et al. (2004) Increased expression of VEGF in retinal pigmented epithelial cells is not sufficient to cause choroidal neovascularization. J Cell Physiol 201: 393-400.
29. Bagci AM, Shahidi M, Ansari R, Blair M, Blair NP, et al. (2008) Thickness profiles of retinal layers by optical coherence tomography image segmentation. Am J Ophthalmol 146: 679-687.
30. Pogue BW, Paulsen KD, O'Hara JA, Wilmot CM, Swartz HM (2001) Estimation of oxygen distribution in RIF-1 tumors by diffusion model-based interpretation of pimonidazole hypoxia and Eppendorf measurements. Radiat Res 155: 15-25.
31. Shahidi M, Wanek J, Blair NP, Mori M (2009) Three-dimensional mapping of chorioretinal vascular oxygen tension in the rat. Invest Ophthalmol Vis Sci 50: 820-825.
32. Serini G, Ambrosi D, Giraudo E, Gamba A, Preziosi L, et al. (2003) Modeling the early stages of vascular network assembly. EMBO J 22: 1771-1779.
33. Chen R, Silva E, Yuen W, Brock A, Fischbach C, et al. (2007) Integrated approach to designing growth factor delivery systems. FASEB J 21: 3896-3903.
34. Leith JT, Michelson S (1995) Secretion rates and levels of vascular endothelial growth factor in clone A or HCT-8 human colon tumour cells as a function of oxygen concentration. Cell Prolif 28: 415-430.
35. Mac Gabhann F, Ji JW, Popel AS (2006) Computational model of vascular endothelial growth factor spatial distribution in muscle and pro-angiogenic cell therapy. PLoS Comput Biol 2: e127.
