# Supplementary material for: Adhesion Failures Determine the Pattern of Choroidal Neovascularization in the Eye: A Computer Simulation Study
Source: PLoS Comput Biol. 2012 May 3;8(5):e1002440. doi: 10.1371/journal.pcbi.1002440 (PMC3342931; doi:10.1371/journal.pcbi.1002440)
Supplement: Text S4 — Modeling Terminology. (PDF) [file pcbi.1002440.s014.pdf]

Text S4

Modeling Terminology

Because terminology in biological modeling and simulation is often inconsistent, we would like to define certain key modeling concepts and their designations. We distinguish types of models by their *level, i.e.*, their degree of abstraction, and by the *length scales* which they primarily treat. At each abstraction level and length scale, any model requires at least the following: *objects* (the physical components to be described), *properties* (the intrinsic properties of the objects, like volume, location, shape, …), *processes* (how objects affect each other. Biologists often call processes mechanisms, behaviors or interactions), *dynamics* (how objects and processes change in time) and *initial conditions* (the initial identity, configuration and state of all objects and processes) (Figure S1, below).

In the context of experimental biology, the term *biological model* often refers to an *in vitro* or *in vivo* cell or animal experiment that exhibits a biological phenomenon. However, in our computational-biology context, a *biological model* is a qualitative (possibly complex) description of a set of hypothesized objects, processes and relationships, dynamics and initial conditions, developed to explain a biological phenomenon. Biologists employ such biological models when they form mental images which they construct by integrating results from many different types of experiment. A *mathematical model* is a formalized, **quantifiable** representation of a biological model, which embodies explicitly the rules governing the biological behavior in a quantitative form (often as sets of equations); a *computational model* is an implementation of a mathematical model in the form of algorithms, which use particular methods and sets of possibly method-dependent parameters, initial conditions and boundary conditions; a *simulation* is an *instance* of a computational model expressed as computer-executable code with specific parameter values (we often employ families of simulations to evaluate a model's response to changes in boundary conditions or initial conditions); and a *visualization* is a set of images presenting a selection of the data a simulation produces. In practice, when developing a model, we begin at the most abstract level, by building a biological model, then gradually add details to develop less abstract models. At each level of abstraction, we must explicitly state the simplifications and assumptions that we have made in addition to those already introduced. Finally, we compare our visualization results to experimental data and refine or correct our biological model and the corresponding models at each level as needed.

When we refer to a *model* without further qualification, we refer to an ensemble of corresponding biological, mathematical and computational models and their corresponding simulations and visualizations, *i.e.*, a particular set of hypotheses and their descriptions at all levels of abstraction.

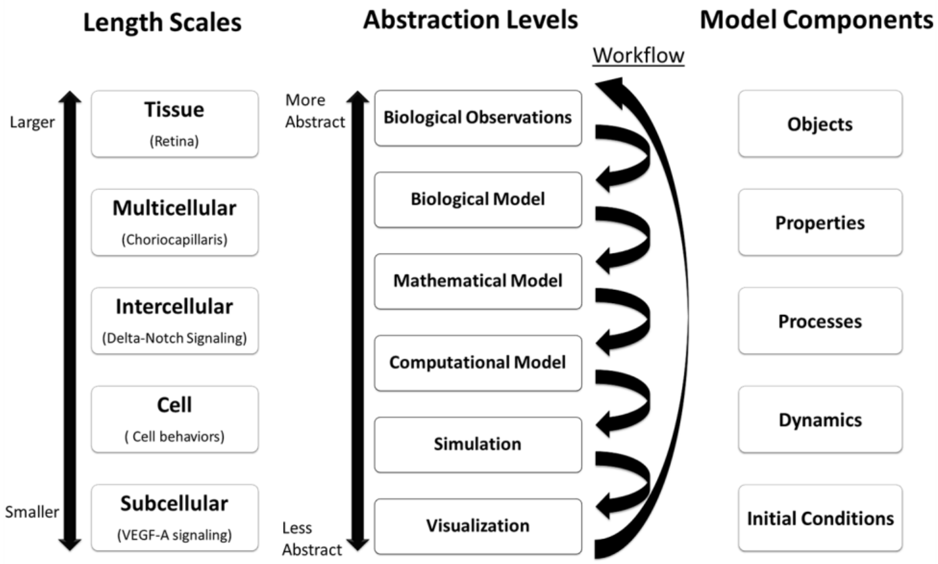

Figure S1. **Models and their components.** Left: A Multi-scale model includes processes and objects at multiple spatial **length scales** (with an illustrative example for each scale). Middle: Any model can be formulated at different **levels of abstraction**, from a verbal or schematic biological model to an algorithmic computation model which can be implemented as simulation code and visualized in a particular way. Model development begins at the most abstract level, by building a biological model, then gradually adding detail to develop less abstract models. Model development and validation involve continuous cycling from more abstract to less abstract levels, followed by comparison of visualization data with biological observations and model refinement. Right: To be complete and useful, all models at all spatial scales and levels of abstraction must include a basic set of **model components**: specifically what is modeled (objects), the capabilities of these objects, which include their behaviors and interactions (processes), how these objects and processes change in time (dynamics) and the situation modeled (initial conditions).
