# Supplementary material for: Adhesion Failures Determine the Pattern of Choroidal Neovascularization in the Eye: A Computer Simulation Study
Source: PLoS Comput Biol. 2012 May 3;8(5):e1002440. doi: 10.1371/journal.pcbi.1002440 (PMC3342931; doi:10.1371/journal.pcbi.1002440)
Supplement: Text S5 — CNV Progression Dynamics. (PDF) [file pcbi.1002440.s015.pdf]

Text S5

This supplementary text summarizes the time-development of **CNV** loci from **Early** to **Late CNV**.

CNV Progression Dynamics

Stable Type 1 CNV (S11): Early Type 1 → Late Type 1 CNV

In most adhesion scenarios that develop **Early Type 1 CNV** with *MW* > 90% the **CNV** remains in the **sub-RPE** space during one simulated **year** (**Late Type 1 CNV**, *MW* > 75%). *I.e.* they exhibit **Stable T1 CNV** (**S11 CNV**). **S11 CNV** occurs primarily when **RPE-BrM labile adhesion** is moderately to severely impaired ($RBl \leq 2$), **RPE-BrM plastic coupling** satisfies $RBl + RBp \leq 4$, and **RPE-RPE** and **RPE-POS labile adhesion** are both normal ($RRl = 3$ and $ROl = 3$). This class of scenarios corresponds to the first sub-class of adhesion scenarios prone to **ET1** (Table S2). Adhesion scenarios prone to **ET1 CNV** with severely impaired **RPE-RPE labile adhesion** ($RRl = 1$) (Table S2, ID: 83 and 84; both *MW* > 0.9) or with severely impaired **RPE-BrM labile adhesion** ($RBl = 1$) and **plastic coupling** ($RRp = 1$) (Table S2, ID: 75, 78, 81, 84; all *MW* > 0.9) do not remain stable, exhibiting **RPE** detachment and degeneration followed by **CNV** involution.

Multiple-regression analysis of the five adhesion parameters accounted for 76% of the observed variance in the probability of occurrence of **S11 CNV** in all 108 adhesion scenarios (adjusted $R^2 = 0.67$). Figure 6 shows the regression-inferred probability of occurrence of **S11 CNV** as a function of the five adhesion parameters, obtained by setting $RRp = RRl$ and $RBp = 3$. Severe impairment of either **RPE-POS labile adhesion** or **RPE-RPE labile adhesion** greatly reduces the *MW*, so **S11 CNV** can only occur when both adhesion strengths are near normal (Figure 6). The maximal regression-inferred probability of **S11 CNV** is 0.93 when **RPE-RPE junctional adhesion** is normal ($RRp = RRl = 3$), **RPE-BrM labile adhesion** is severely impaired ($RBl = 1$), **RPE-BrM plastic coupling** is normal ($RBp = 3$), and **RPE-POS labile adhesion** is normal ($ROl = 3$) (Figure 6). Severe impairment of both **RPE-BrM labile adhesion** and **plastic coupling** ($RBl = RBp = 1$) causes the **RPE** to detach from **BrM**, leading to either **T12 CNV** translocation or **T13 CNV** progression and causing **RPE** degeneration followed by **CNV** involution. The probability of **S11 CNV** for two of the adhesion scenarios in Table S5, ID: 3 and 41, is significantly larger than regression analysis predicts.

Generally, **CNV** *growth speed* differs from replica to replica in adhesion scenarios prone to **S11 CNV** (compare to **S22 CNV** dynamics, below). Figure 7 shows typical **S11 CNV** dynamics for 10 simulation replicas of a single adhesion scenario ($RRl = 3$, $RRp = 3$, $RBl = 2$, $RBp = 2$, $ROl = 3$) (Table S5, adhesion scenario ID: 38). We visualize snapshots of **S11 CNV** dynamics in one replica in Figure 8 and Movie S1. 9 of the ten simulation replicas initiate **CNV**, then develop **ET1 CNV** (Figure 8A, black arrows) and **S11 CNV** during one simulated **year** (Figure 7B and Figure 8D). Only 3 simulation replicas formed fully developed capillary networks composed of about 45 **stalk cells** (~ 3000 **cells**/mm^2) (Figure 7B and Figure 8D). In general when **stalk cells** form large aggregates the

concentration of **RPE-derived VEGF-A** at the center of the aggregate is less than the threshold below which **stalk cells** die. A few **stalk cells** in 5 of the simulation replicas die during one simulated **year** (Figure 7A). While **stalk cells** do contact the **POS** during the **early** window, **Type 2 CNV** does not develop (Figure 7C). The **RPE** remains viable and its total contact area with **BrM** decreases as **stalk cells** proliferate (Figure 7D-E). The **POS** never contacts **BrM**, indicating that the **RPE** does not develop any holes (Figure 7F and Figure 8A-D).

Sub-RPE to Sub-Retinal CNV Translocation (T12 Translocation): Early Type 1 → Late Type 2 CNV

Sub-RPE to **sub-Retinal** translocation occurs when **stalk cells** of **Early Type 1 CNV** ($MW \geq 0.75$) later translocate to the **sub-retinal** space to produce **Late Type 2 CNV** ($MW \leq 0.25$). **T12** translocation occurs primarily when **RPE-RPE labile adhesion** is normal ($RRl = 3$), both **RPE-BrM** and **RPE-POS labile adhesion** are severely impaired ($RBl = 1$ and $ROl = 1$), and the combination of **RPE-BrM** and **RPE-POS plastic coupling** satisfies $RRp + RBp \geq 4$, except for the case $RRp = RBp = 2$.

Adhesion scenarios in which some replicas exhibit **T12 CNV** can also have replicas which exhibit either **S22** or **S11** over one simulated **year**. Figure 9 shows **CNV** dynamics for 10 simulation replicas of the adhesion scenario ($RRl = 3$, $RRp = 3$, $RBl = 1$, $RBp = 1$, $ROl = 1$) (Table S6, adhesion scenario ID: 93). We visualize snapshots of the **T12 CNV** dynamics in one replica in Figure 10 and Movie S2. **CNV** initiates in all replicas; 8 replicas develop **ET1 CNV** (Figure 9A-B and Figure 10A). 7 replicas exhibit **T12 CNV**. We show snapshots of the **T12 CNV** dynamics that occur in one of those replicas in Figure 10. After 3 **months**, most replicas form a developed **sub-RPE** capillary network (black arrow, Figure 10A) composed of ~ 20 to 40 **stalk cells** (~ 1500 to 3000 **cells**/mm^2). One replica exhibits **S11 CNV**. Two replicas form **S22 CNV** (Figure 9C, black and dark red lines). The **RPE** remains viable in all replicas (Figure 9D). The contact area between the **RPE** and **BrM** decreases as **ET1 CNV** or **S11 CNV** develops, and remains constant during **ET2 CNV** (Figure 9E). **RPE** reattaches to **BrM** during **T12 CNV** (*e.g.* see the dark green line in Figure 9E). The **POS** never contacts **BrM**, indicating that the **RPE** does not develop any tears or holes (Figure 9F and Figure 10D).

Sub-RPE CNV to Sub-Retinal CNV Progression (P13 Progression): Early Type 1 → Late Type 3 CNV

In **sub-RPE CNV** to **sub-Retinal CNV** progression (**P13** progression), **stalk cells** initially grow between the **RPE** and **BrM** in **ET1 CNV** then invade the **sub-retinal** space to initiate **LT3**. **P13** progression primarily occurs when both **RPE-RPE** and **RPE-BrM labile adhesion** are severely impaired ($RRl = 1$ and $RBl = 1$), **RPE-BrM plastic coupling** strength is moderately to severely impaired ($RBp \leq 2$), and **RPE-POS labile adhesion** is normal ($ROl = 3$) (Table S7). In adhesion scenarios leading to **T13 CNV**, because both **RPE-RPE** and **RPE-BrM labile adhesion** are severely impaired, the **BrM-RPE-POS** complex can block **stalk cells** neither from invading the **sub-RPE** space nor the **sub-retinal** space. However, **stalk cells** consistently invade the **sub-RPE** space first and then progress to the **sub-retinal** space (Figure 11 and Figure 12). **Stalk cells** invade the **sub-retinal** space first primarily because of three mechanisms: 1) The **junctional**

adhesion by which **stalk cells** adhere to **BrM** is stronger than both **stalk-RPE** and **stalk-POS labile adhesion**. 2) Normal **RPE-POS labile adhesion** (*ROl* = 3) opposes **stalk cell** invasion of the **sub-retinal** space. 3) The gradient of **RPE-derived VEGF-A** in the apicobasal direction changes its direction from into the retina to out of the retina at the mid-plane of the **RPE** (the concentration of **RPE-derived VEGF-A** is maximal at the mid-plane of the **RPE**). Thus stalk cells entering the **sub-retinal** space across the **RPE** must migrate from regions with higher concentrations of **RPE-derived VEGF-A** to regions with lower concentrations, a migration opposed by chemotaxis.

Generally, **CNV** dynamics is very similar across all replicas in adhesion scenarios prone to the **P13 CNV** and much less heterogeneous than for **T12 CNV**. Figure 11 shows typical **P13 CNV** dynamics for 10 simulation replicas of the adhesion scenario (*RRl* = 1, *RRp* = 3, *RBl* = 1, *RBp* = 2, *ROl* = 3) (Table S7, adhesion scenario ID: 83). We visualize snapshots of **P13 CNV** dynamics in one replica in Figure 12 and Movie S3. **CNV** initiates in all replicas and all develop **ET1 CNV** (Figure 11A-B and Figure 12A). Between **months** 1 and 2, **stalk cells** (black outline arrow, Figure 12B) cross the **RPE** and invade the **sub-retinal** space once the number of **stalk cells** in the **sub-RPE** space reaches ~ 60 **cells** (Figure 11B-C). **CNV** progression into the **sub-retinal** space finishes around **month** 5 (Figure 11C and Figure 12C-D). A few **stalk cells** in most replicas die due to lack of **RPE-derived VEGF-A**. The **RPE** remains viable in all replicas (Figure 11D). The contact area between the **RPE** and **BrM** decreases as **ET1** develops, and remains constant afterwards during **LT3 CNV** (Figure 11E). The **POSs** do contact **BrM** a few times, but the contact area and duration are very small (Figure 11F), so the **RPE** does not develop any persistent or substantial holes (Figure 12D).

Stable Type 2 CNV (S22): Early Type 2 CNV → Late Type 2 CNV

In **Stable Type 2 CNV** (**S22 CNV**), **stalk cells** initially invade the **sub-retinal** space to develop **Early Type 2 CNV** and remain confined in the **sub-retinal** space in **Late Type 2 CNV**. The **ET2 CNV** classification is based on a $MW \leq 0.25$ (Table 3) during the first three months. Most adhesion scenarios that develop **ET2 CNV** in which the *MW* remains less than 0.15 during the first three months also exhibit **S22 CNV**. Thus, the three main classes of adhesion scenarios that cause **ET2 CNV** predominantly lead to **S22 CNV**. Table S3 (**ET2 CNV**) shows only adhesion scenarios with $MW < 0.05$ throughout the first three **months**, so some of the adhesion scenarios in Table S8 exhibiting **S22 CNV** are not listed in Table S3 (**ET2 CNV**).

Multiple-regression analysis of the five adhesivities for the probability of occurrence of **S22 CNV** accounted for 89% of the observed variance in the probability of occurrence of **S22 CNV** in all 108 adhesion scenarios (adjusted $R^2 = 0.84$). Figure 13 shows the regression-inferred probability of occurrence of **S22 CNV** as a function of the five adhesion parameters, obtained by setting *RRp* = *RRl* and *RBp* = *RBl*. The multiple-regression results show that moderate to severe impairment of **RPE-RPE junctional adhesion** (*RRp* = *RRl* < 2) and normal to moderately impaired **RPE-BrM junctional adhesion** (*RBp* = *RBl* > 2) develop **S22 CNV**, independent of the strength of **RPE-POS** adhesion (0.9 isosurface, Figure 13).

Generally, **CNV** dynamics is very similar across all replicas of the adhesion scenarios prone to **S22 CNV**. As for **P13 CNV**, the variability from replica to replica is smaller than for **S11 CNV**. Figure 14 shows typical **S22 CNV** dynamics for 10 simulation replicas of the adhesion scenario (*RRl* = 1, *RRp* = 1, *RBl* = 3, *RBp* = 3, *ROl* = 3) (Table S8, adhesion scenario ID: 16). We show snapshots of the **S22 CNV** dynamics in one replica in Figure 15 and Movie S4. **CNV** initiates in all replicas and all develop **ET2 CNV** (Figure 14A-B and Figure 15A-D). During first two **months** after initiation, **stalk cells** develop a capillary network in the **sub-retinal** space (Figure 15B and Figure 14C). **CNV** development in the **sub-retinal** space finishes around **month** 4 (Figure 14C and Figure 15C-D). A few **stalk cells** in most replicas die due to lack of **RPE-derived VEGF-A**. The **RPE** remains viable in all replicas (Figure 14D). The contact area between the **RPE** and **BrM** remains constant throughout **S22 CNV** (Figure 14E). The **POSs** do contact **BrM**, but the contact area and duration are small (Figure 14F), so the **RPE** does not develop any substantial or persistent holes (Figure 15D).

Sub-Retinal to Sub-RPE Progression (P23 CNV Progression): Early Type 2 CNV → Late Type 3 CNV

In **P23 CNV** progression, **stalk cells** initially invade the **sub-retinal** space to produce **Early Type 2 CNV**, then invade the **sub-RPE** space to progress to **Late Type 3 CNV**. **P23 CNV** primarily occurs when **RPE-RPE plastic coupling** is severely or moderately impaired (*RRp* $\leq$ 2) and all other adhesions are severely impaired (*RRl* = 1, *RBl* = 1, *RBp* = 1, *ROl* = 1).

Generally, **CNV** dynamics is very similar across all replicas of the adhesion scenarios prone to **P23 CNV**. Variability from replica to replica is low and comparable to the variability observed in **P13 CNV** and **S22 CNV**. Figure 16 shows typical **P23 CNV** dynamics for 10 simulation replicas of the adhesion scenario where all adhesions are severely impaired (*RRl* = 1, *RRp* = 1, *RBl* = 1, *RBp* = 1, *ROl* = 1) (adhesion scenario ID: 108). We visualize snapshots of the **P23 CNV** dynamics in one replica in Figure 17 and Movie S5. **CNV** initiates in all replicas and all replicas rapidly develop **ET2 CNV** (Figure 16C). **Stalk cells** cross the **RPE** and invade the **sub-RPE** space (Figure 16B and Figure 17A2) once the number of **stalk cells** in the **sub-retinal** space reaches ~ 50 **cells** which occurs during the first **month** after initiation (Figure 16C). **Stalk cells** gradually invade the **sub-RPE** space during the remainder of the simulated **year** (Figure 16B and Figure 17A2-D2). Unlike in previously discussed scenarios in which all **RPE cells** survive, **RPE cells** death increases with the number of **sub-RPE stalk cells** (Figure 16B). In two replicas 30 **cells** die (30% of the total of 100 **cells**) during the simulated **year** (Figure 16D). The contact area between the **RPE** and **BrM** decreases as **P23 CNV** develops (Figure 16E). In all replicas the **POS** contacts **BrM** persistently and extensively, as the **RPE** develops substantial holes (Figure 16F and Figure 17D1-2). Formation of a hole or tear in the **RPE** reduces its contact area with **BrM** (Figure 16F).

Stable Type 3 (S33 CNV): Early Type 3 CNV → Late Type 3 CNV

In stable **Type 3 CNV**, **stalk cells** initially invade both the **sub-RPE** and **sub-retinal** space and remain in both loci for the entire simulated **year**. **Stalk cells** occasionally migrate in both directions between the **sub-retinal** space and the **sub-RPE** space. **S33**

CNV occurs primarily for two classes of adhesion scenarios: 1) When **RPE-RPE labile adhesion** is severely impaired (*RRl* = 1), **RPE-POS labile adhesion** is normal (*ROl* = 3), **RPE-BrM labile adhesion** is moderately impaired (*RBl* = 2) and **RPE-BrM plastic coupling** satisfies $RBl + RBp \leq 4$. 2) When **RPE-RPE labile adhesion** is severely impaired (*RRl* = 1), **RPE-POS labile adhesion** is normal (*ROl* = 3), **RPE-BrM labile adhesion** is severely impaired (*RBl* = 1) and **RPE-BrM plastic coupling** is normal (*RBp* = 3). **RPE-RPE plastic coupling** has no effect on the probability of **CNV** initiation or occurrence of **S33 CNV** in these scenarios. **RPE-BrM junctional adhesion** in adhesion scenarios causing **S33 CNV** is less impaired than in those which result in **P13 CNV**. The greater **RPE-BrM junctional adhesion** encourages **stalk cells** to invade both the **sub-retinal** space and the **sub-RPE** space simultaneously (in **P13 CNV** all **stalk cells** invade the **sub-retinal** space first).

Generally, **CNV** dynamics is very similar across all replicas of the adhesion scenarios prone to **S33 CNV**. Variability from replica to replica is comparable to the variability in **P13 CNV**, **S22 CNV** and **P23 CNV**. Figure 18 shows the typical **S33 CNV** dynamics in 10 simulation replicas of the adhesion scenario (*RRl* = 1, *RRp* = 1, *RBl* = 2, *RBp* = 2, *ROl* = 3) (Table S10, adhesion scenario ID: 53). We visualize snapshots of **S33 CNV** dynamics in one replica in Figure 19 and Movie S33. **CNV** initiates in all replicas and all develop **ET3 CNV**. During the first **month**, more stalk cells invade the **sub-RPE** space than invade the **sub-retinal** space (Figure 18B-C and Figure 19A1). Between **months** 1 and 2, about 30% of the **sub-RPE stalk cells** transmigrate into the **sub-retinal** space (dark blue line, Figure 18B-C). After **month** 3, the number of **sub-RPE stalk cells** slowly increases, while the number of **sub-retinal stalk cells** remains constant. The contact area between the **RPE** and **BrM** rapidly decreases when **stalk cells** invade the **sub-RPE** space during the first **month** of the simulation, then rapidly increases as **sub-RPE stalk cells** transmigrate into the **sub-retinal** space between **months** 1 and 2. The contact area between the **RPE** and **BrM** slowly decreases during **months** 3 to 12. A few **RPE cell** die in most replicas, but **RPE cells** death is much less pervasive than in **P23 CNV**. In a few replicas the **POS** contacts **BrM** persistently, but the holes the **RPE** develops are significantly smaller than those occurring in **P23 CNV** (Figure 16F and Figure 17D1-2).
